# Supplementary figures and images for: The beneficial effect of chronic muscular exercise on muscle fragility is increased by Prox1 gene transfer in dystrophic mdx muscle
Source: PLoS One. 2022 Apr 18;17(4):e0254274. doi: 10.1371/journal.pone.0254274 (PMC9015141; doi:10.1371/journal.pone.0254274)

## Electrophoresis

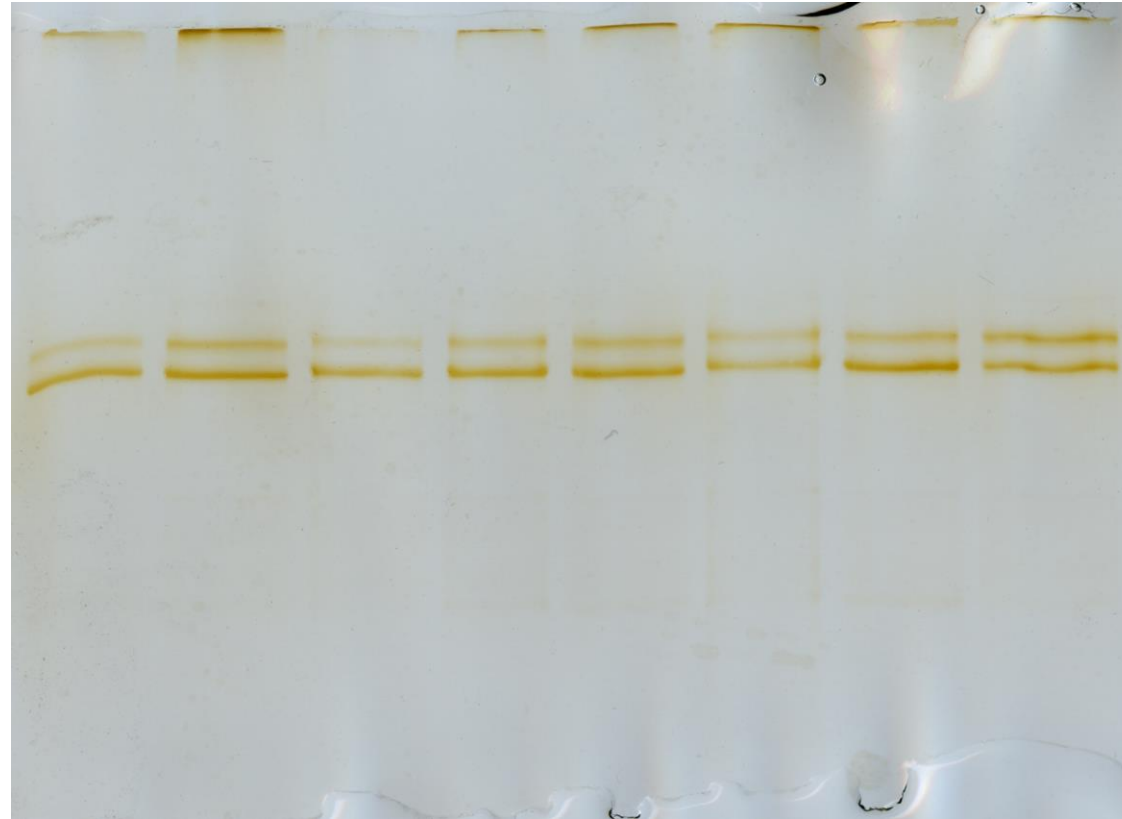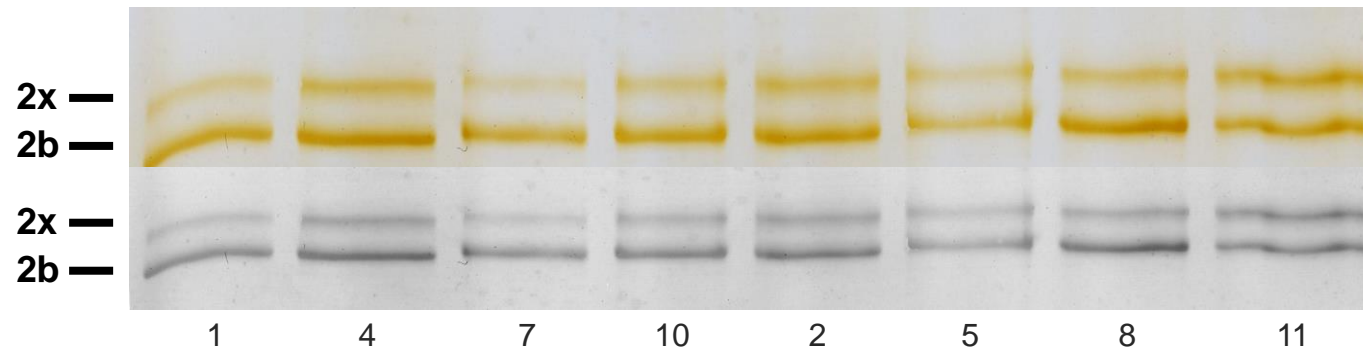

- 1- mdx
- 2- mdx
- 3- mdx
- 4- mdx + P
- 5- mdx + P
- 6- mdx + P
- 7- mdx + W
- 8- mdx + W
- 9- mdx + W
- 10- mdx + W + P
- 11- mdx + W + P
- 12- mdx + W + P

Supplement: S1 Fig — (PDF) [file pone.0254274.s001.pdf]

TA 1G

Mdx+P

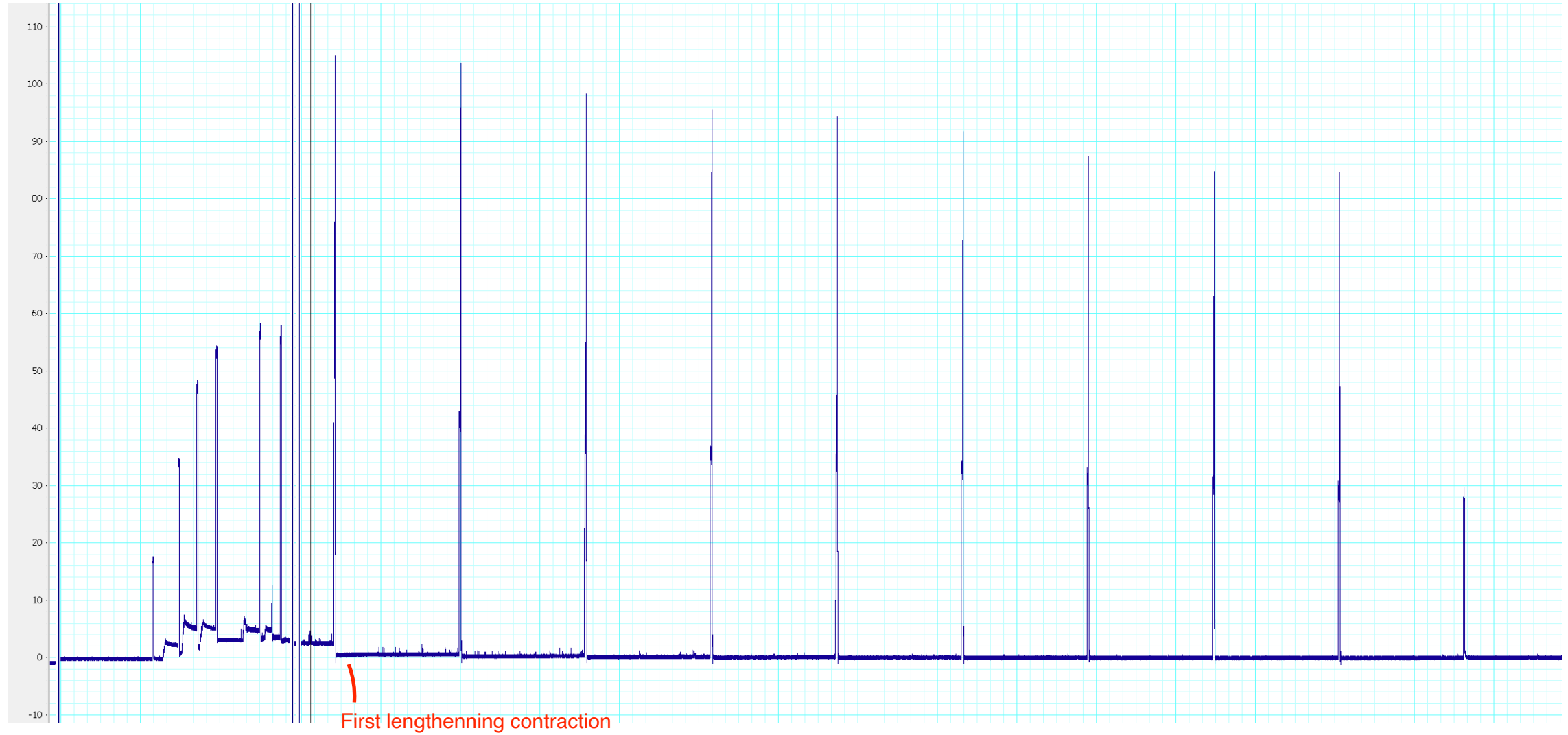

TA 2D

Mdx

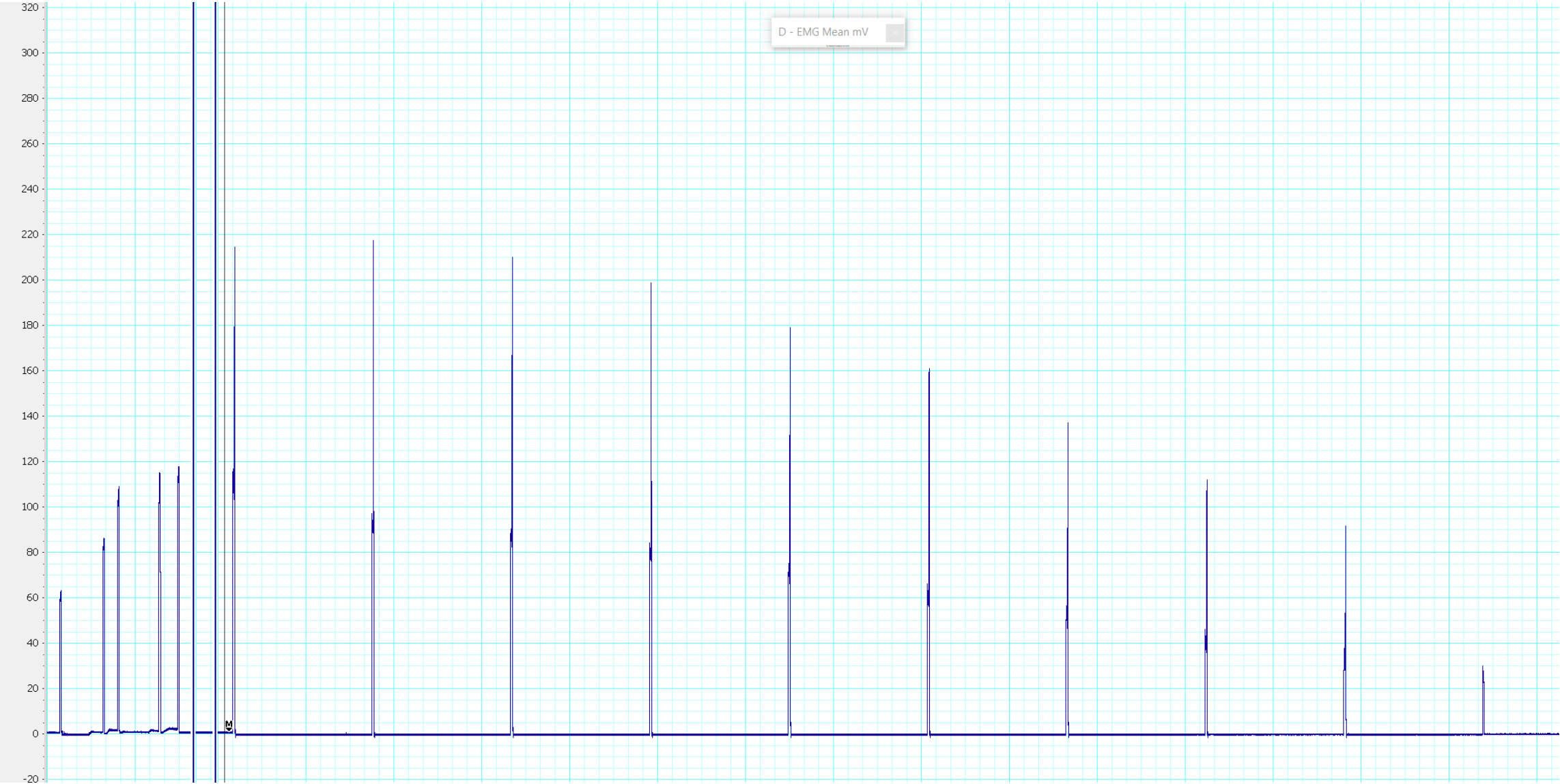

TA 2G

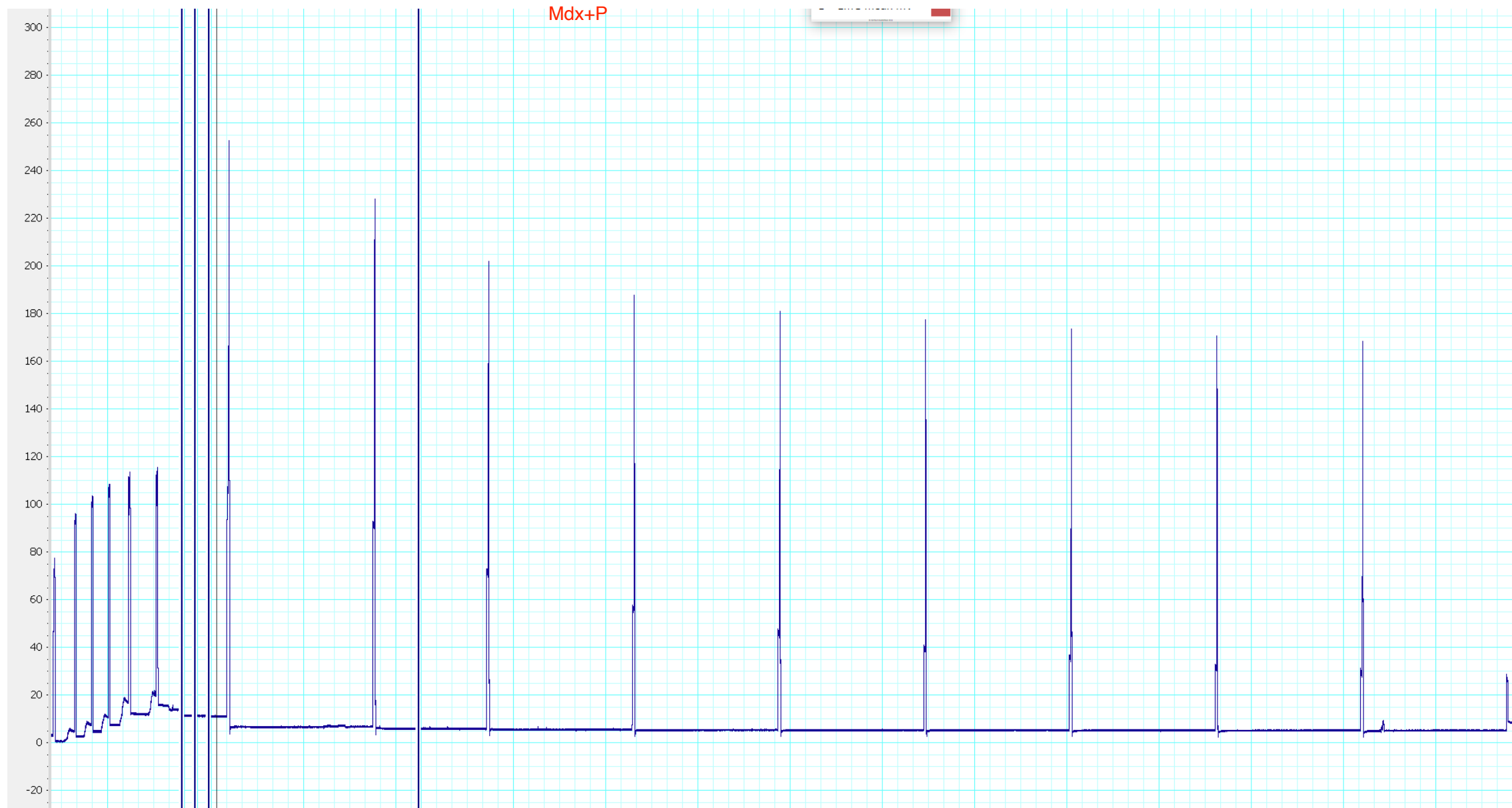

## SET 2

TA 4D

Mdx

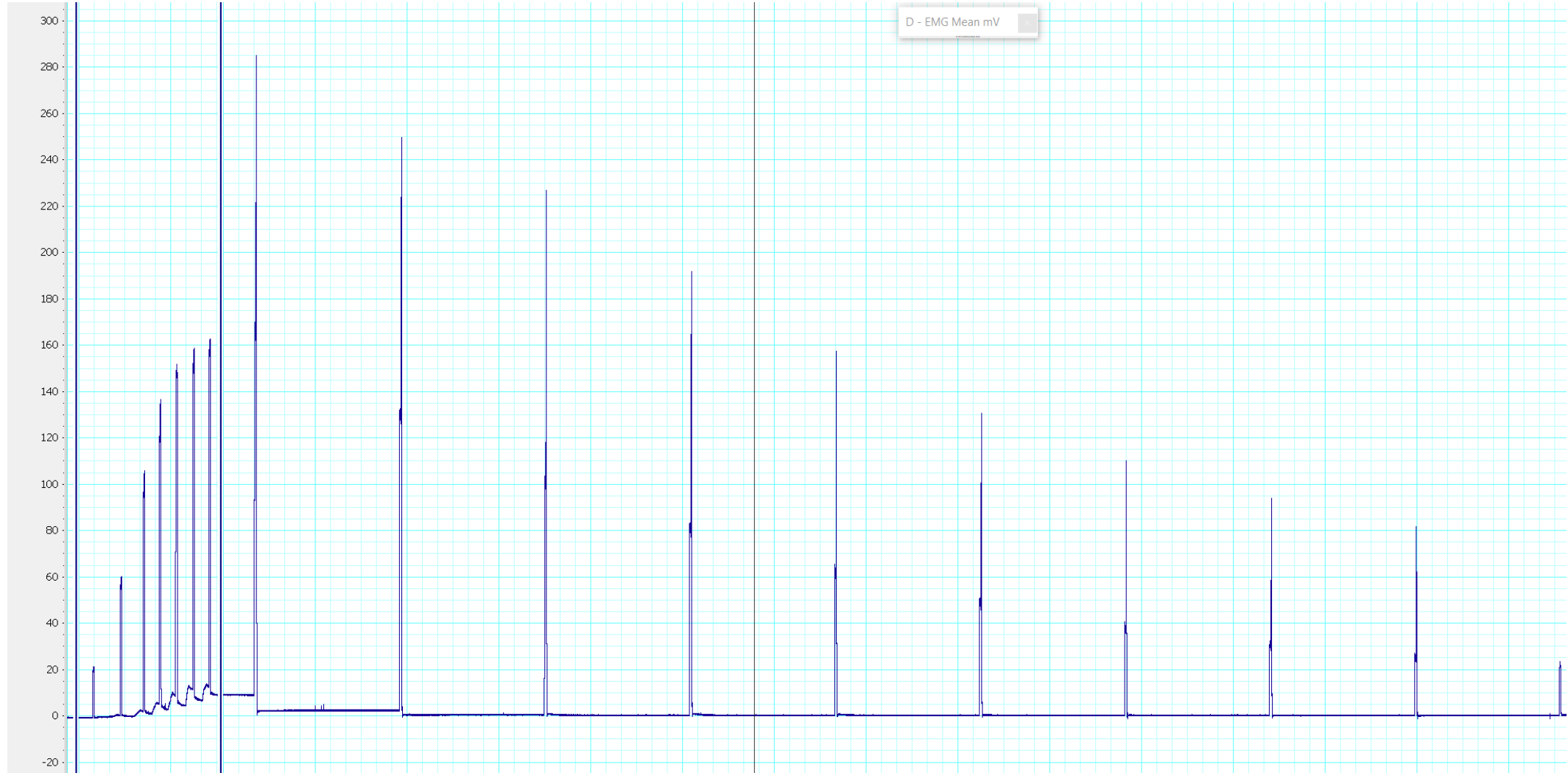

TA 4G

Mdx+P

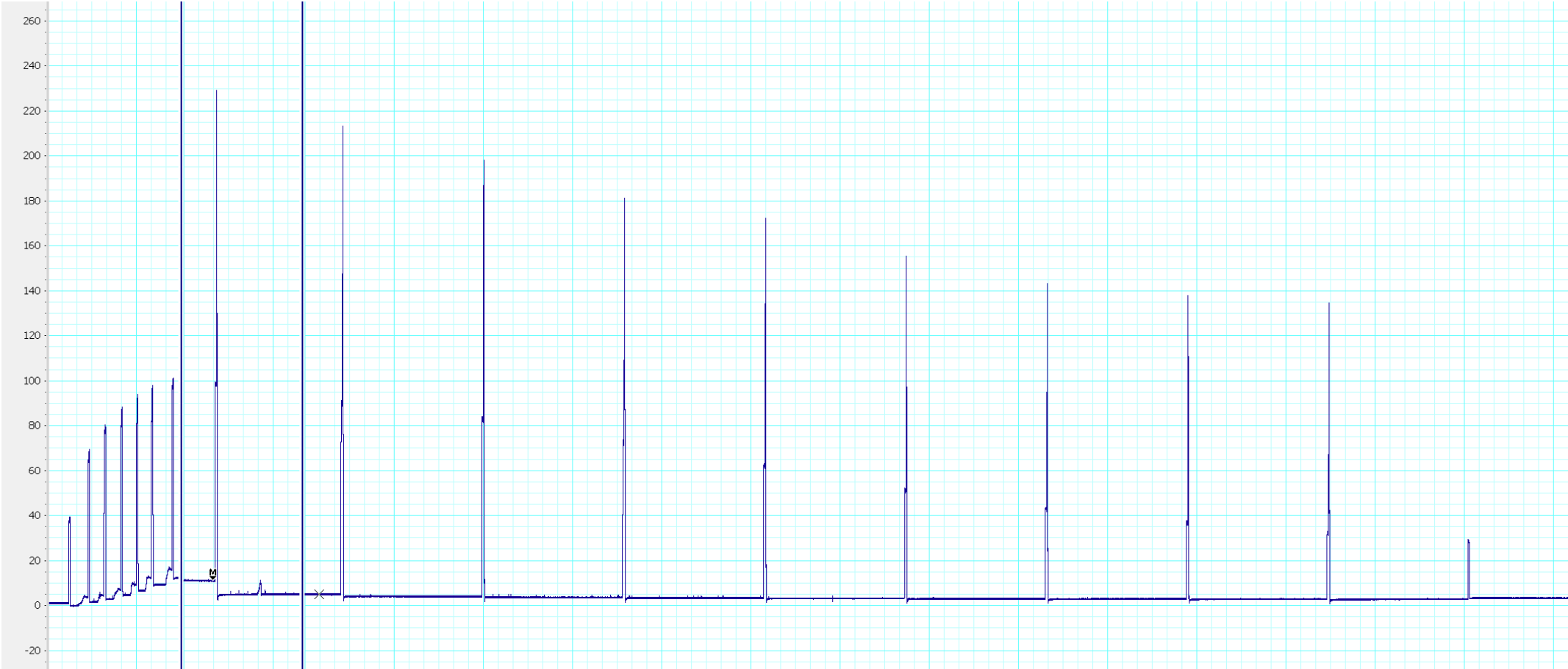

TA 5D

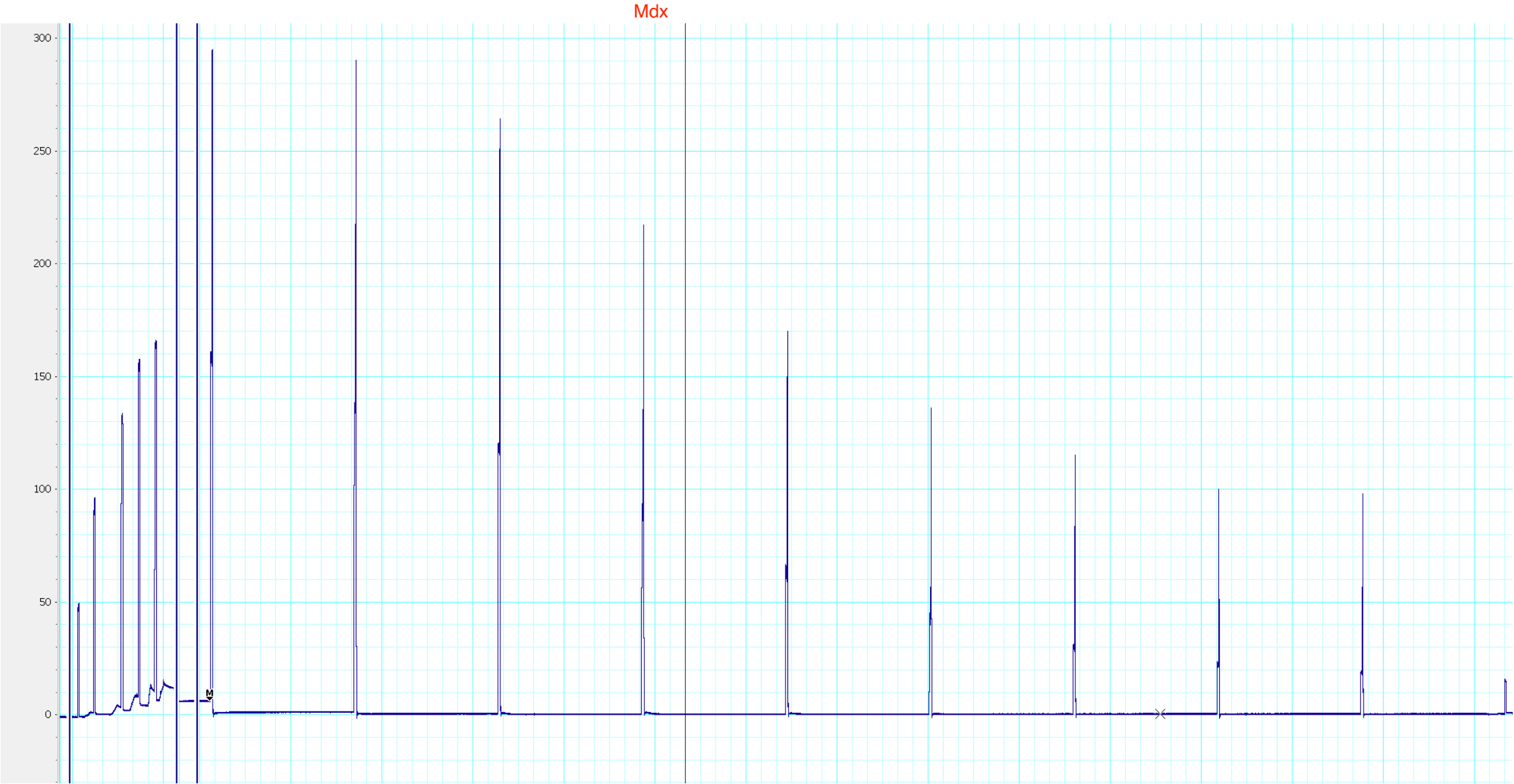

TA 5G

Mdx+P

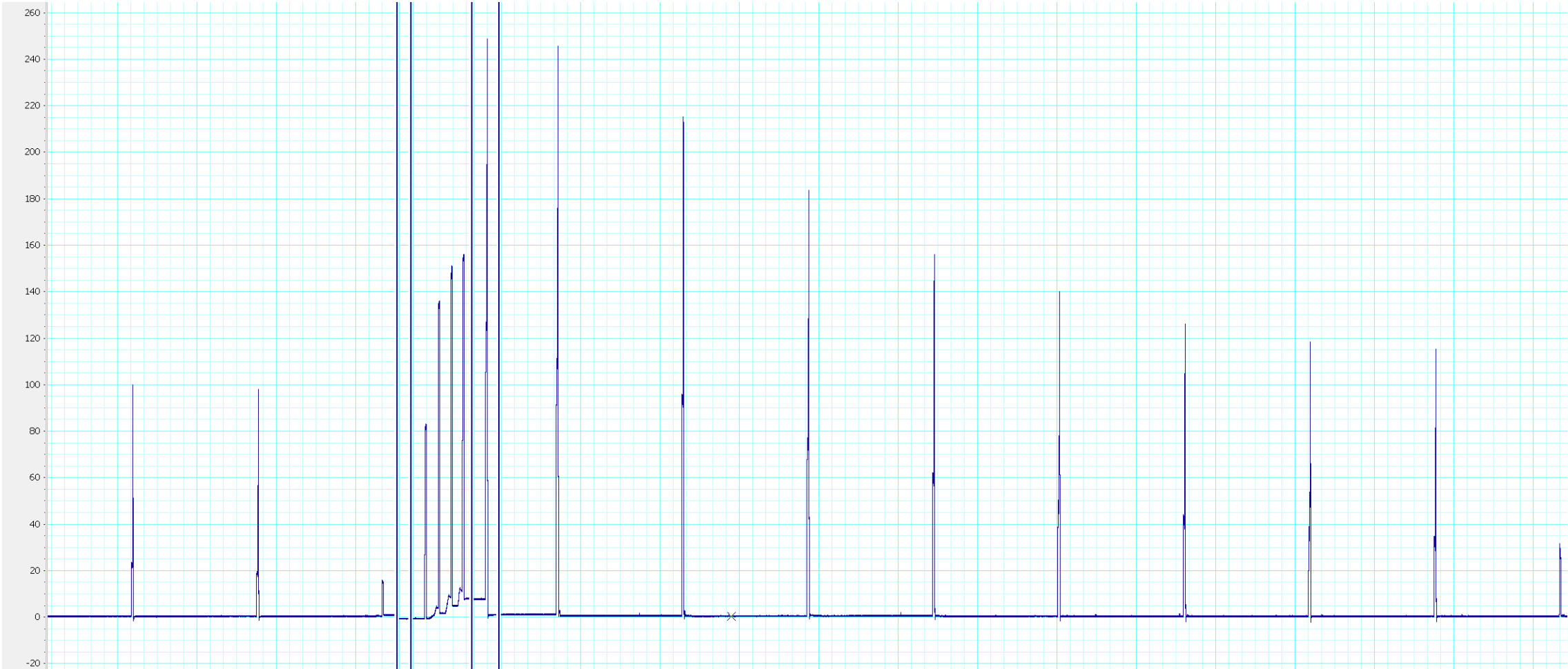

TA 6D

Mdx

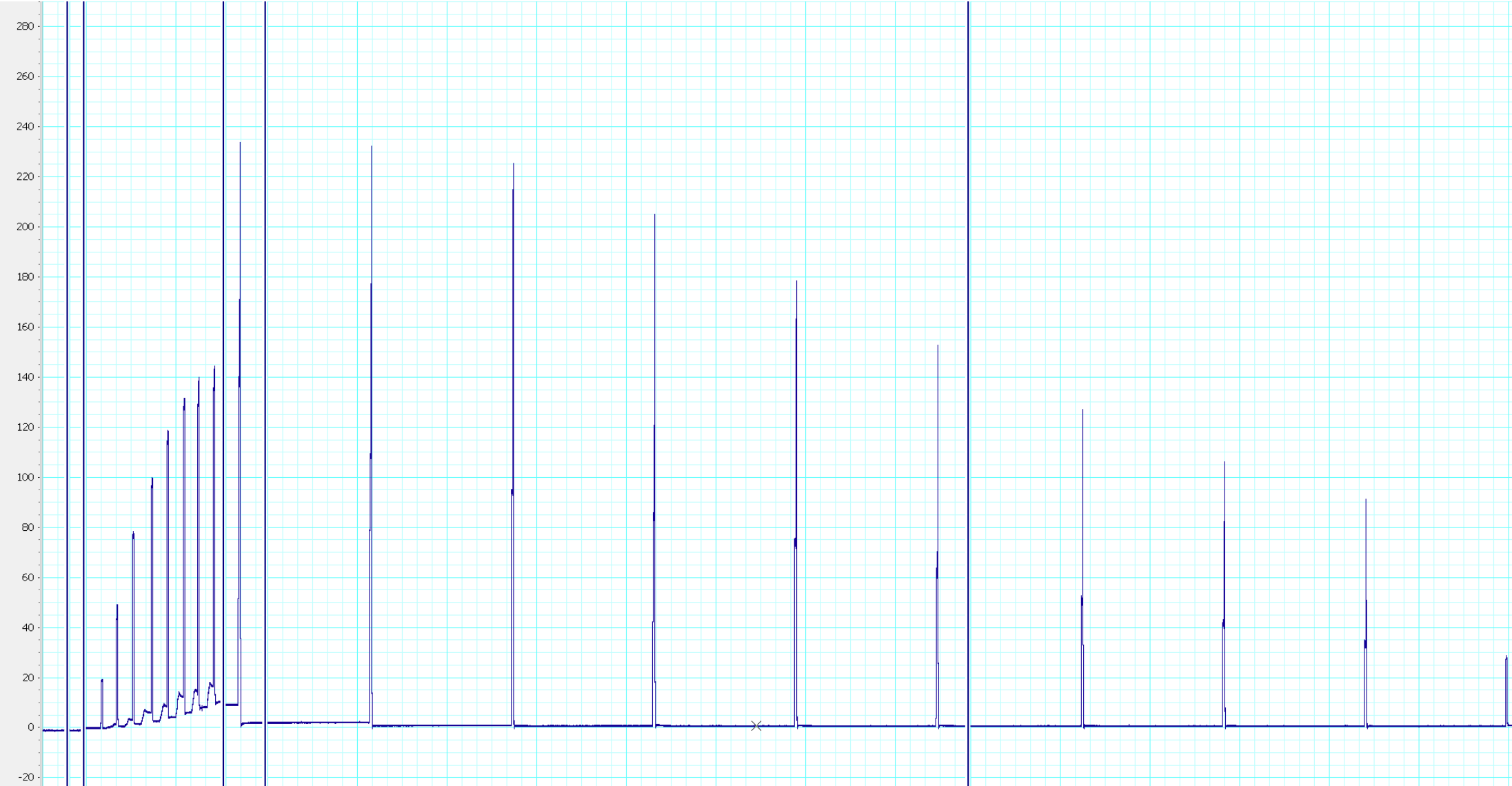

TA 6G

Mdx+P

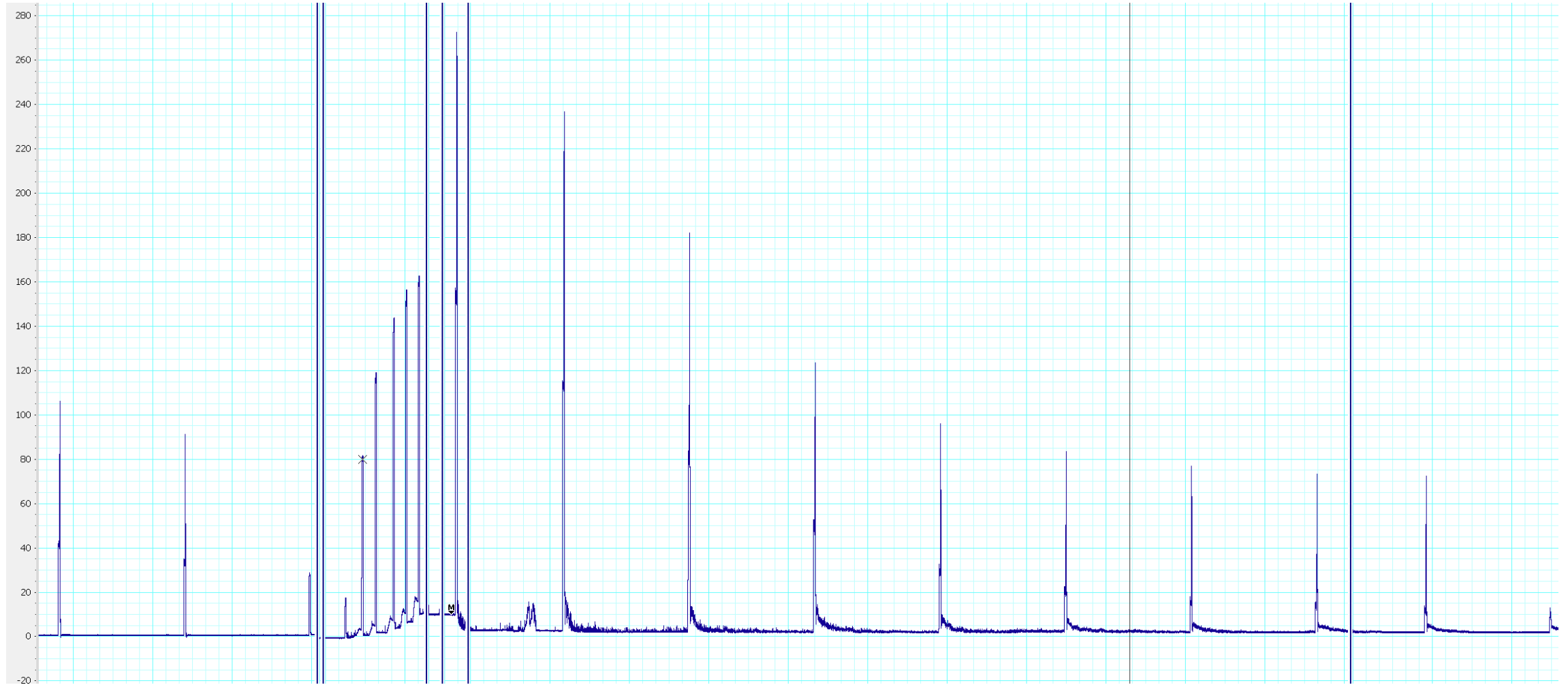

TA 8D

Mdx

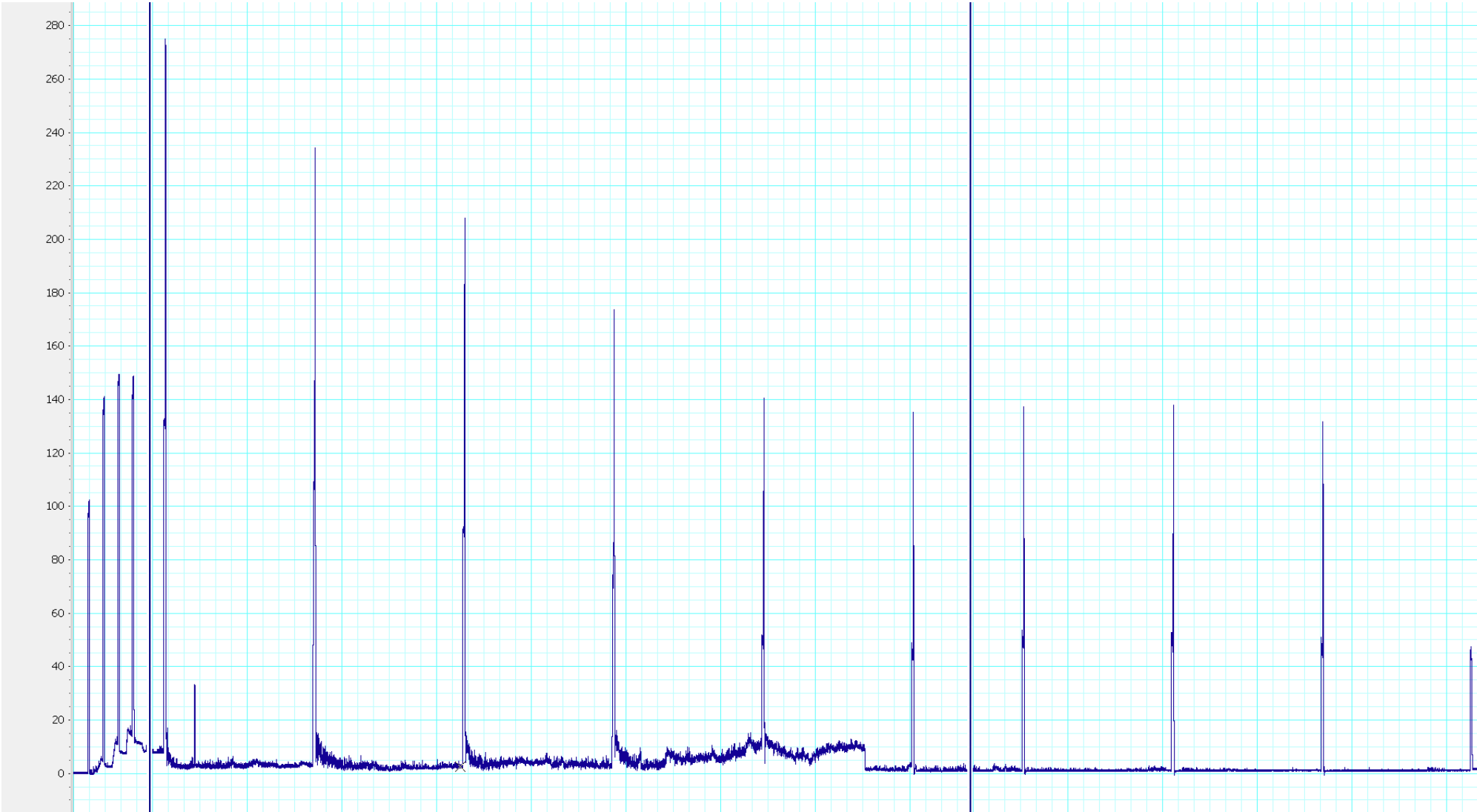

TA 8G

Mdx+P

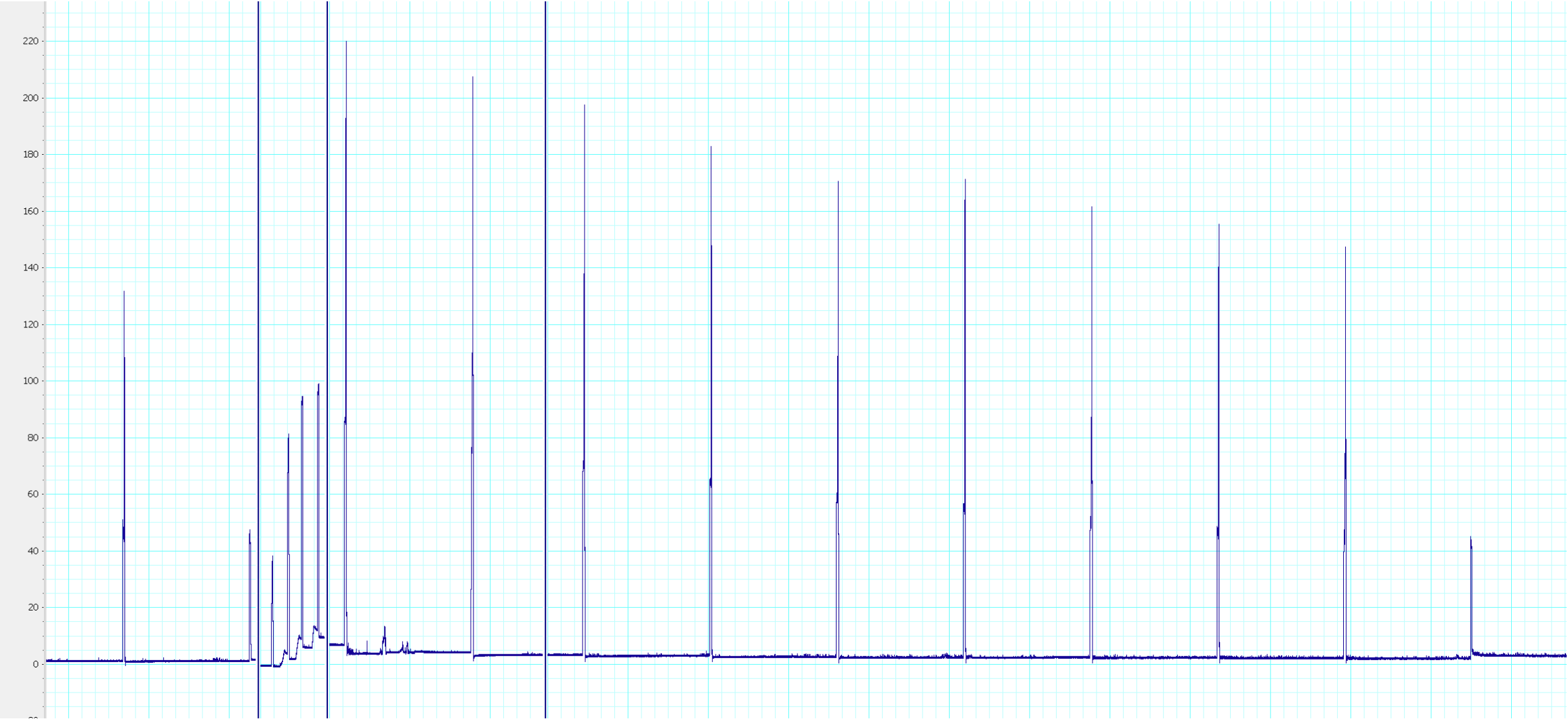

Supplement: S3 Fig — (PDF) [file pone.0254274.s003.pdf]

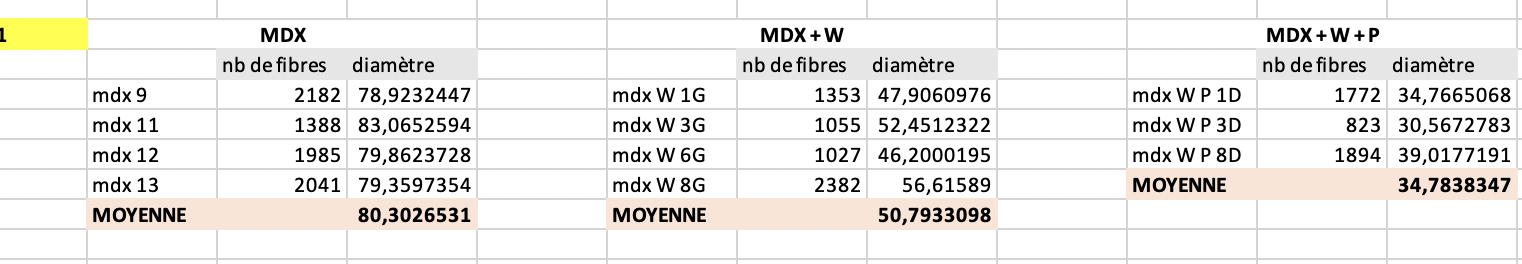

Supplement: S4 Table — (PNG) [file pone.0254274.s009.png]

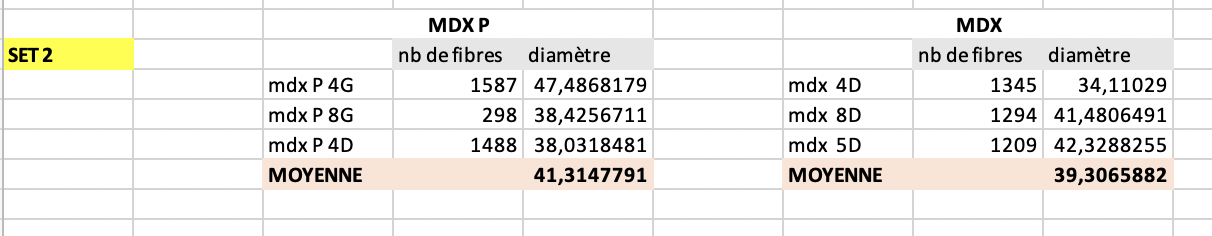

Supplement: S5 Table — (PNG) [file pone.0254274.s010.png]
