# Supplementary material for: The beneficial effect of chronic muscular exercise on muscle fragility is increased by Prox1 gene transfer in dystrophic mdx muscle
Source: PLoS One. 2022 Apr 18;17(4):e0254274. doi: 10.1371/journal.pone.0254274 (PMC9015141; doi:10.1371/journal.pone.0254274)
Supplement: S2 Fig — (PDF) [file pone.0254274.s002.pdf]

Mdx + W +P

TA 1D

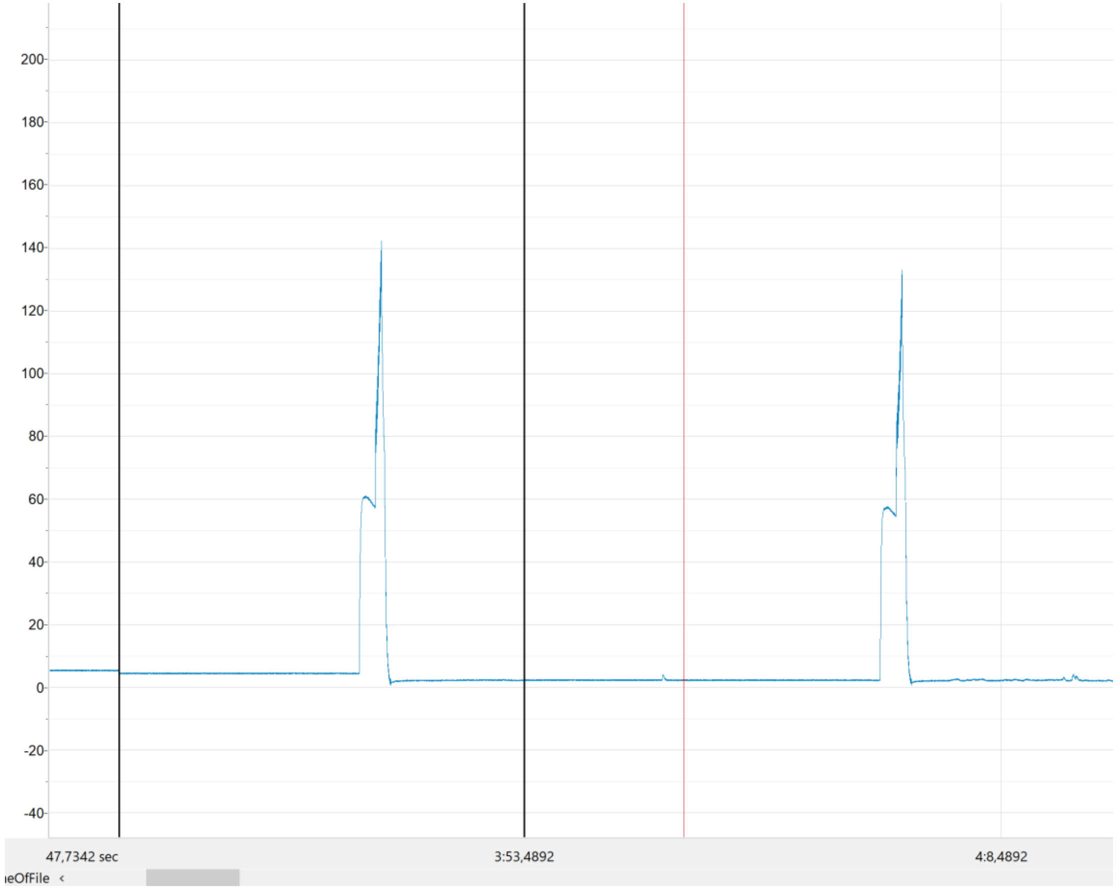

Firts lengthening contraction

PIC 1

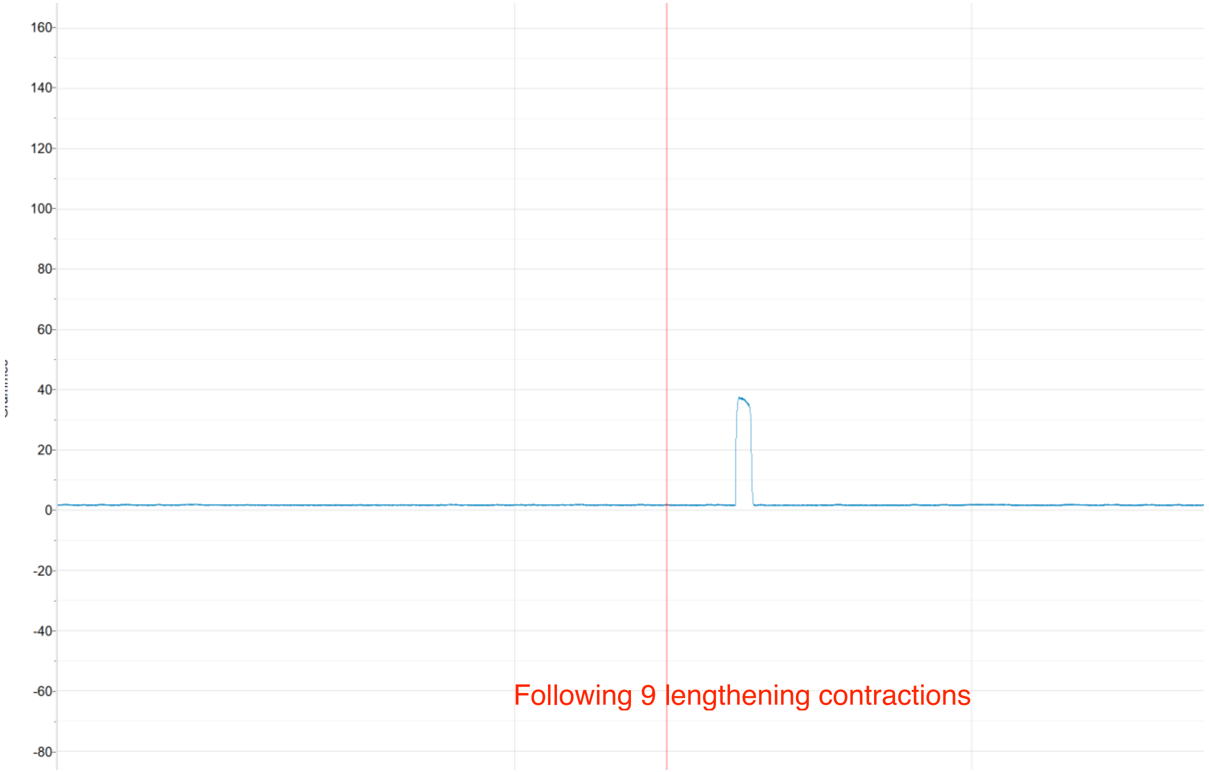

Following 9 lengthening contractions

PIC 10

Mdx + W +P

TA 3D

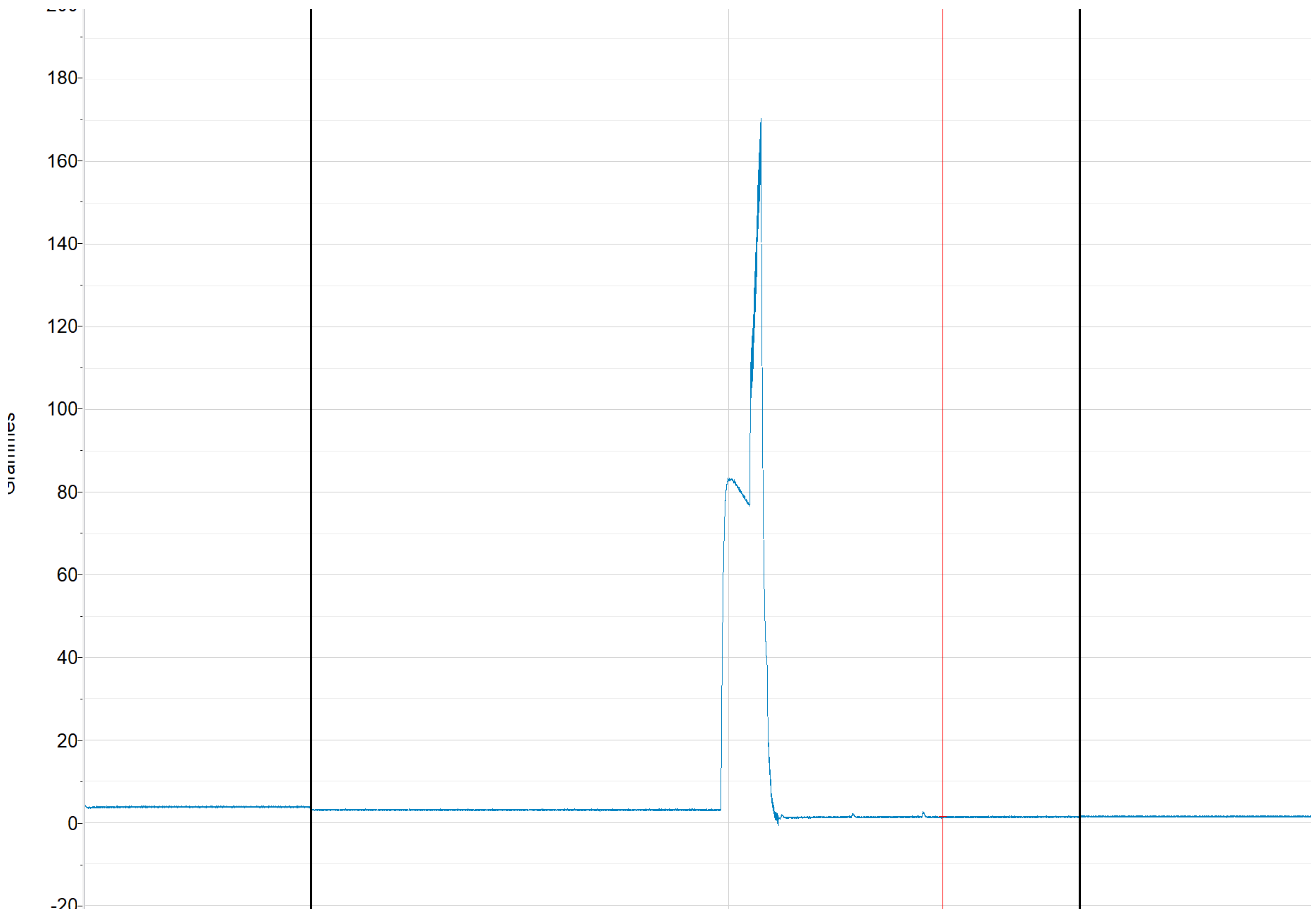

PIC 1

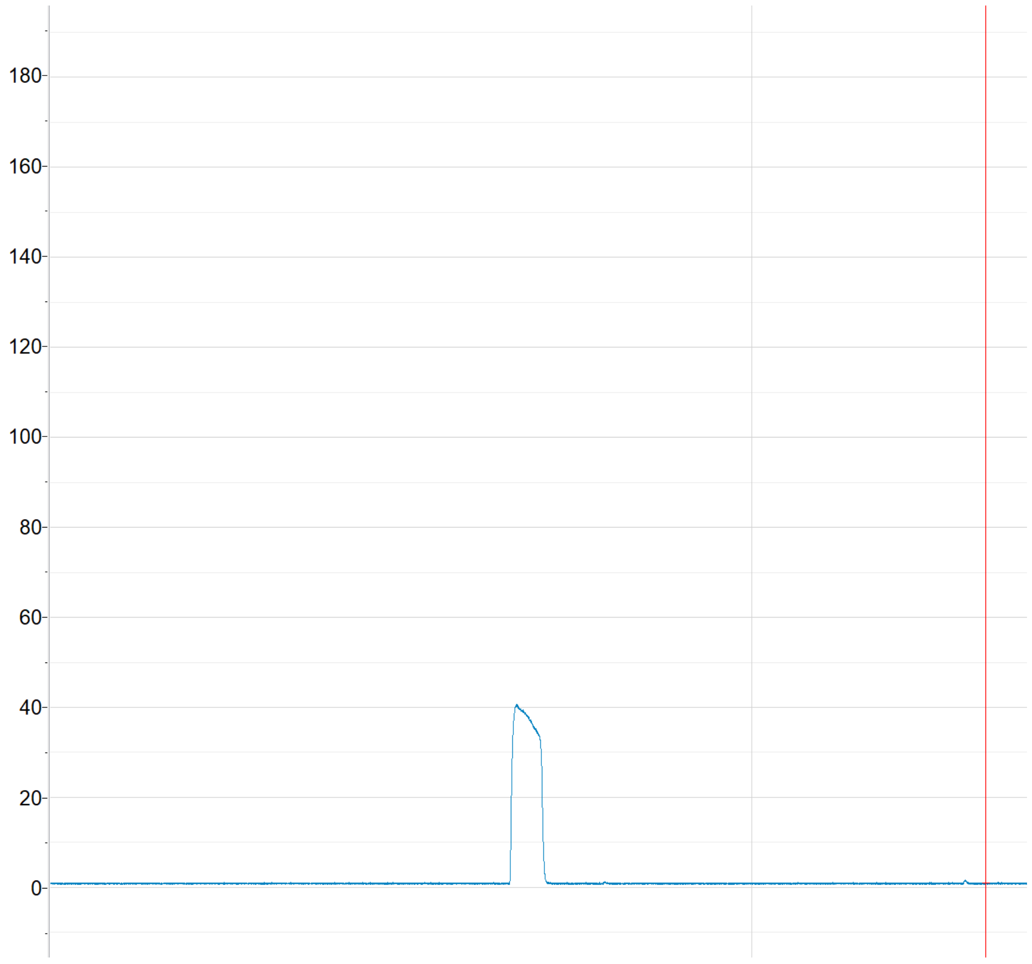

PIC 10

Mdx + W +P

TA 5D

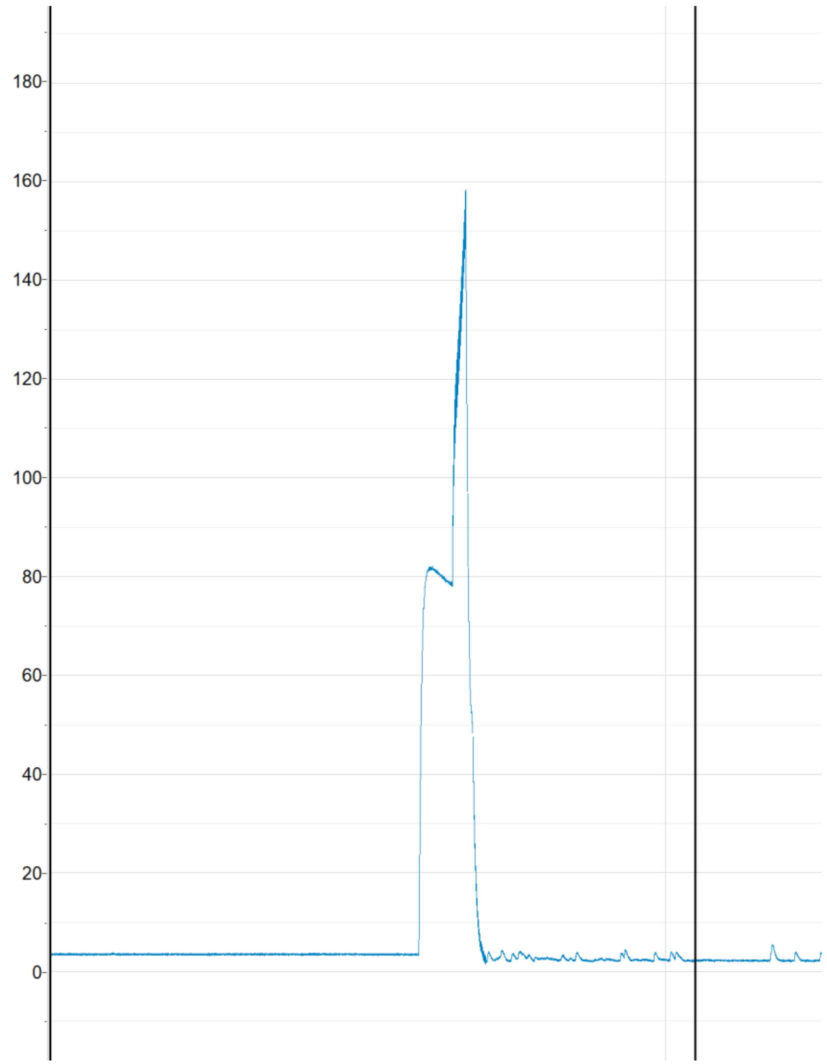

PIC 1

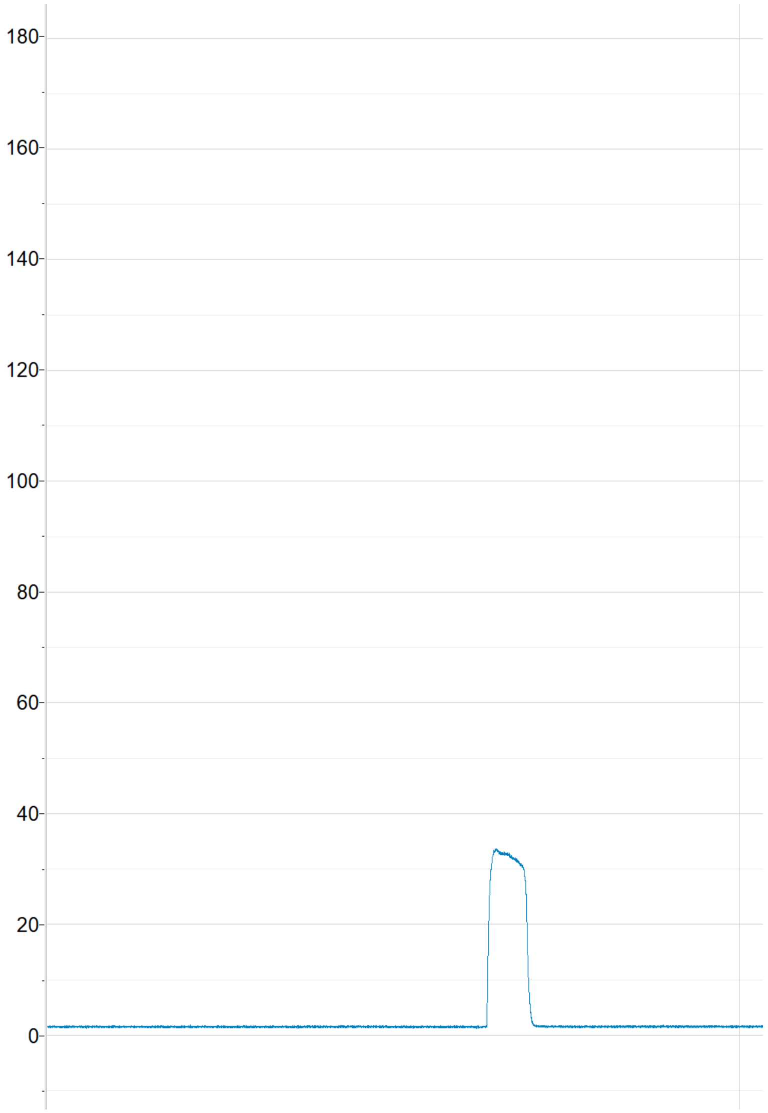

PIC 10

Mdx + W +P

TA 6D

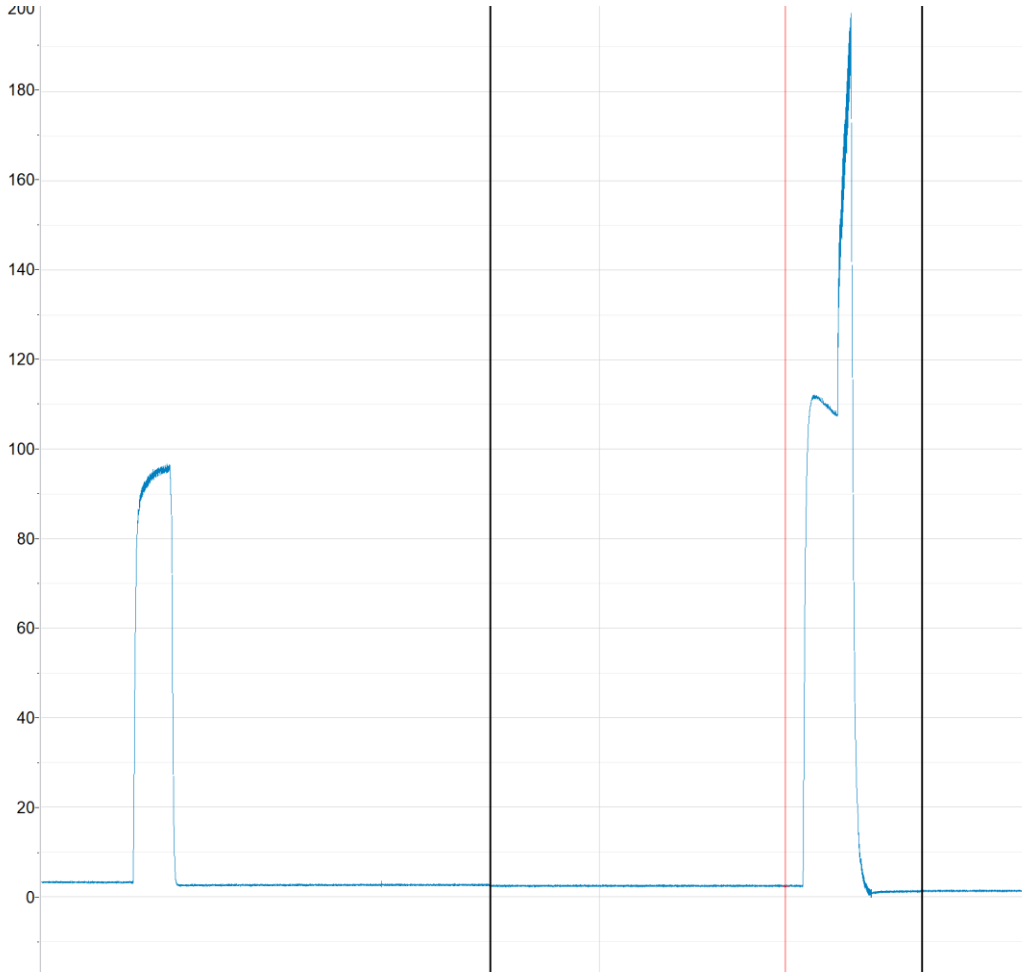

PIC 1

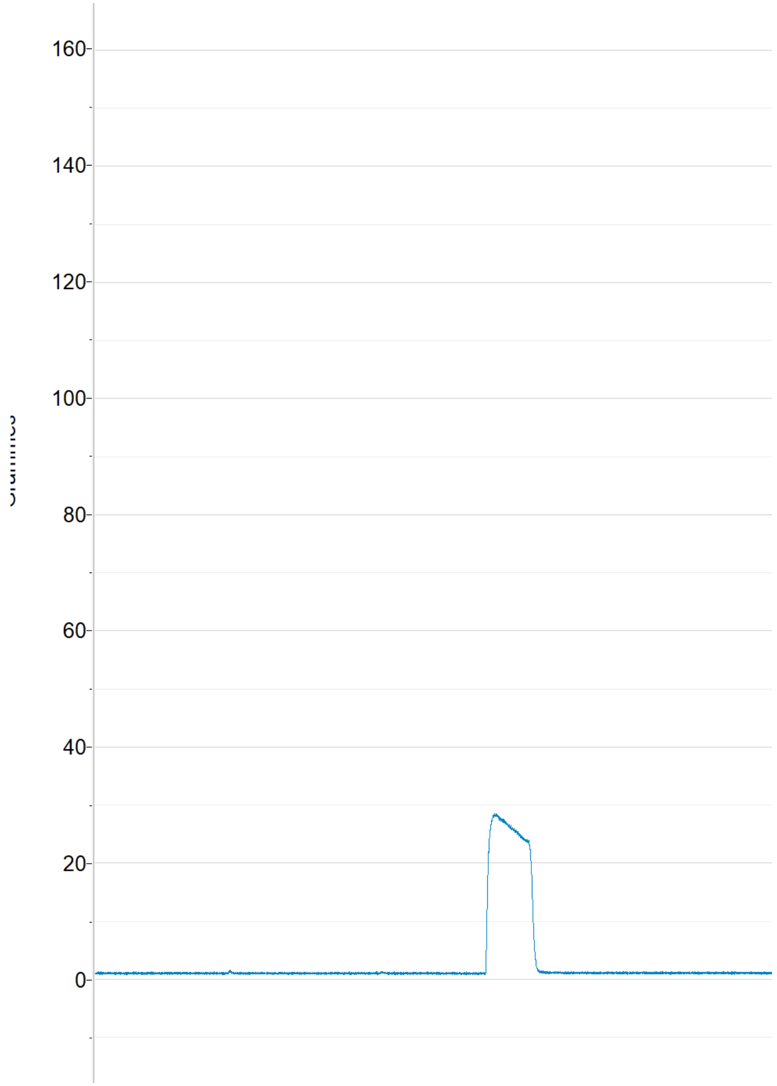

PIC 10

Mdx + W +P

TA 7D

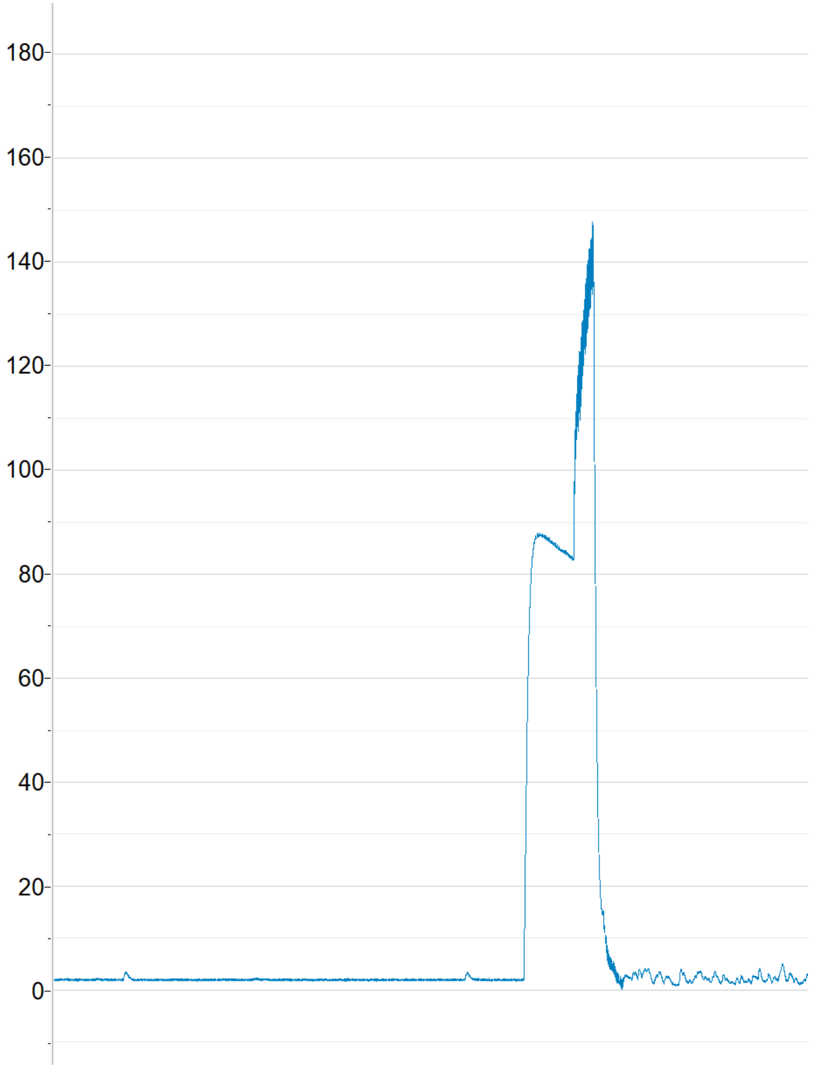

PIC 1

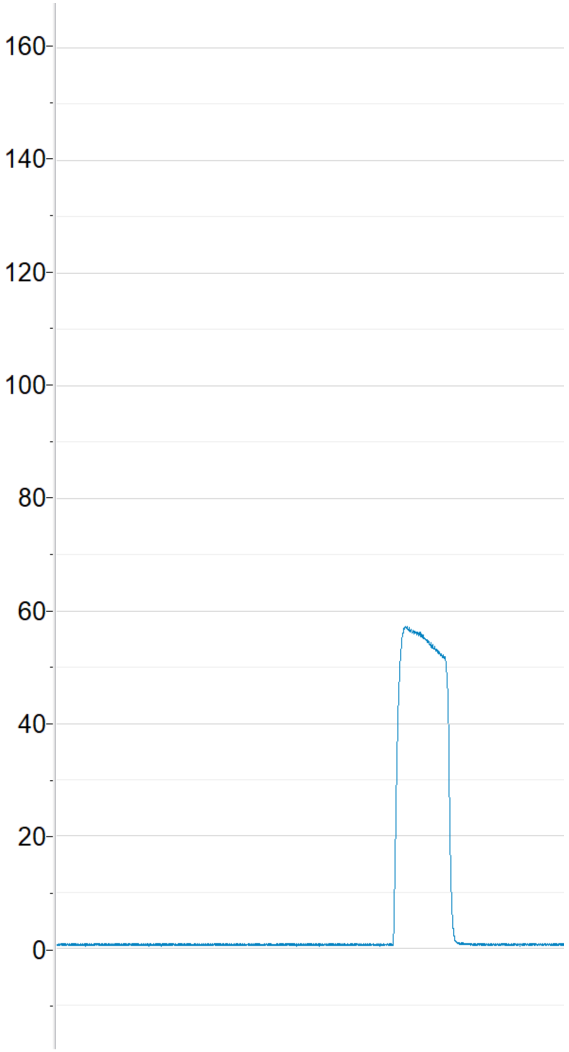

PIC 10

Mdx + W +P

TA 8D

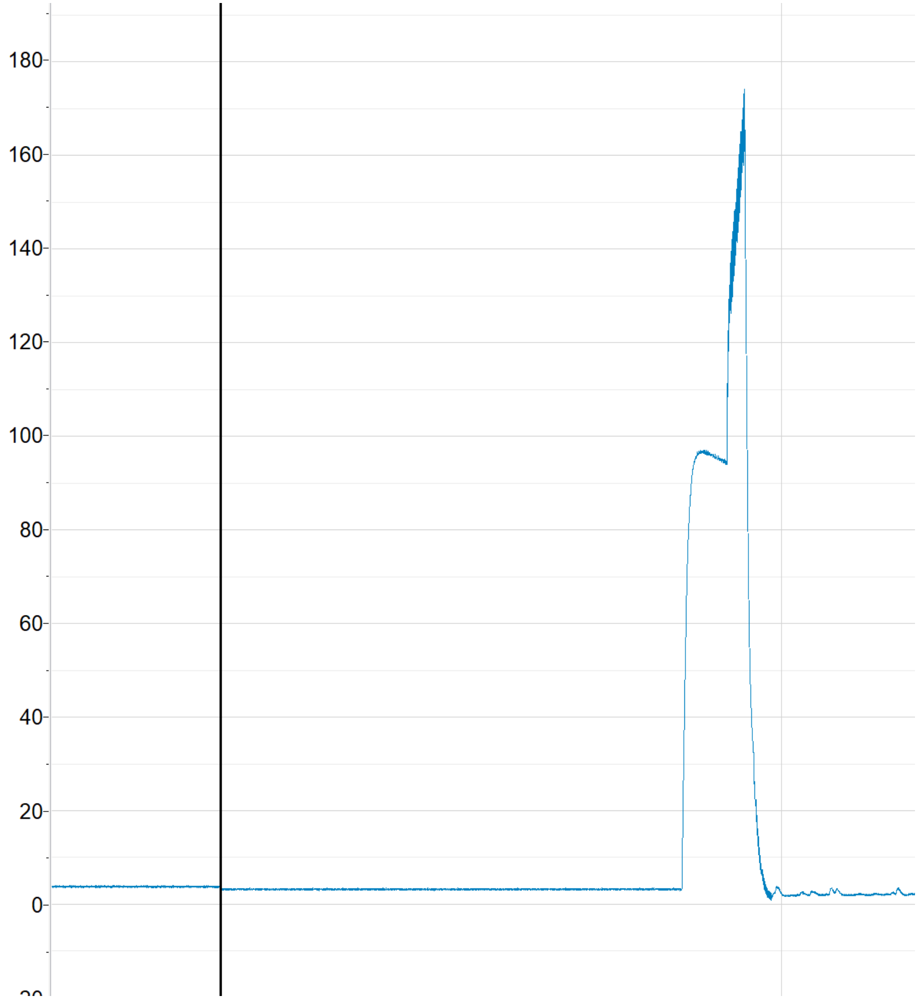

PIC 1

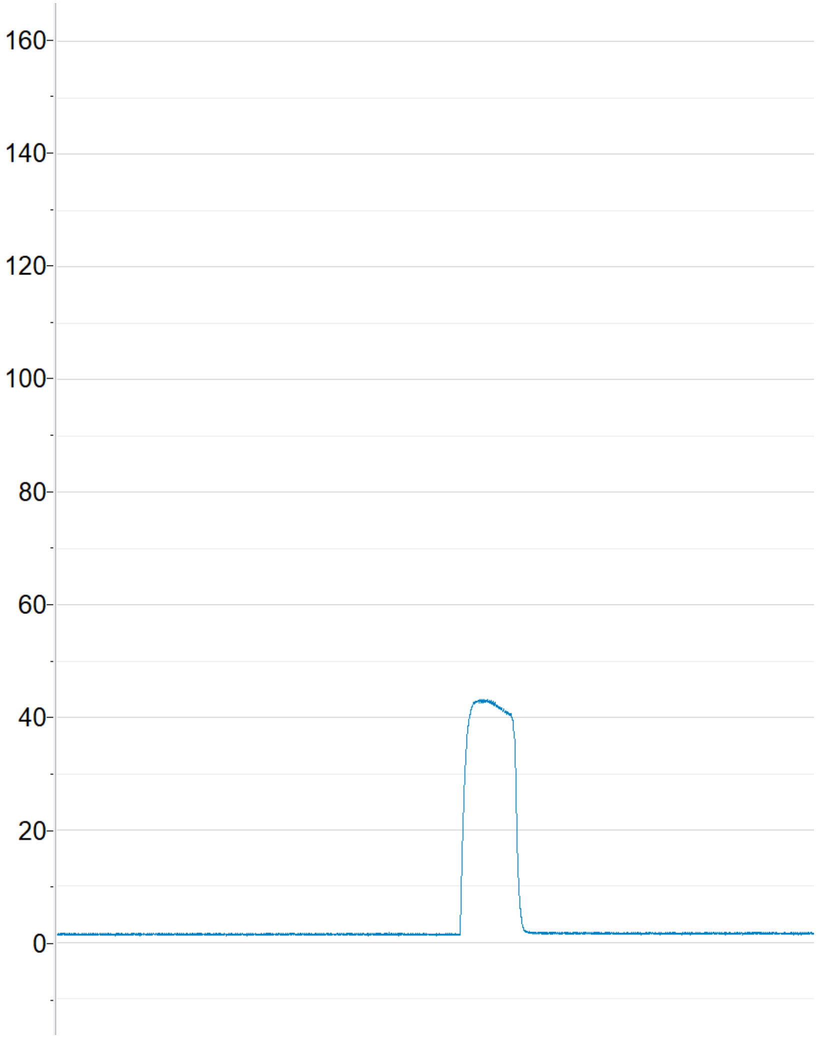

PIC 10

Mdx + W

TA 1G

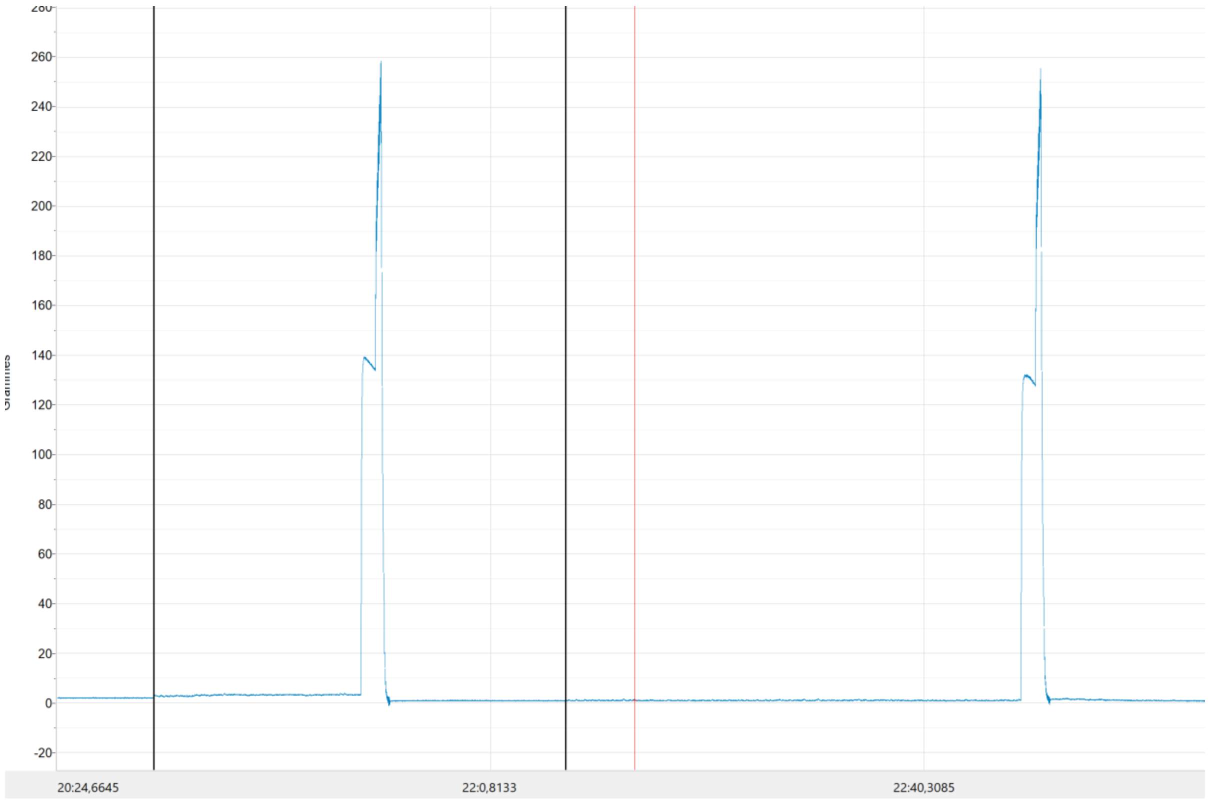

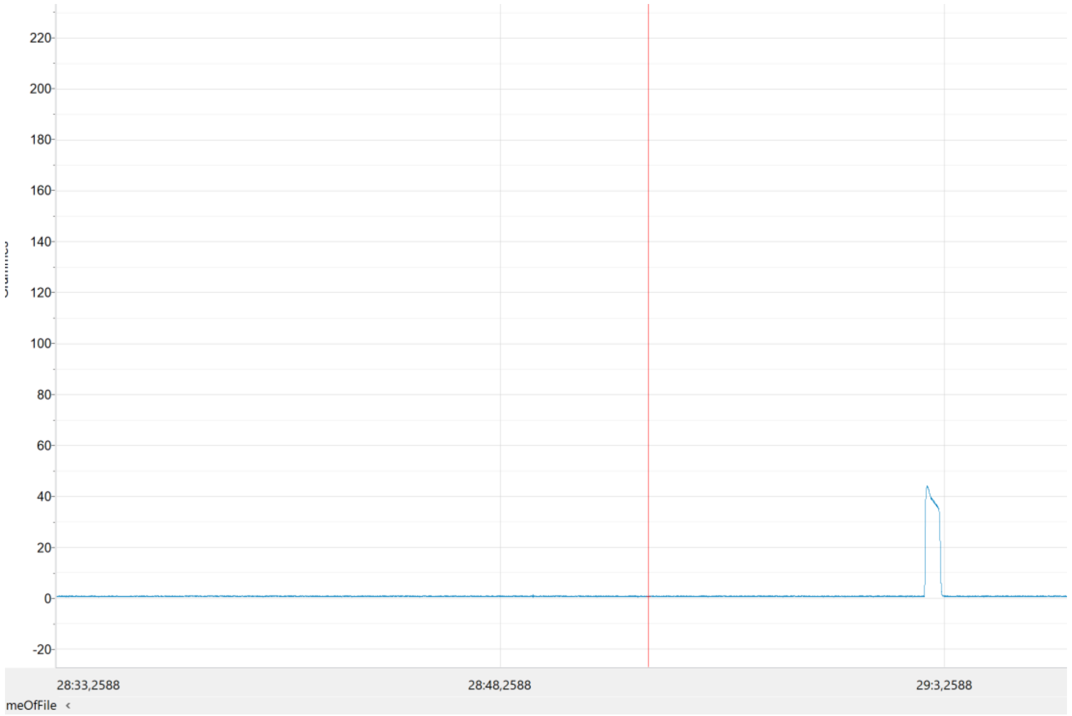

PIC 1

PIC 10

Mdx + W

TA 2D

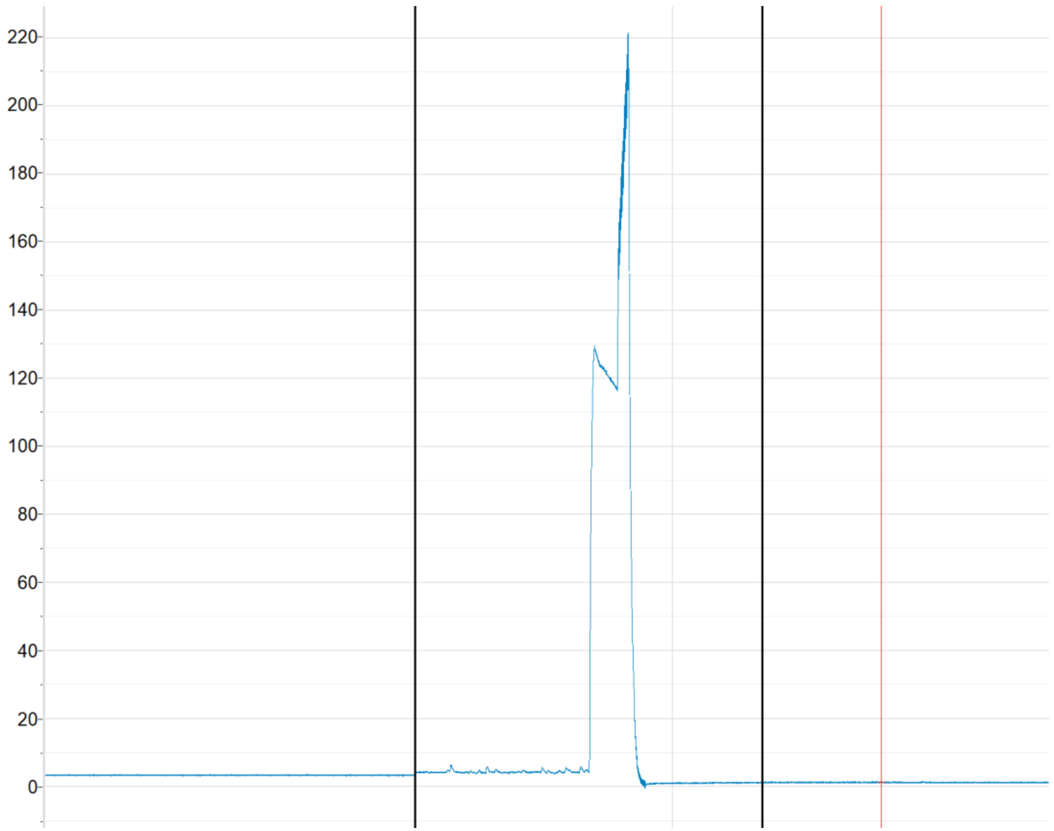

PIC 1

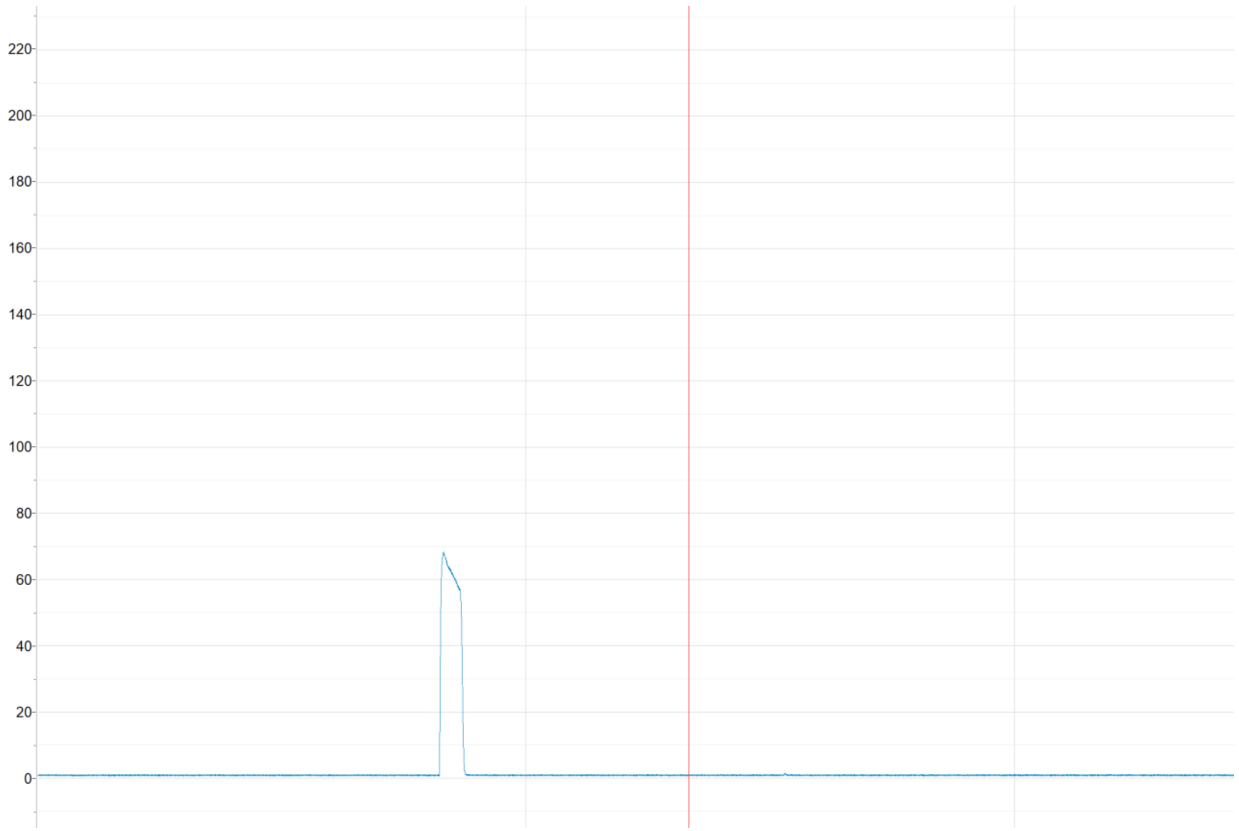

PIC 10

Mdx + W

TA 2G

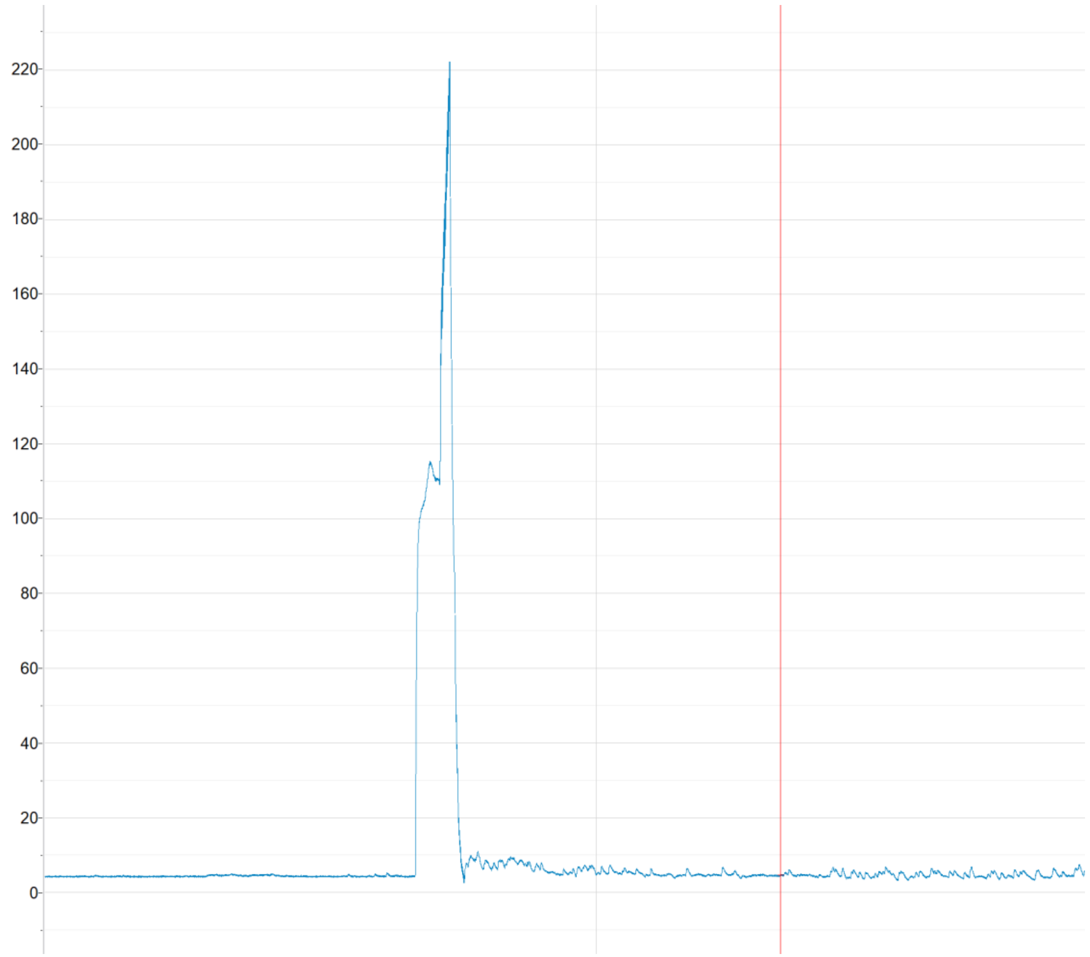

PIC 1

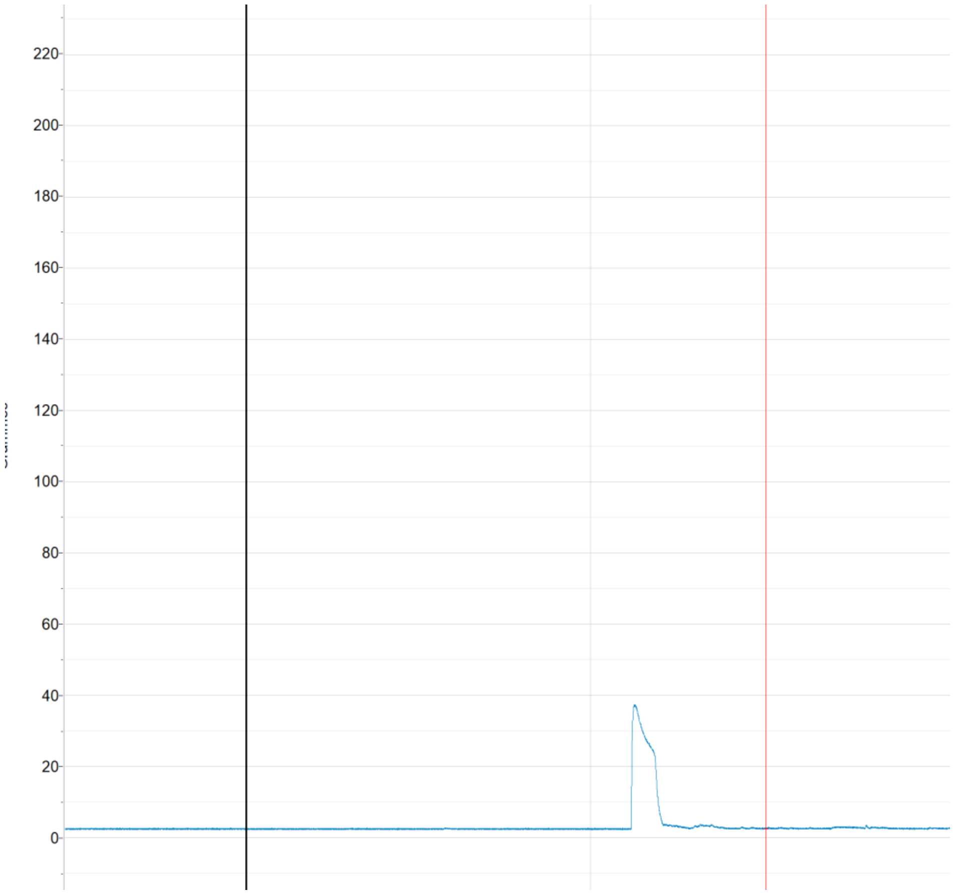

PIC 10

Mdx + W

TA 5G

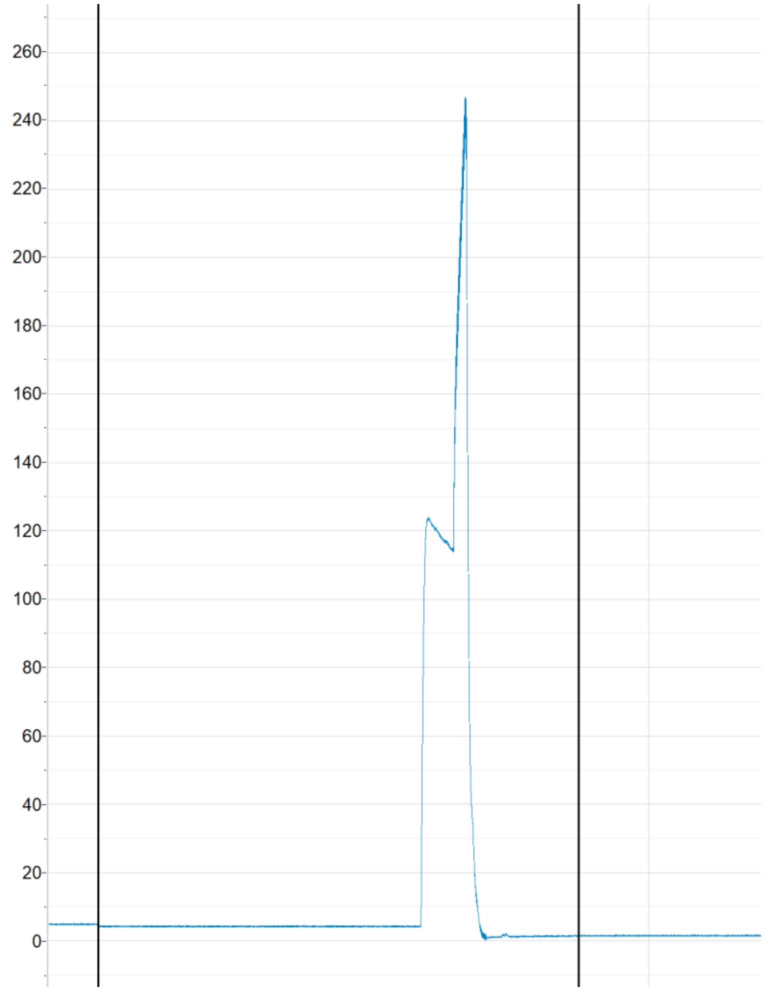

PIC 1

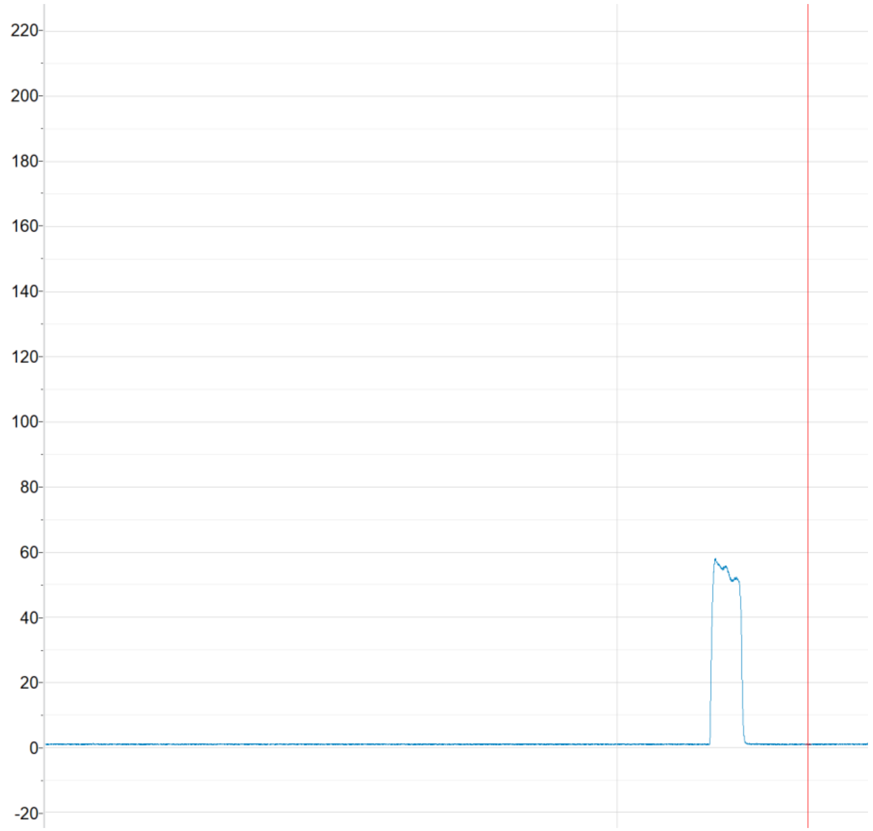

PIC 10

Mdx + W

TA 6G

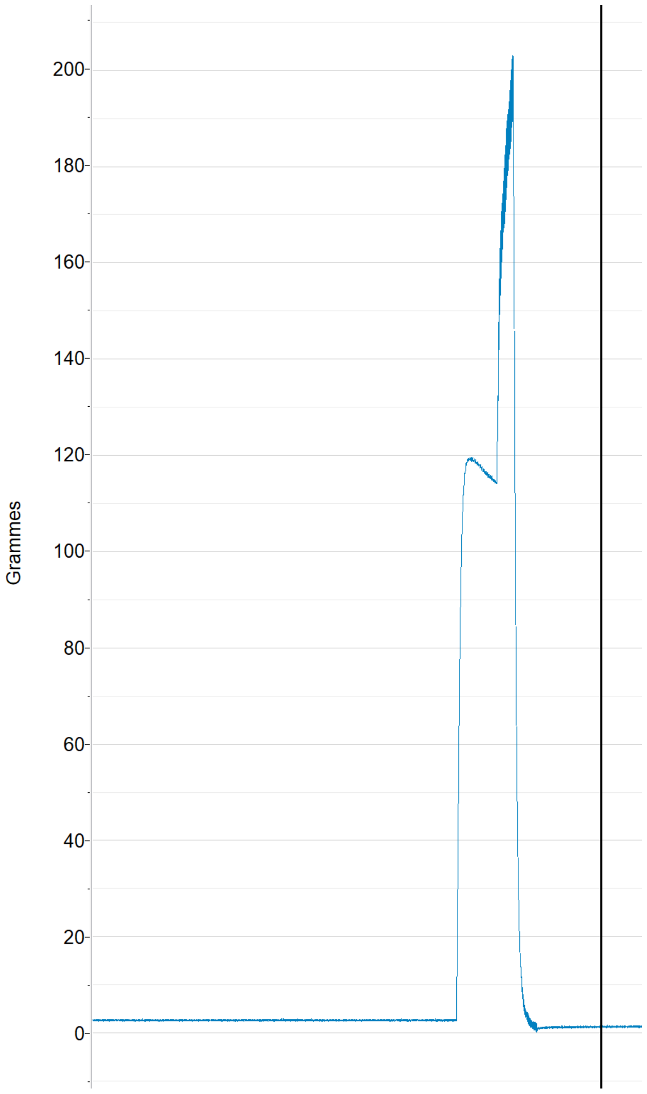

PIC 1

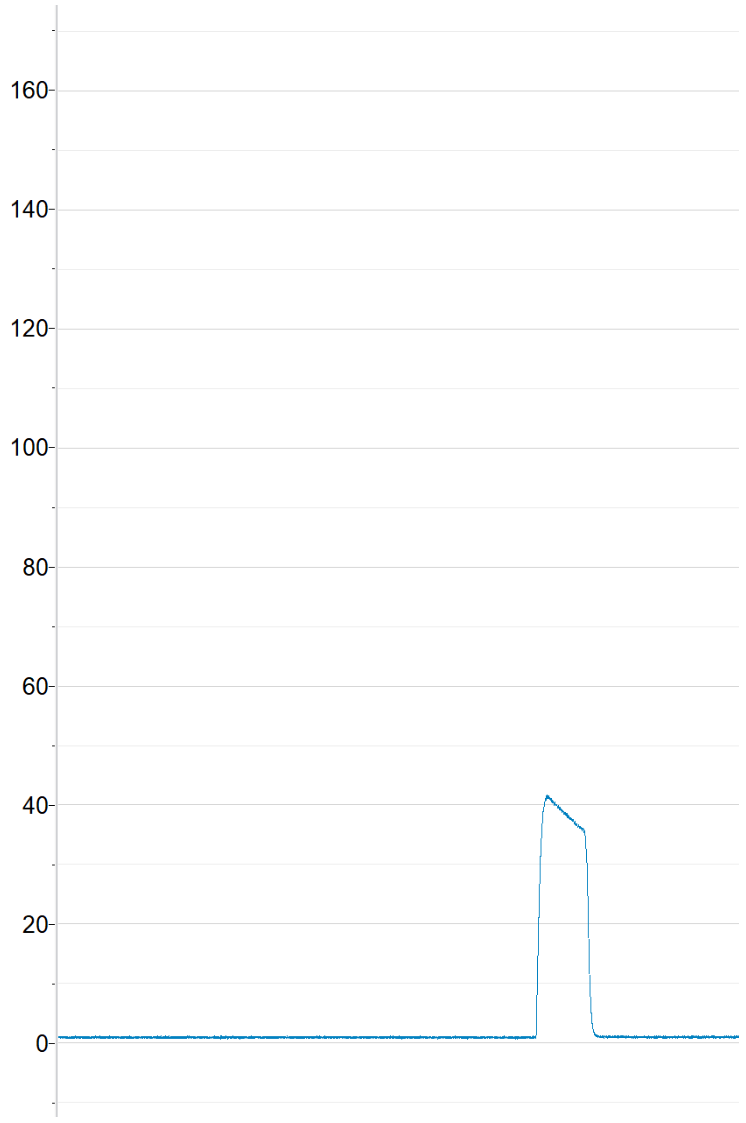

PIC 10

Mdx + W

TA 7G

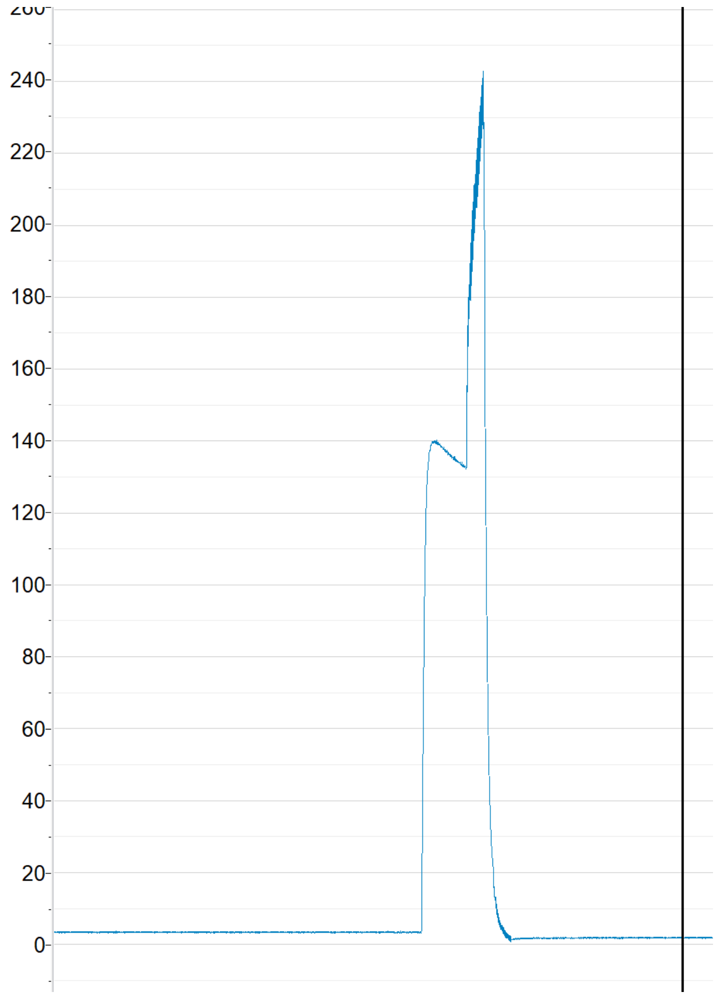

PIC 1

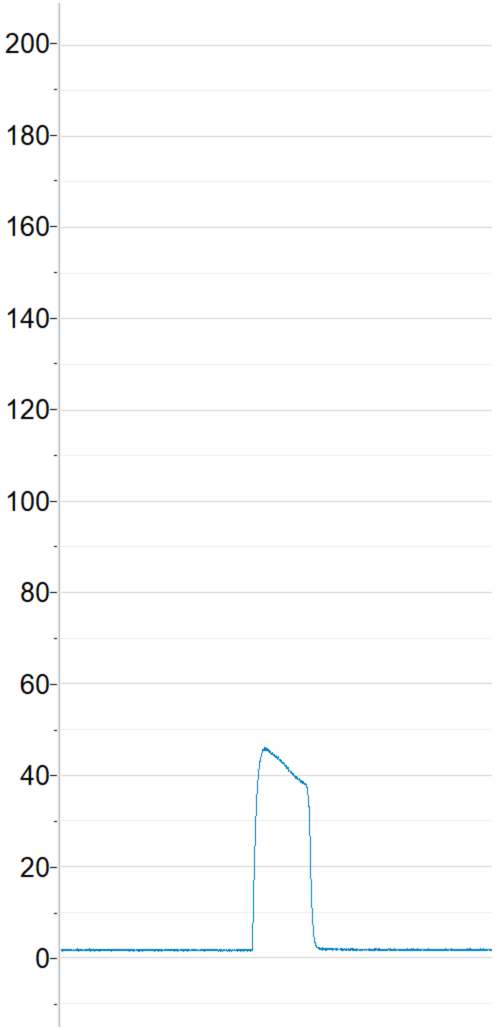

PIC 10

Mdx + W

TA 8G

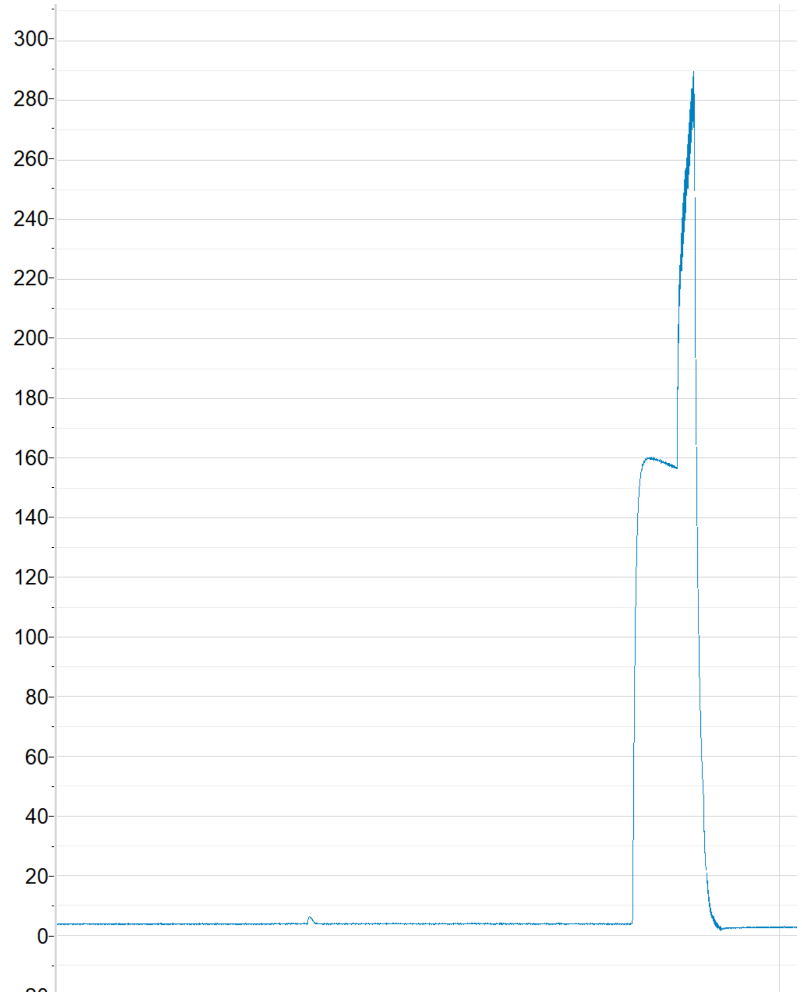

PIC 1

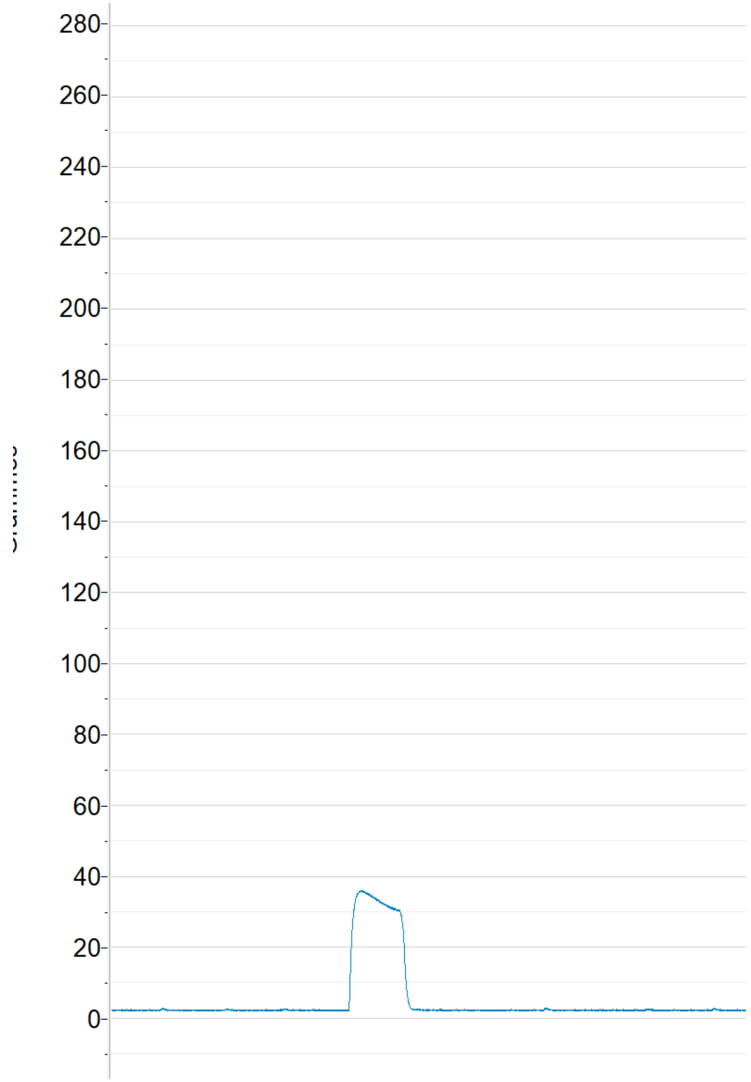

PIC 10

Mdx

TA 9D

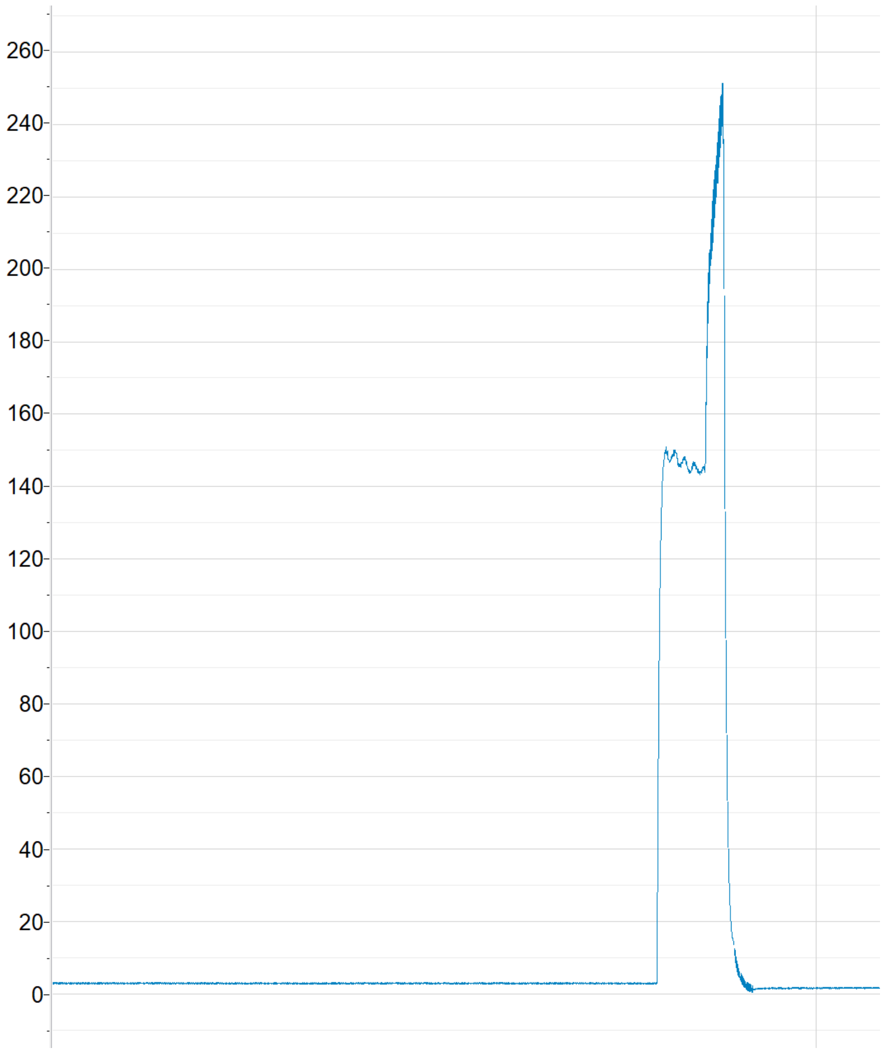

PIC 1

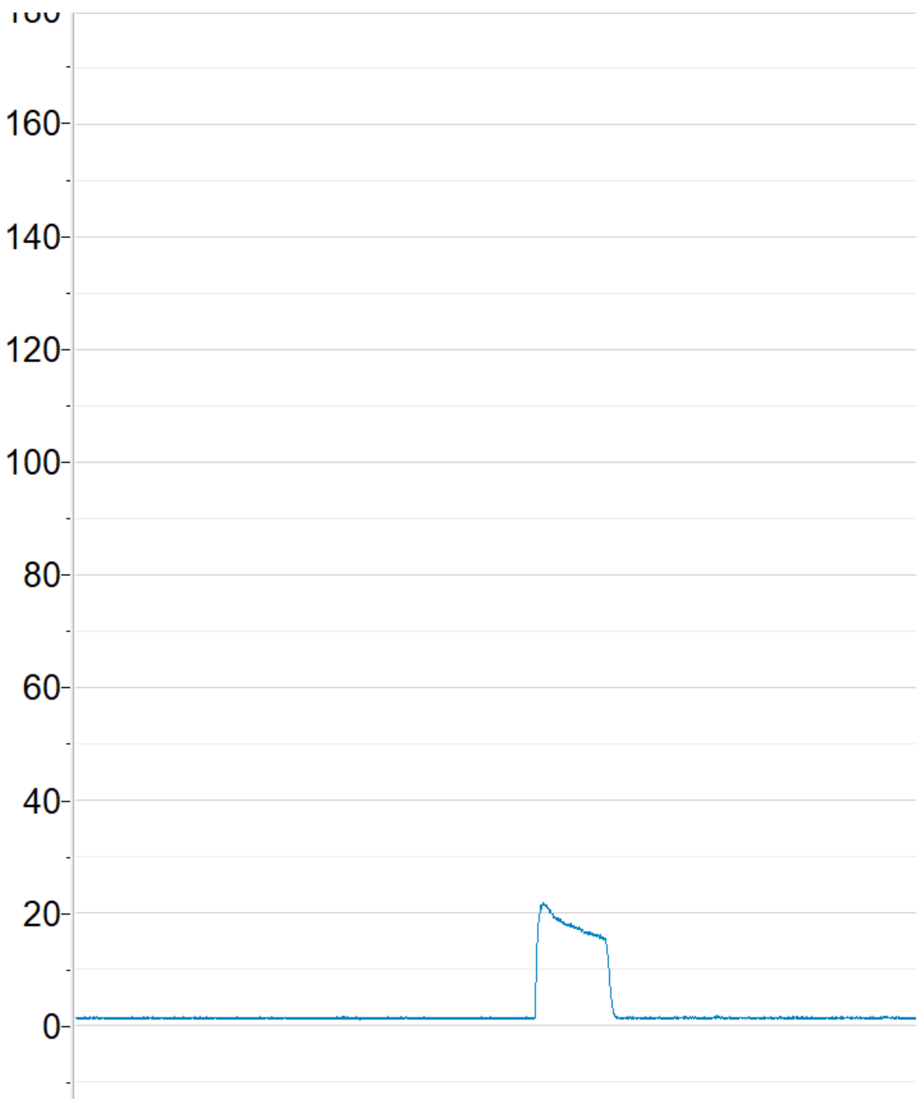

PIC 10

Mdx

TA 9G

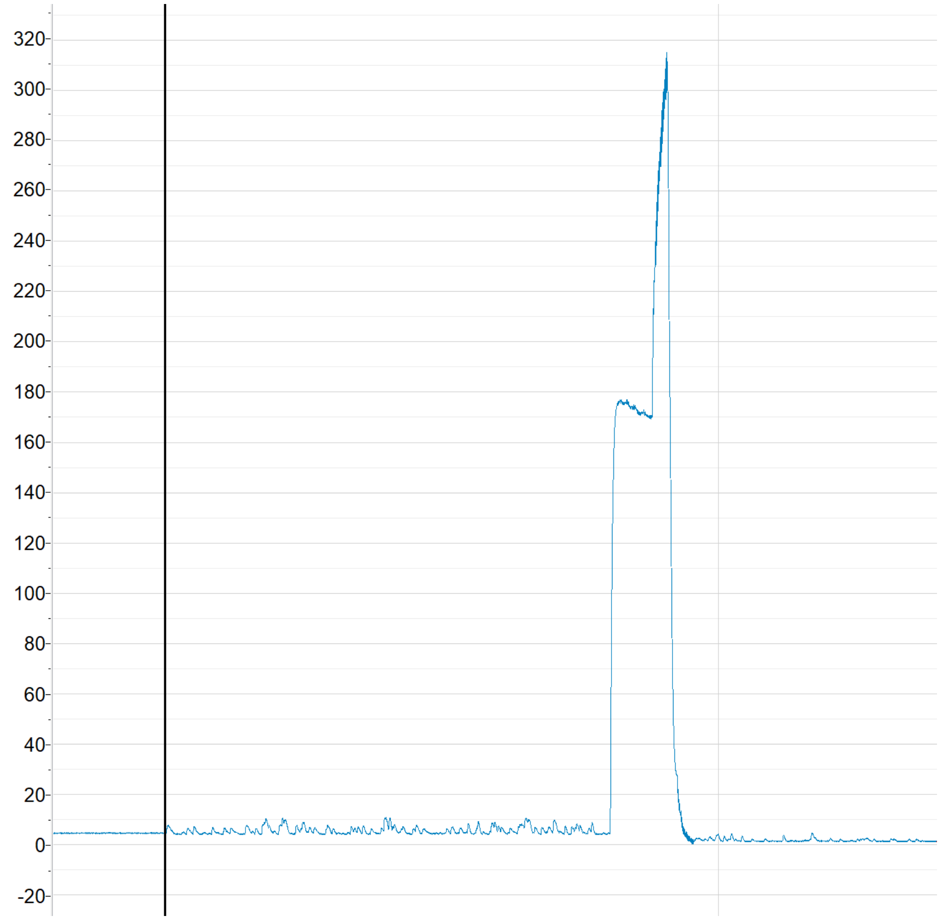

PIC 1

Mdx

TA 10D

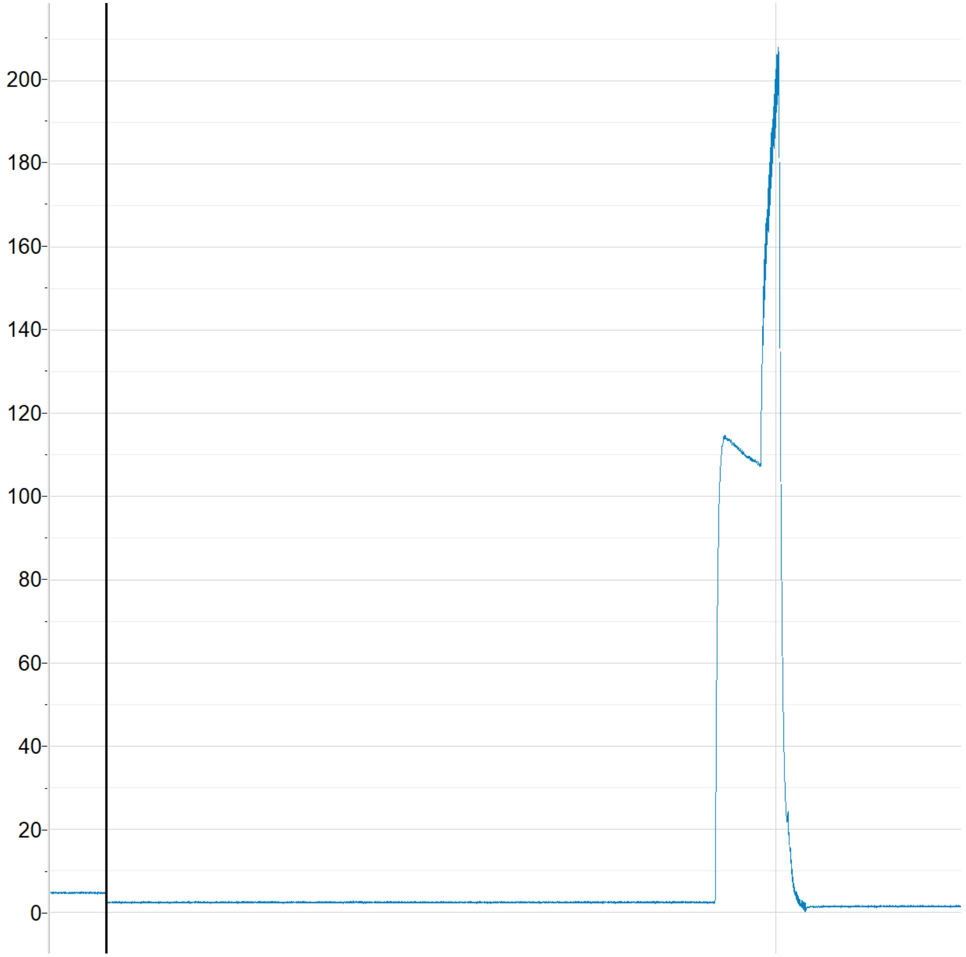

PIC 1

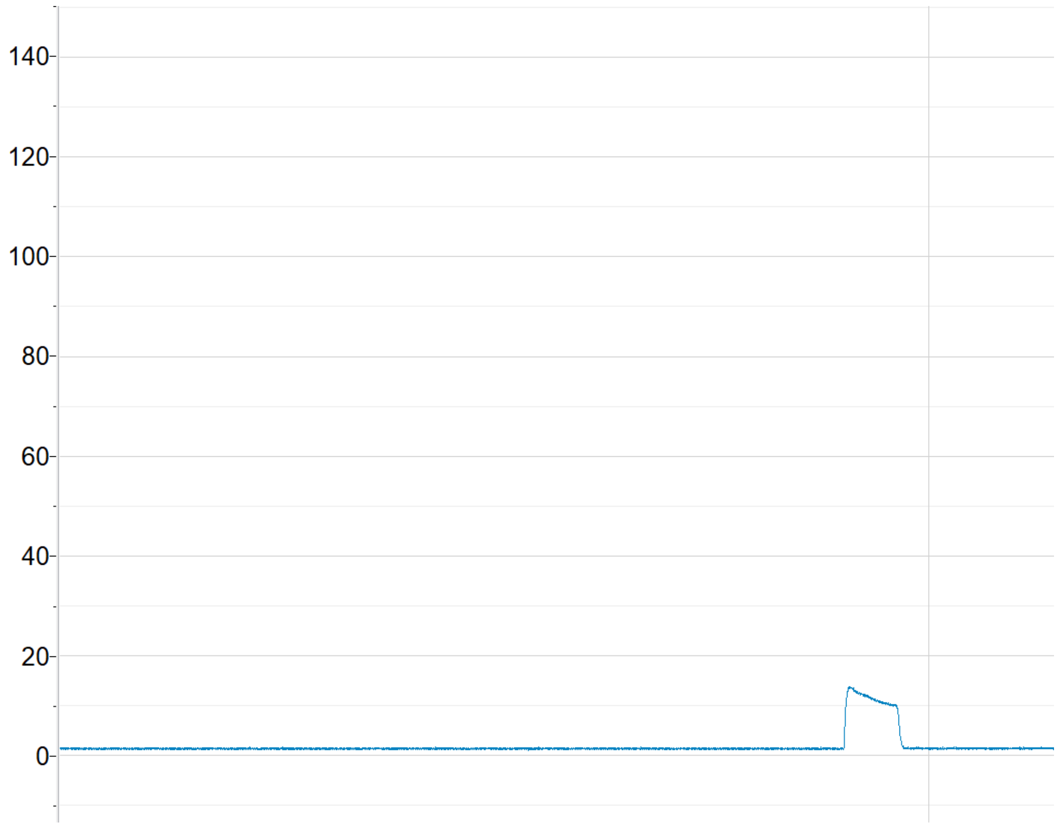

PIC 10

Mdx

TA 10G

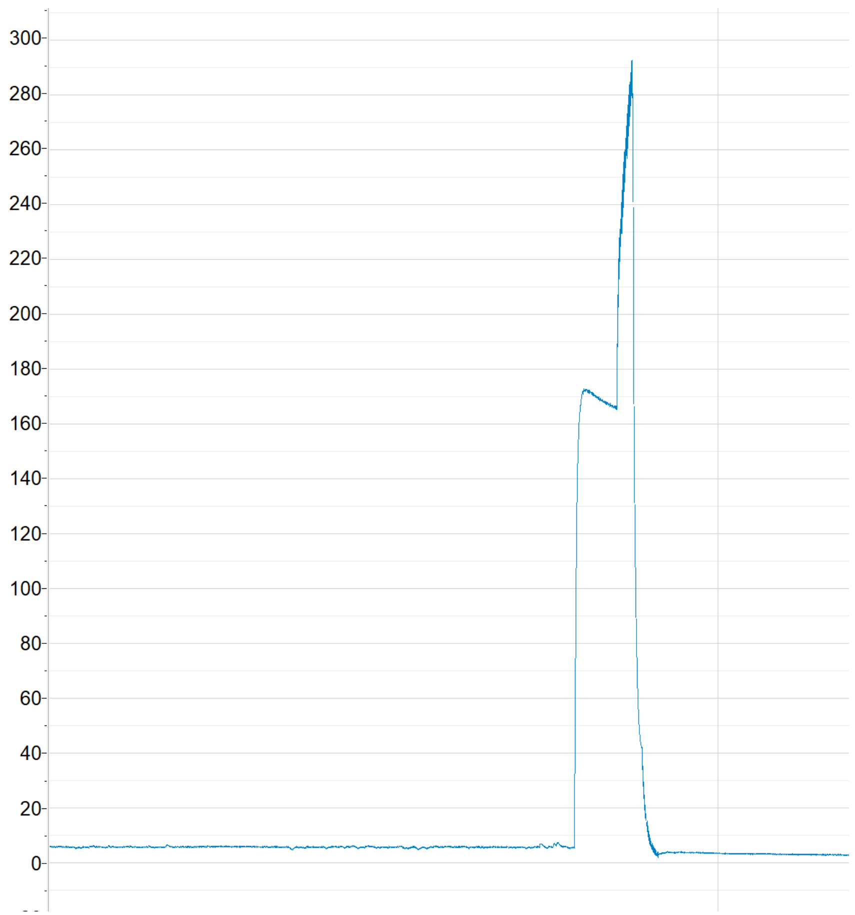

PIC 1

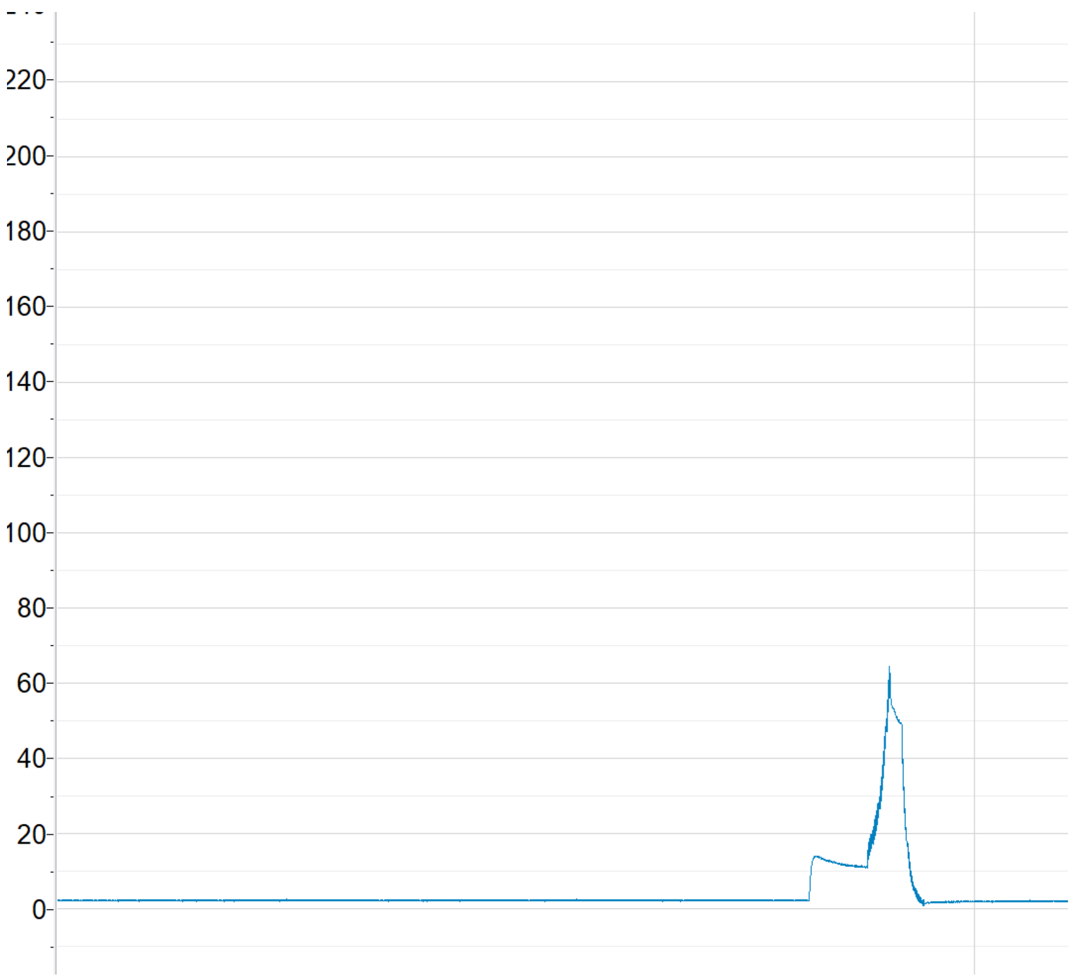

PIC 10

Mdx

TA 11D

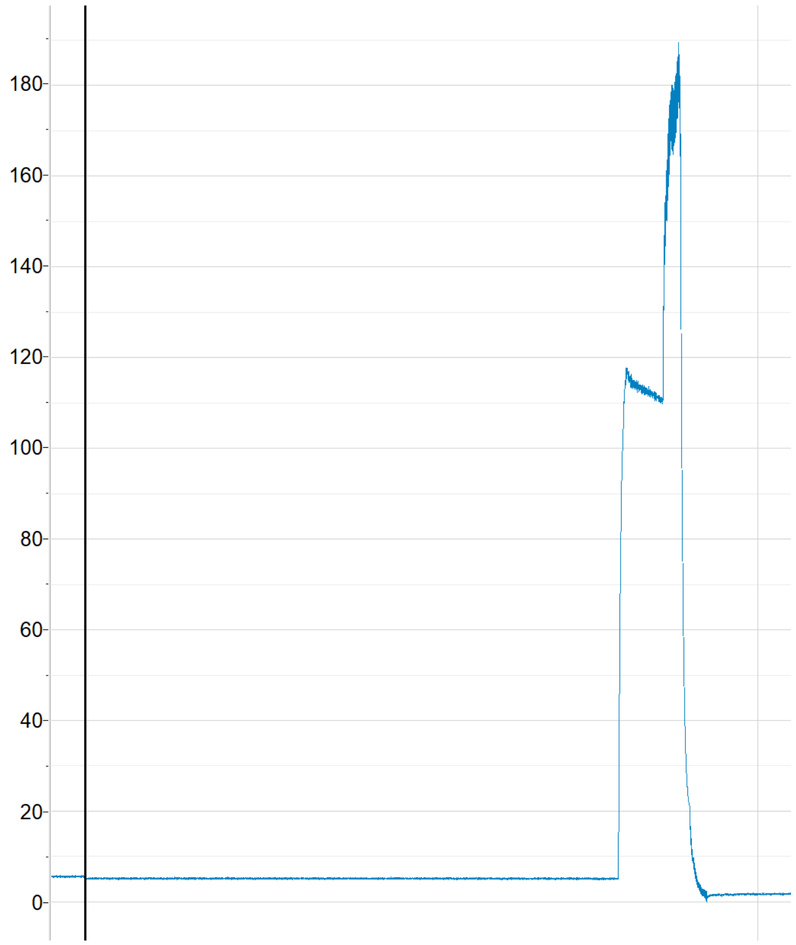

PIC 1

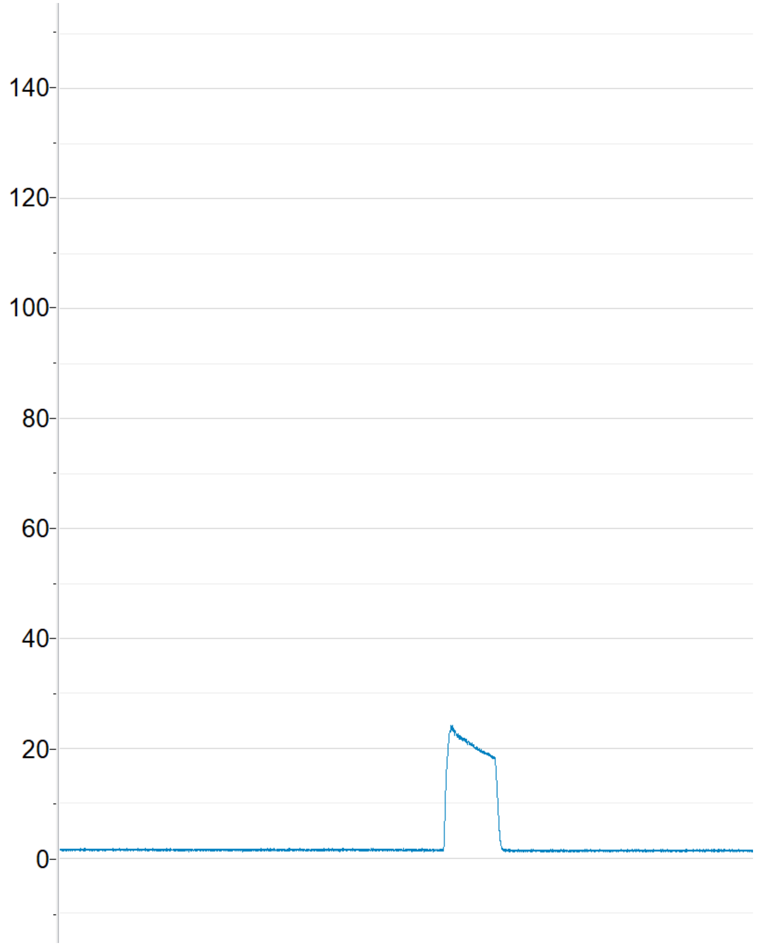

PIC 10

Mdx

TA 11G

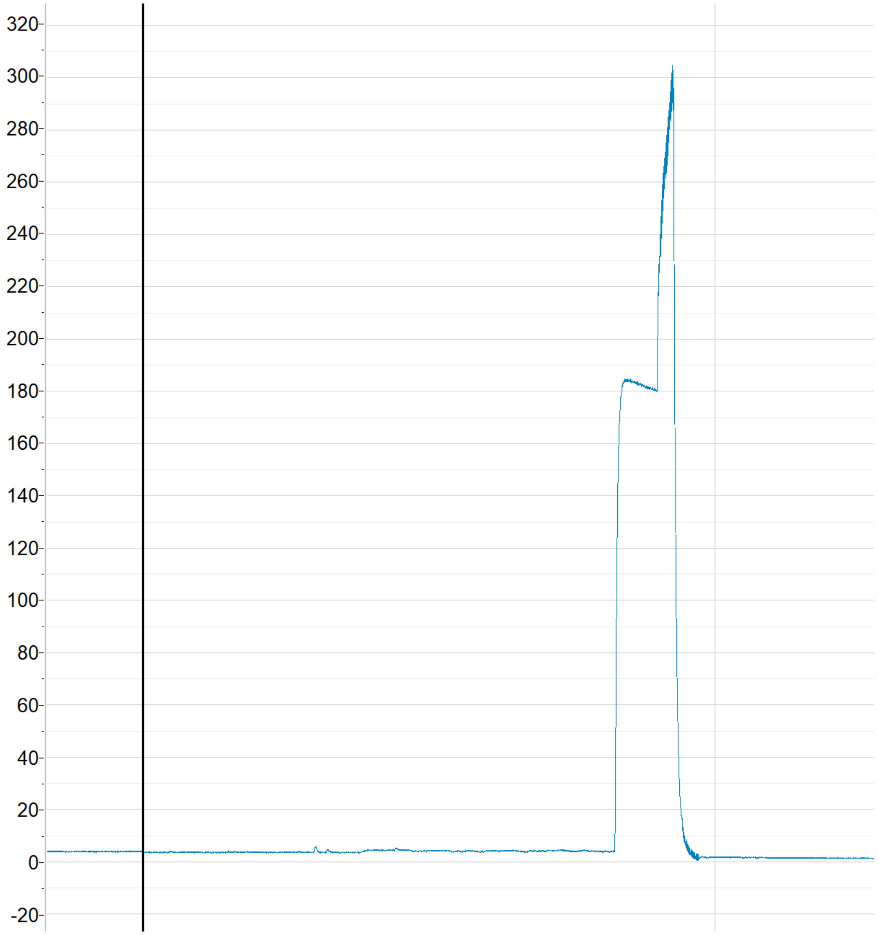

PIC 1

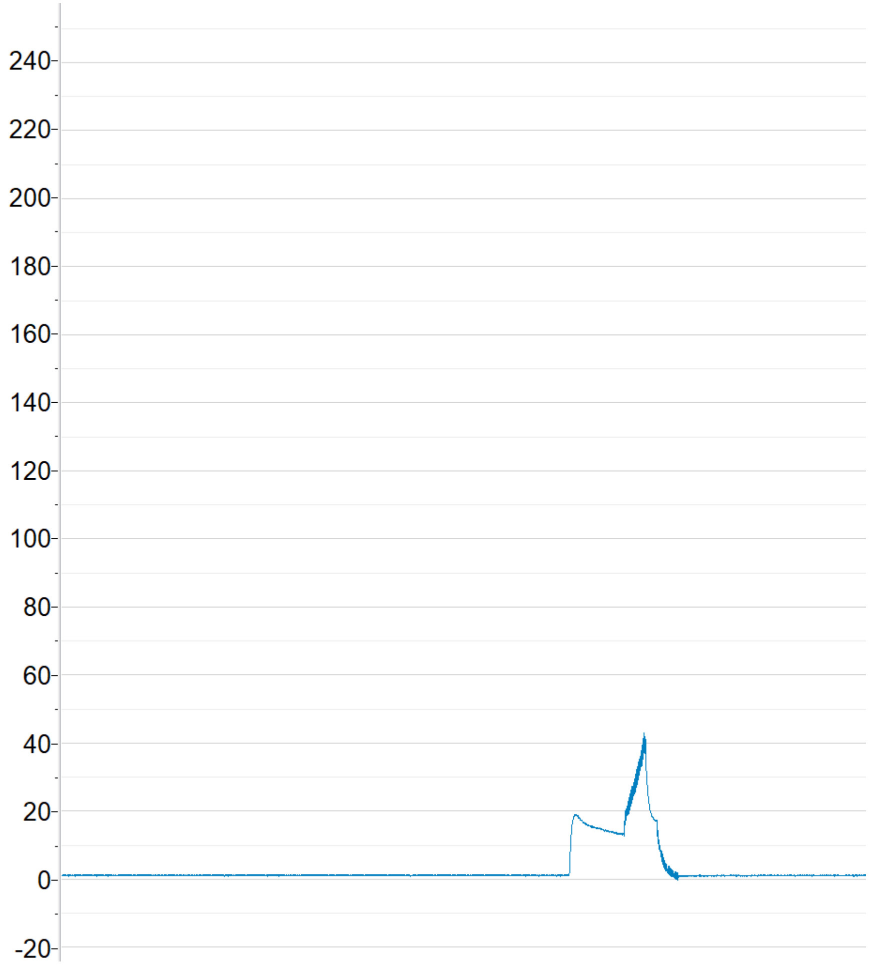

PIC 10

Mdx

TA 12D

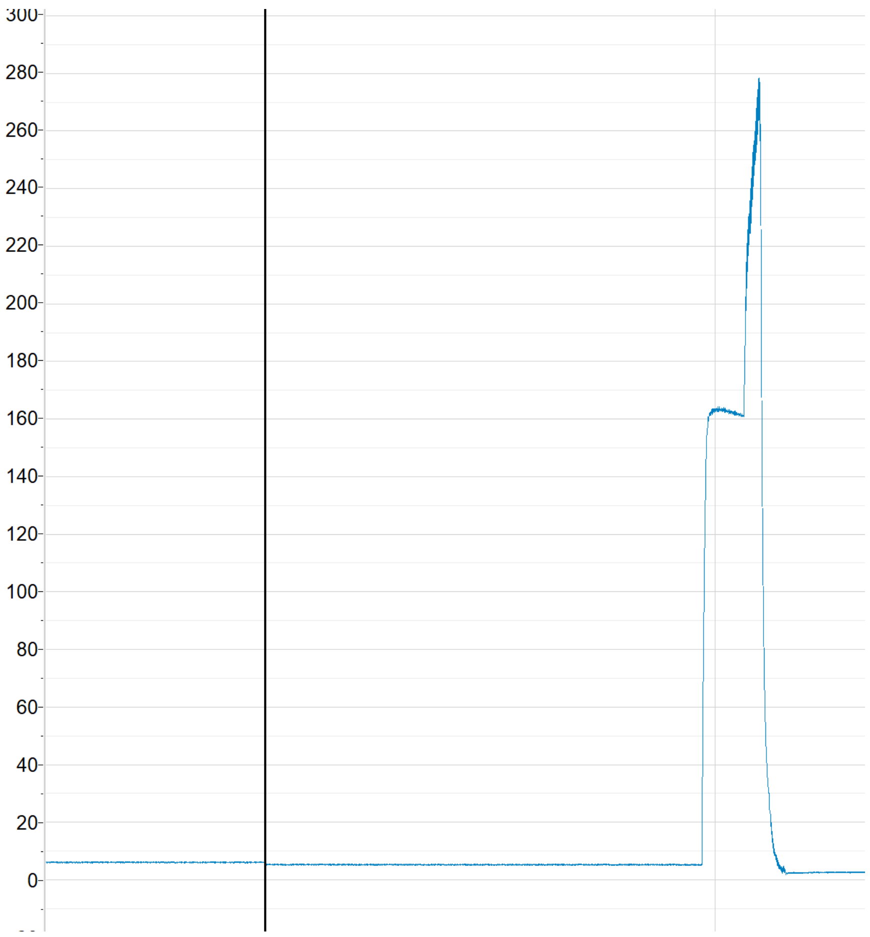

PIC 1

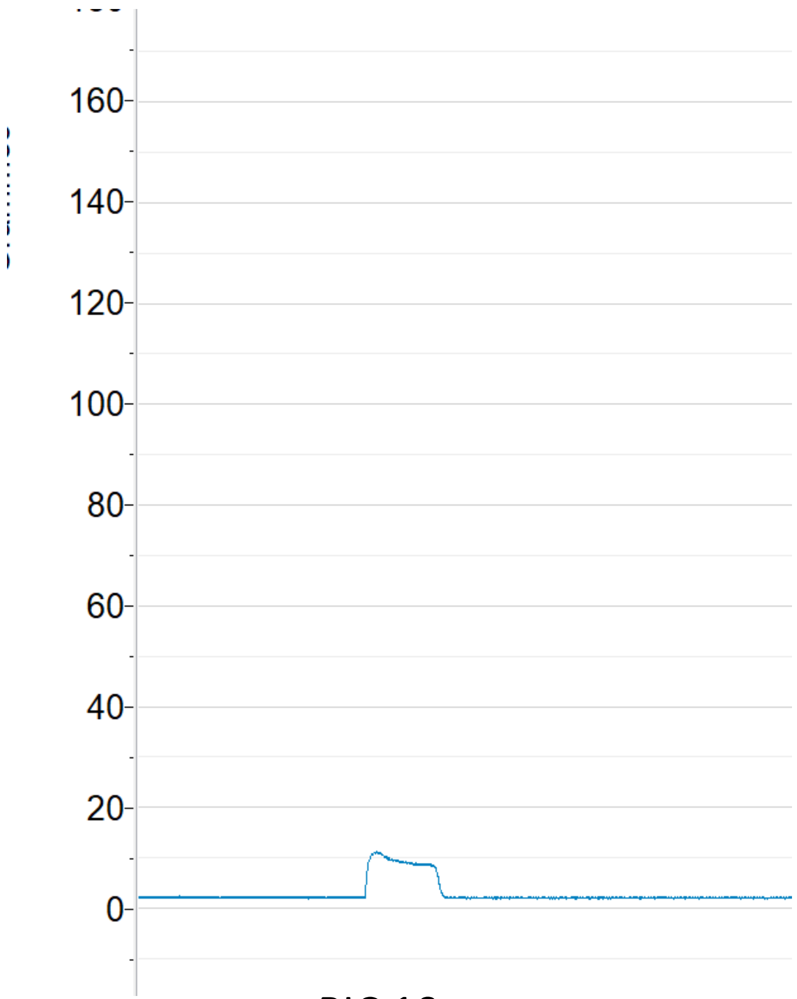

PIC 10

Mdx

TA 12G

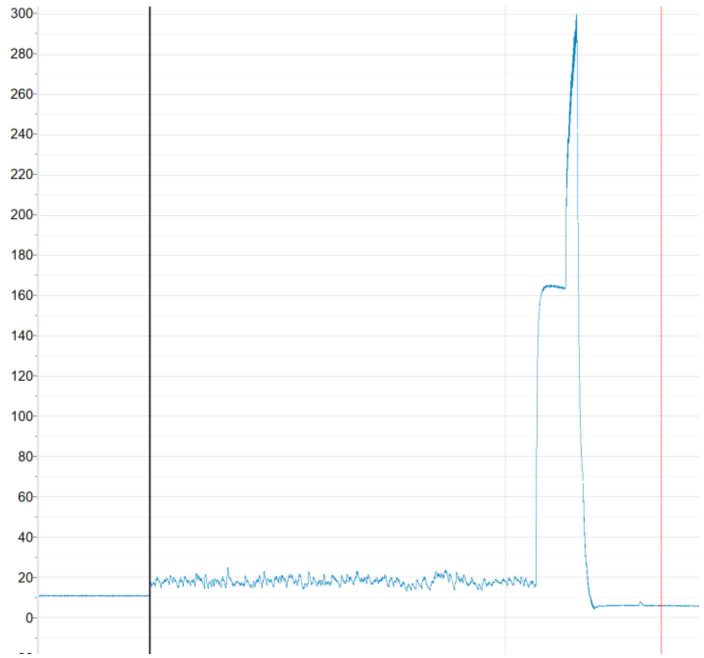

PIC 1

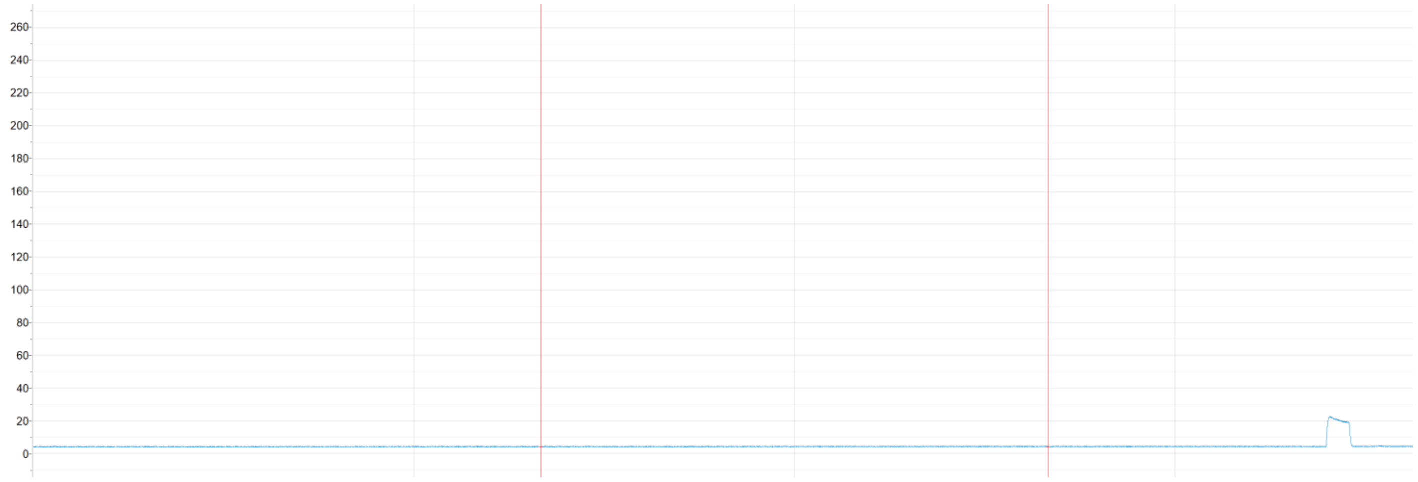

PIC 10
