# Supplementary material for: The beneficial effect of chronic muscular exercise on muscle fragility is increased by Prox1 gene transfer in dystrophic mdx muscle
Source: PLoS One. 2022 Apr 18;17(4):e0254274. doi: 10.1371/journal.pone.0254274 (PMC9015141; doi:10.1371/journal.pone.0254274)
Supplement: S4 Fig — (PDF) [file pone.0254274.s004.pdf]

Scale bar 500um (20X)

SET 1

MDX

mdx 9

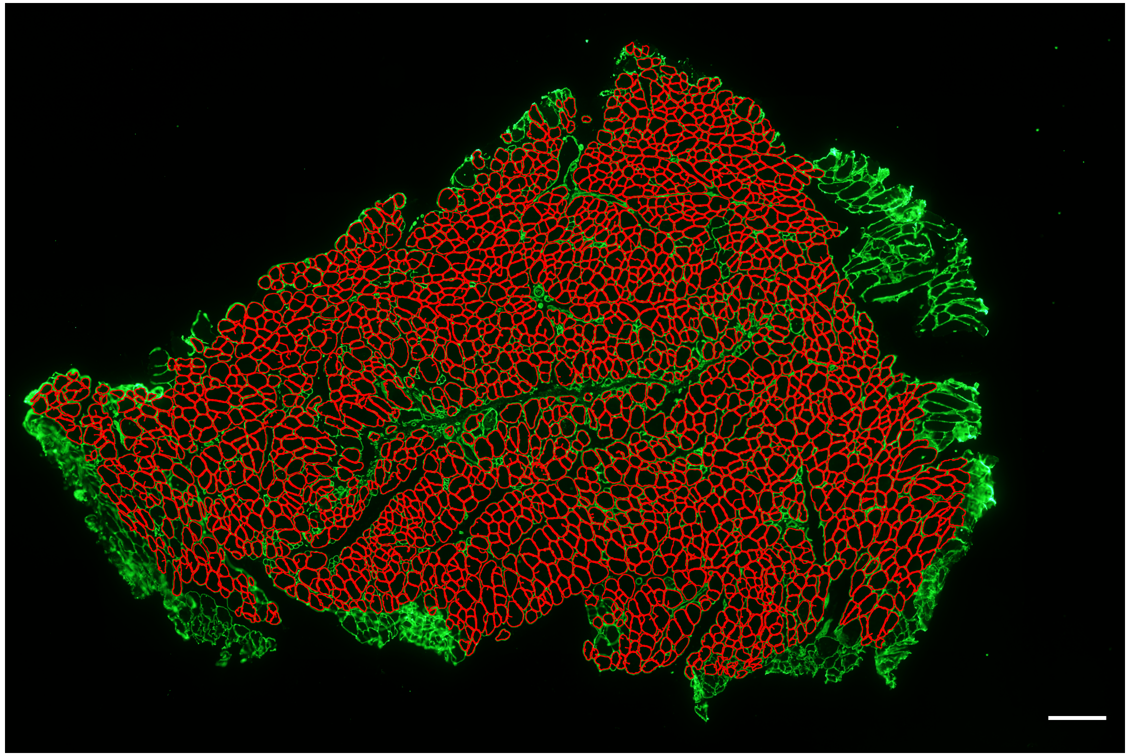

spinning

mdx 11

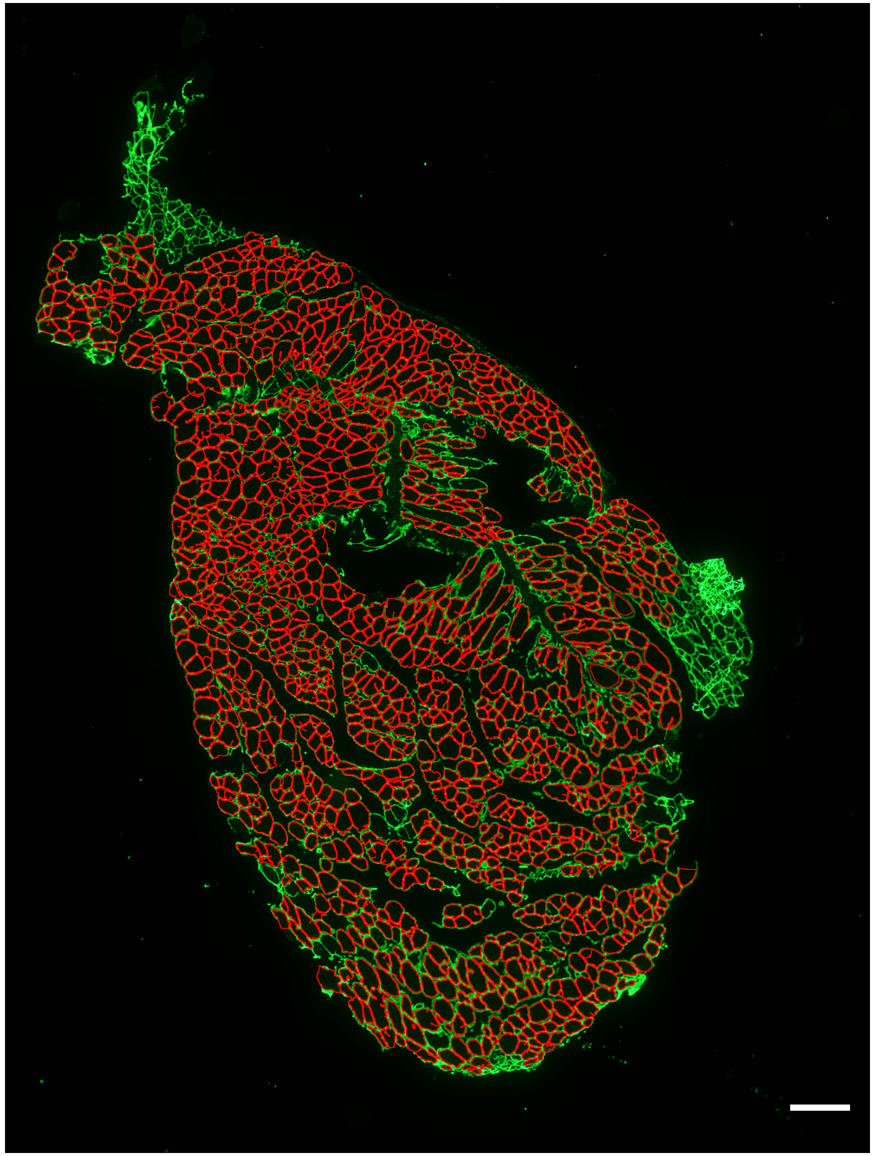

spinning

mdx 12

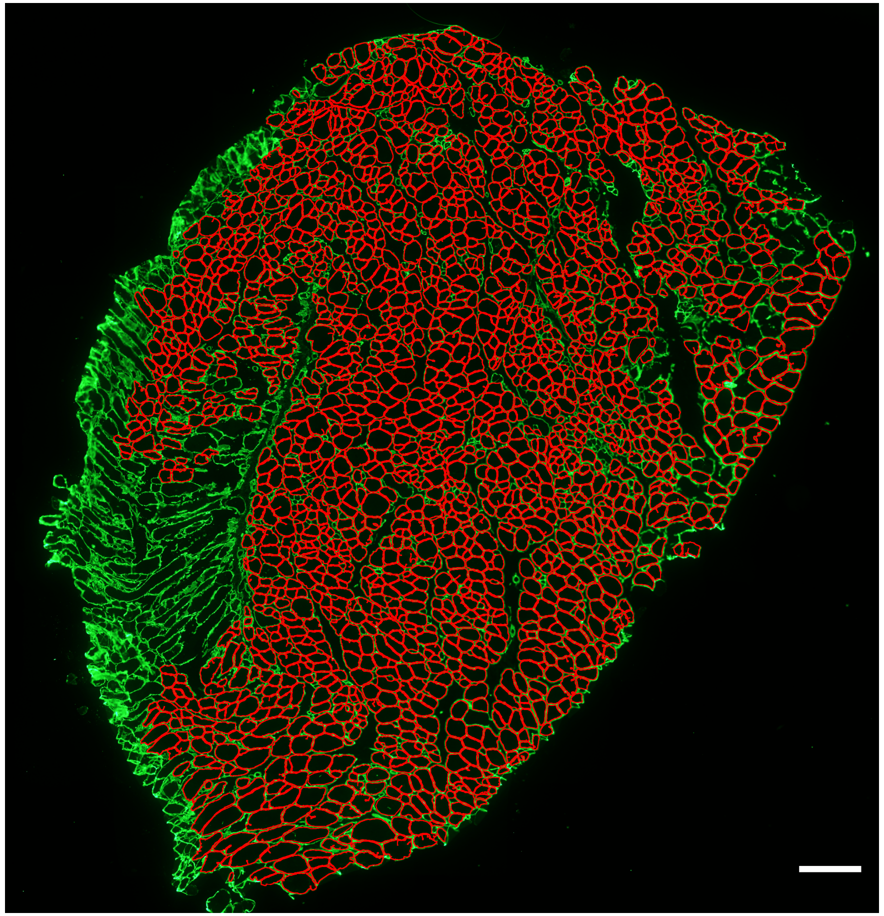

spinning

mdx 13

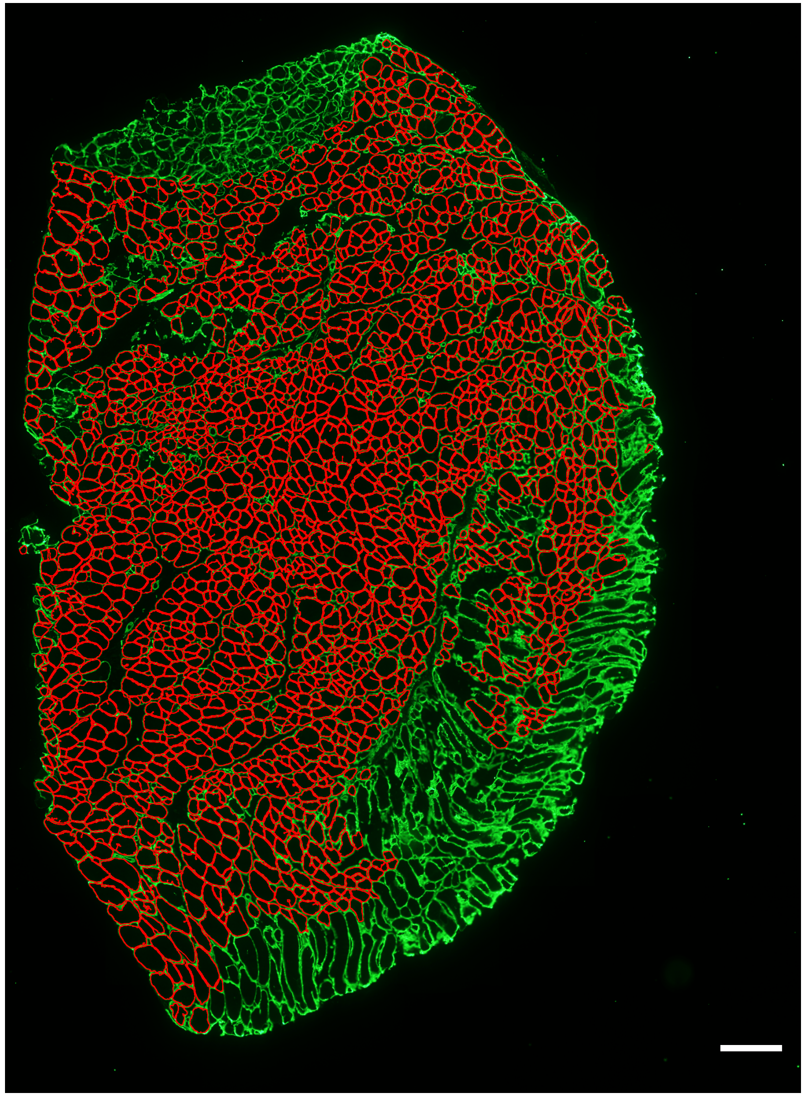

spinning

MDX+W

Mdx+W 8G

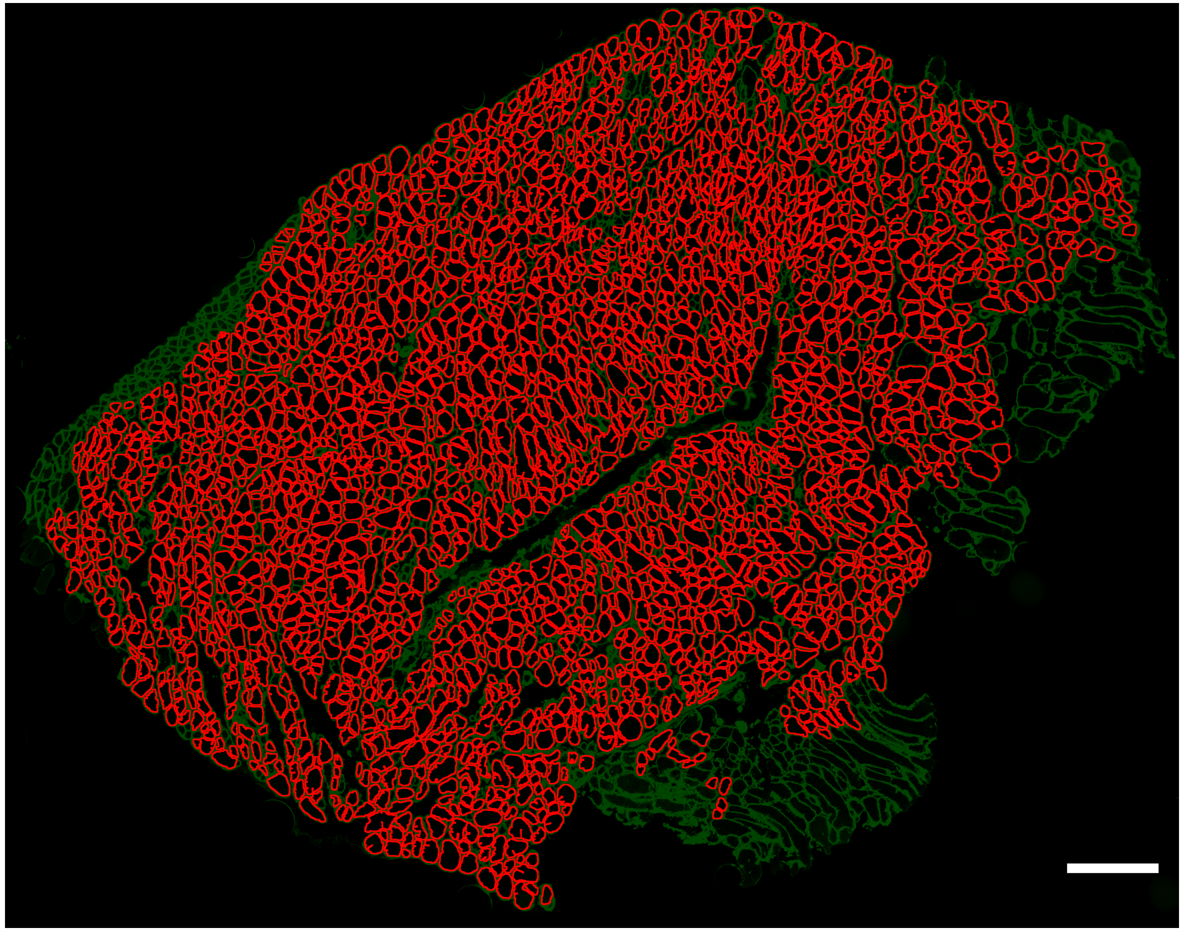

spinning

Mdx+W 3G

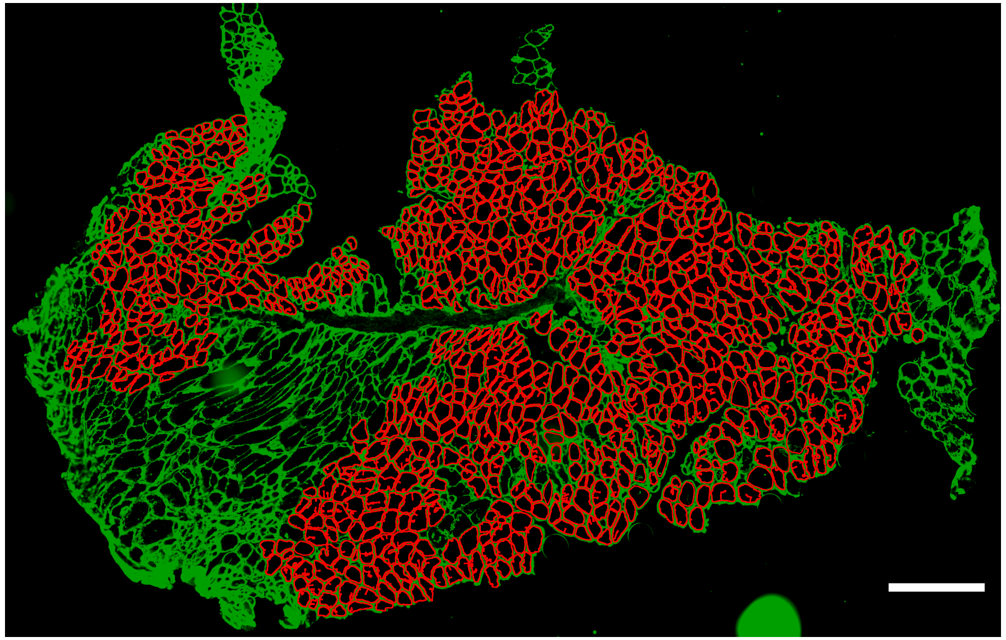

nanozoomer

mdx W 1G

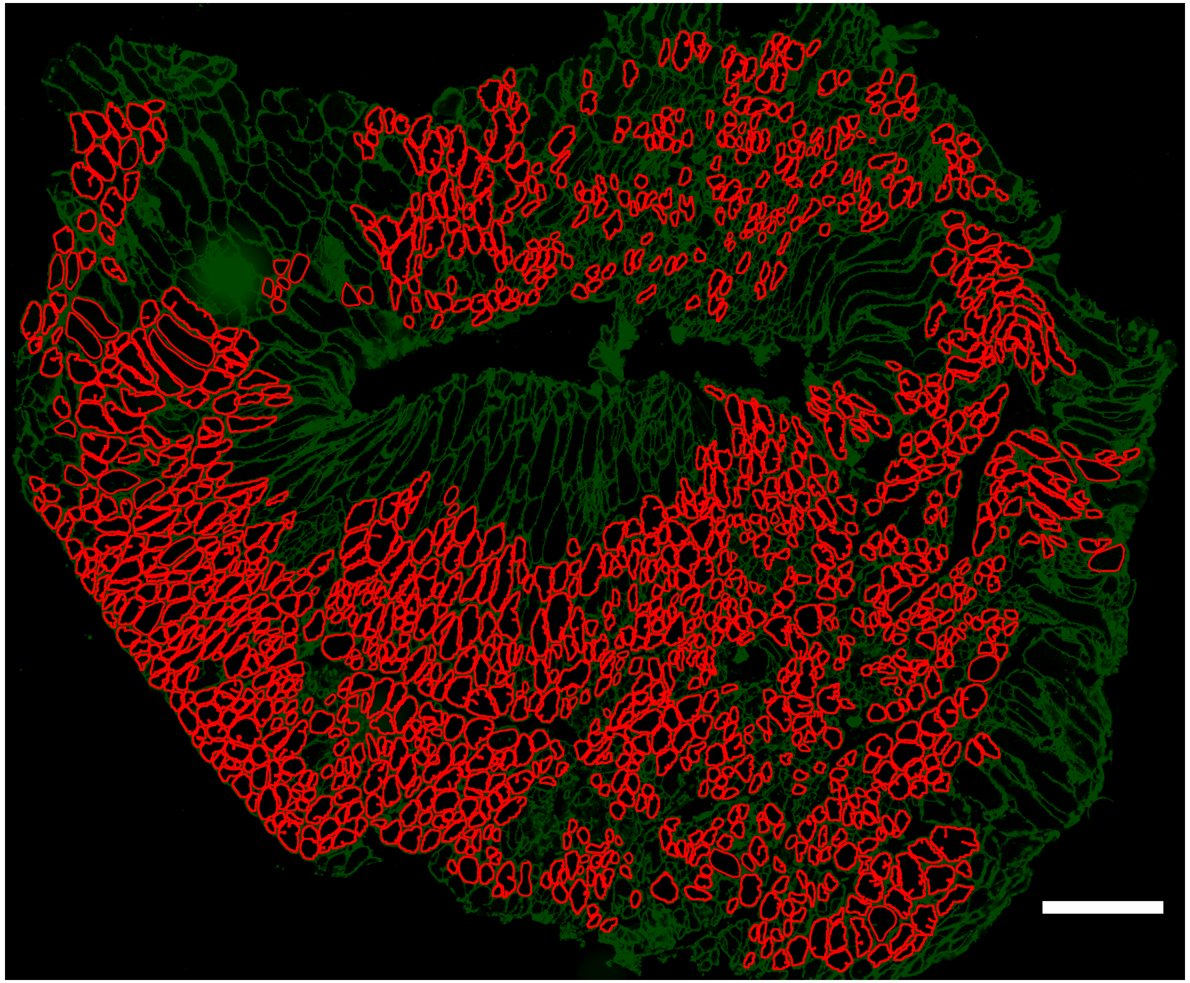

spinning

mdx W 6G

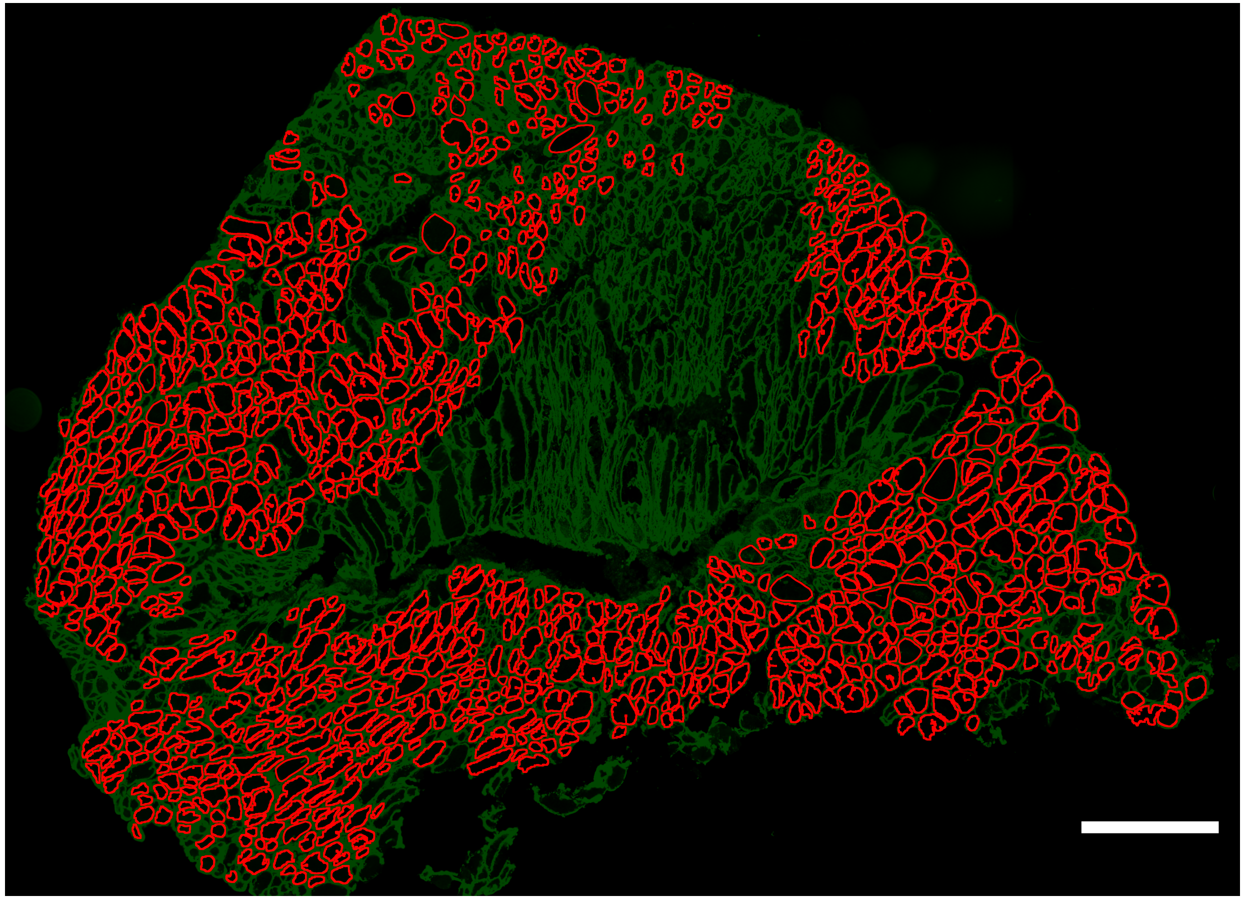

nanozoomer

mdx W 8G

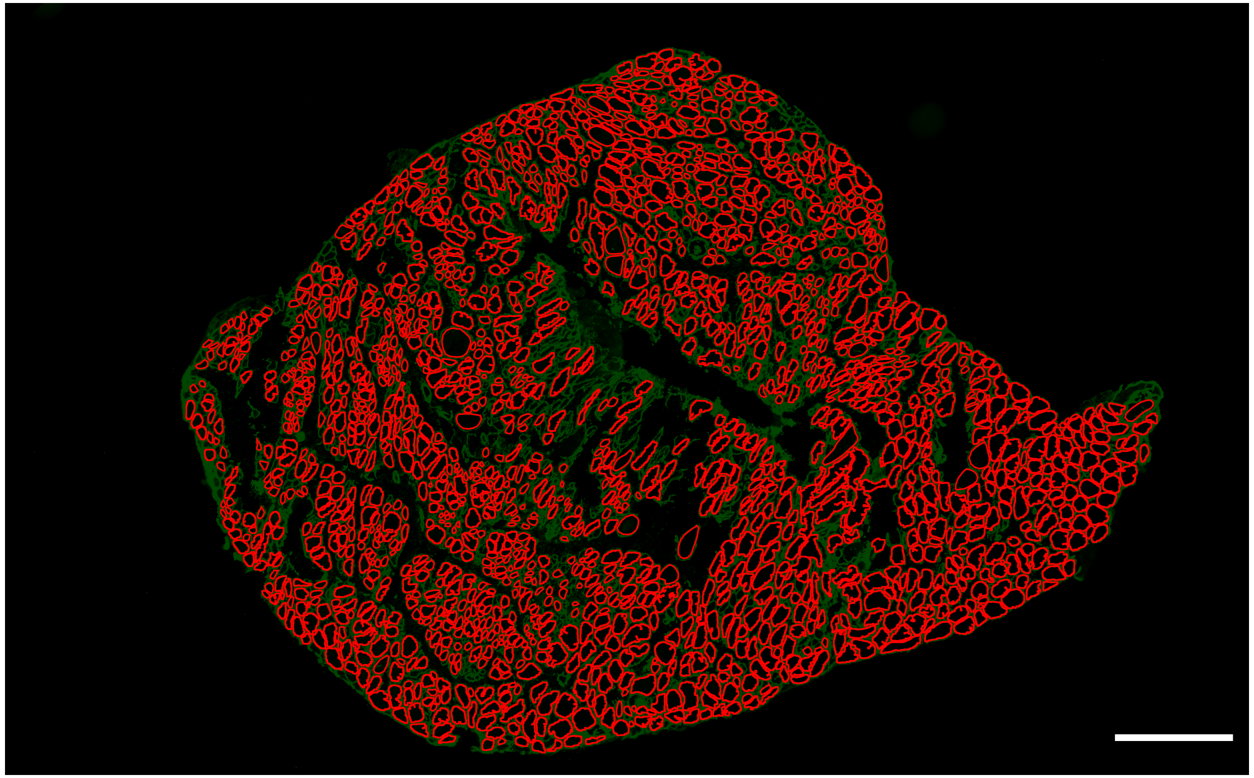

mdx W P 8D

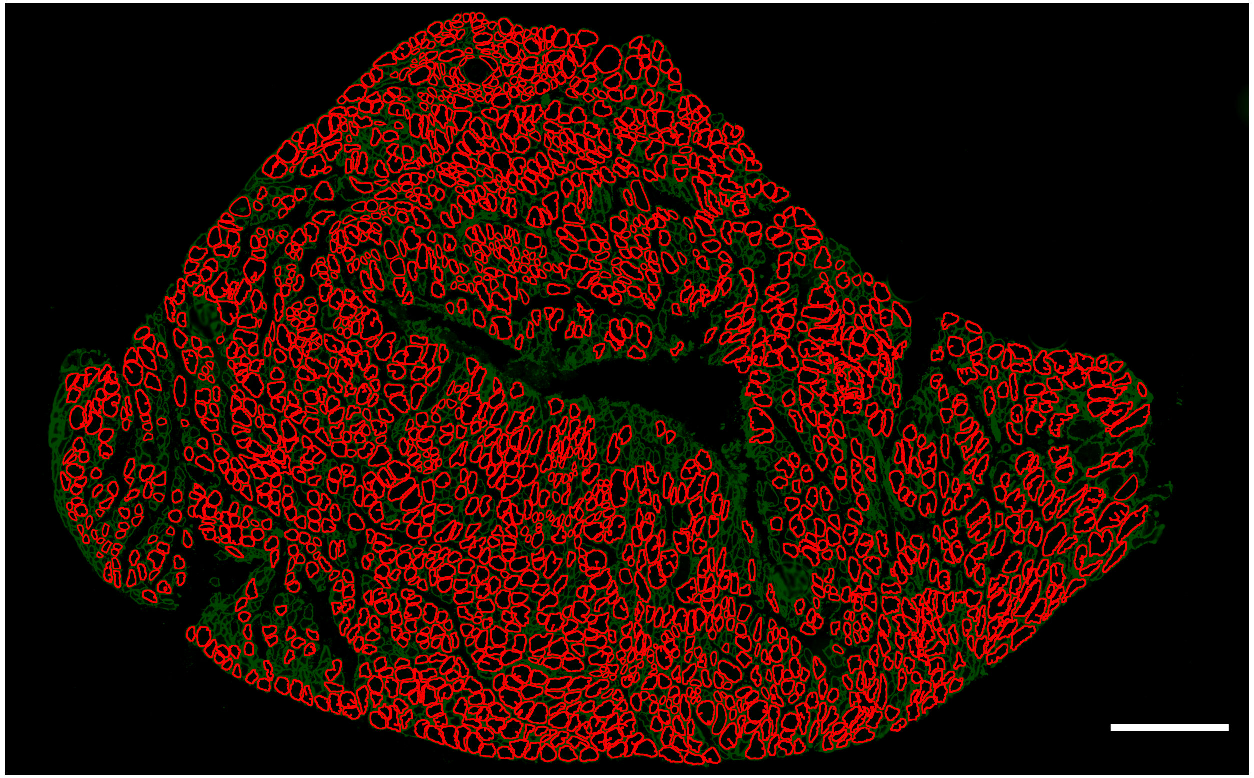

MDX+W+ P

Mdx+W 8G

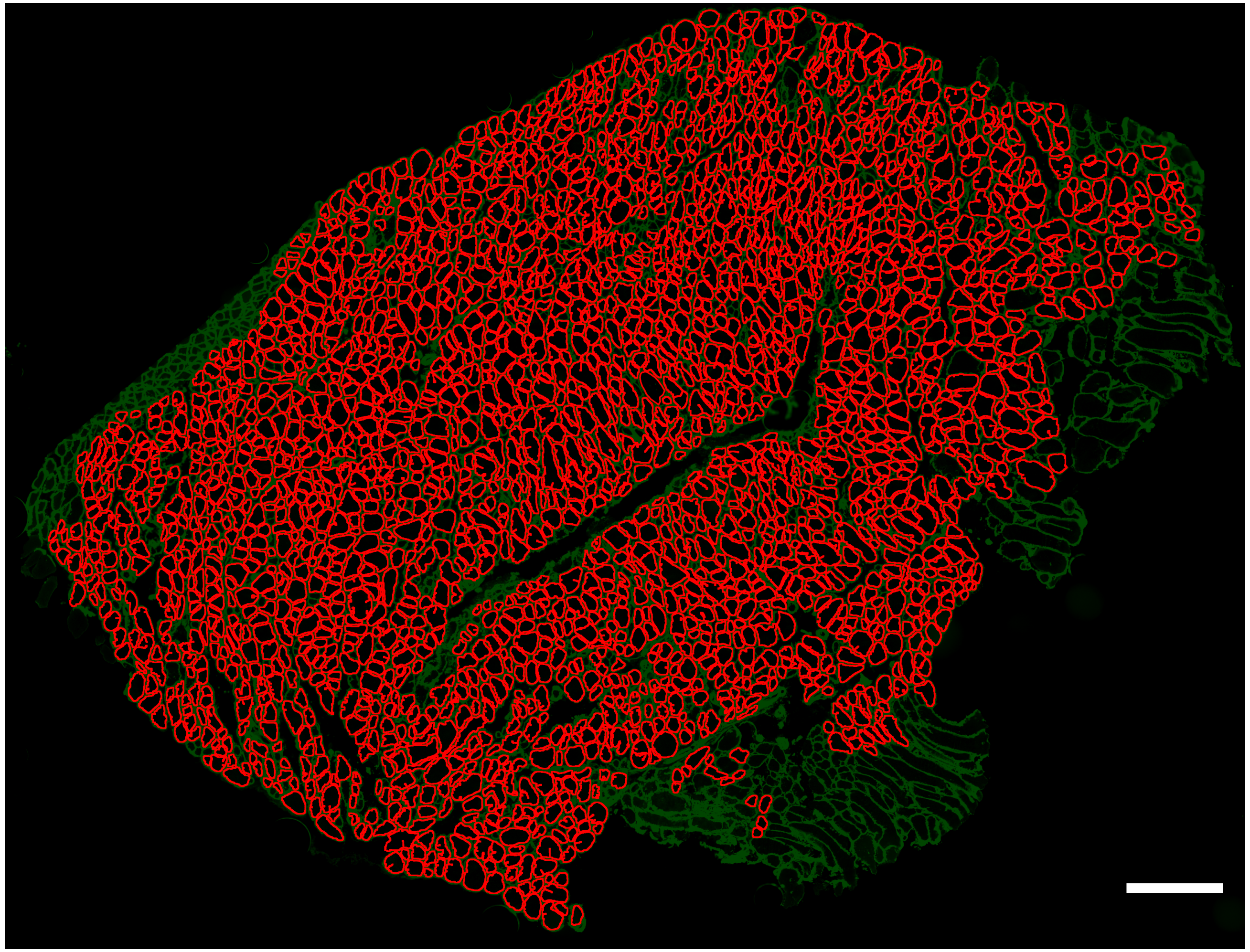

Mdx+W+P 8D

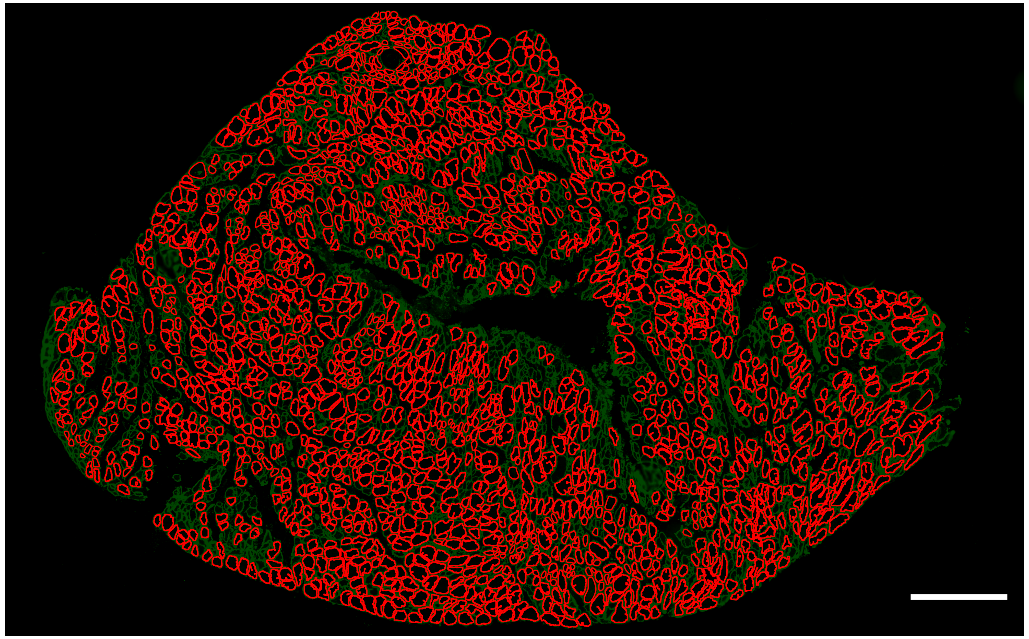

mdx W+P 3D

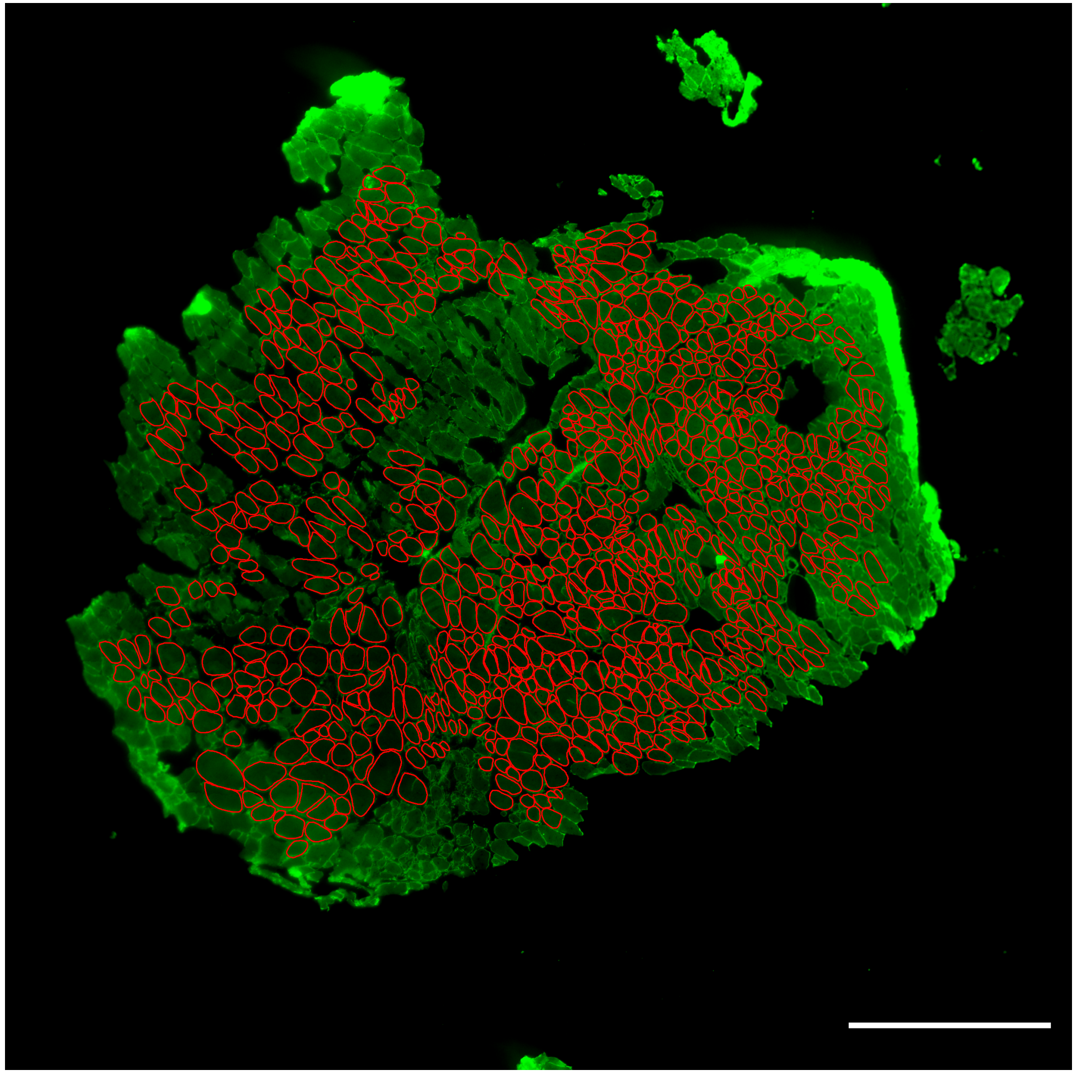

(40X) *spinning*
