# Supplementary material for: The beneficial effect of chronic muscular exercise on muscle fragility is increased by Prox1 gene transfer in dystrophic mdx muscle
Source: PLoS One. 2022 Apr 18;17(4):e0254274. doi: 10.1371/journal.pone.0254274 (PMC9015141; doi:10.1371/journal.pone.0254274)
Supplement: S5 Fig — (PDF) [file pone.0254274.s005.pdf]

Scale bar 500um (20X)

SET 2

MDX

mdx 4D

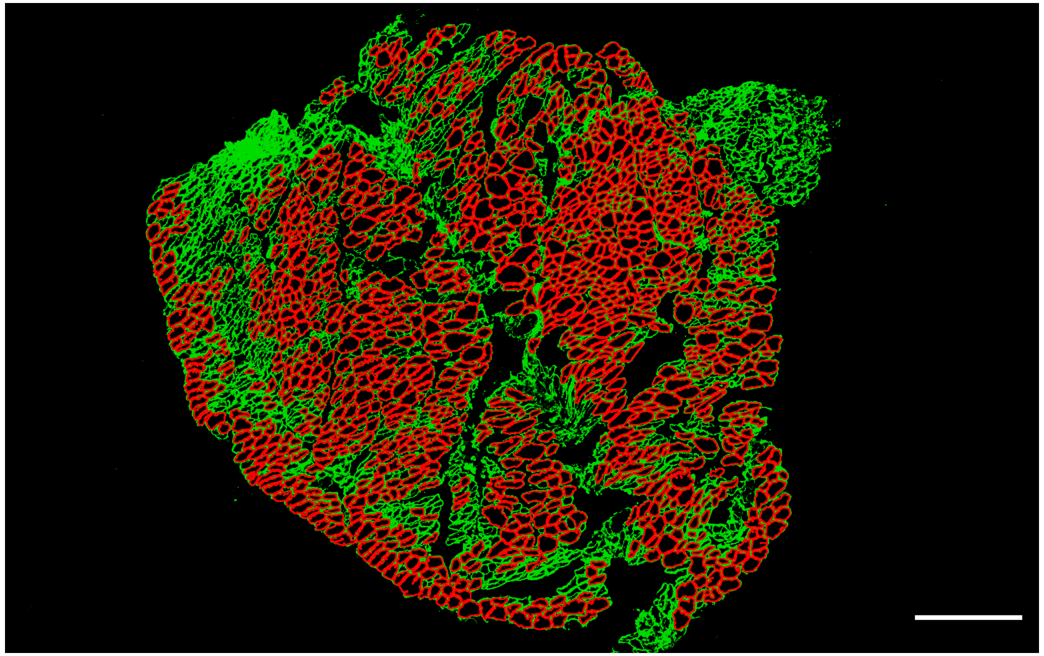

nanozoomer

mdx 5D

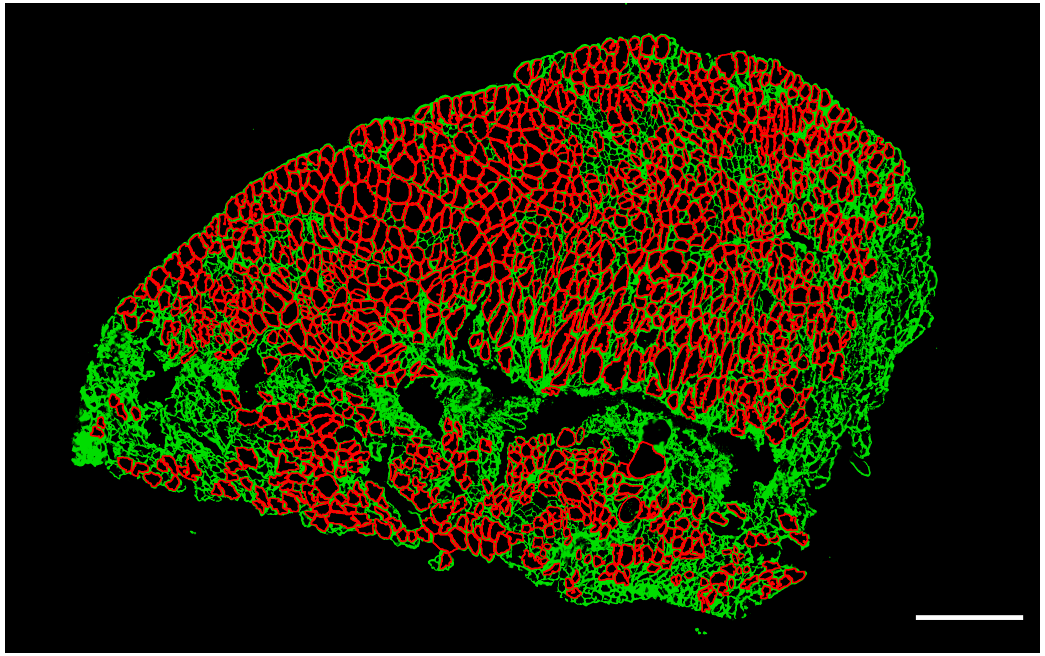

nanozoomer

mdx 8D

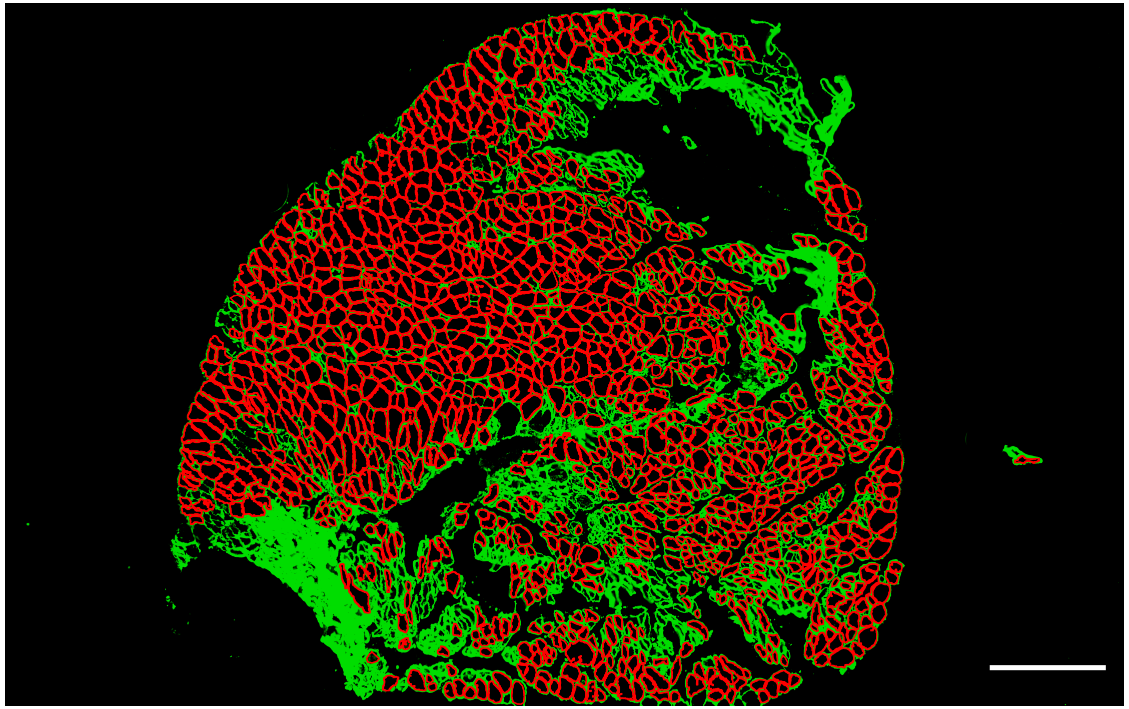

nanozoomer

MDX+P

mdx P 4G

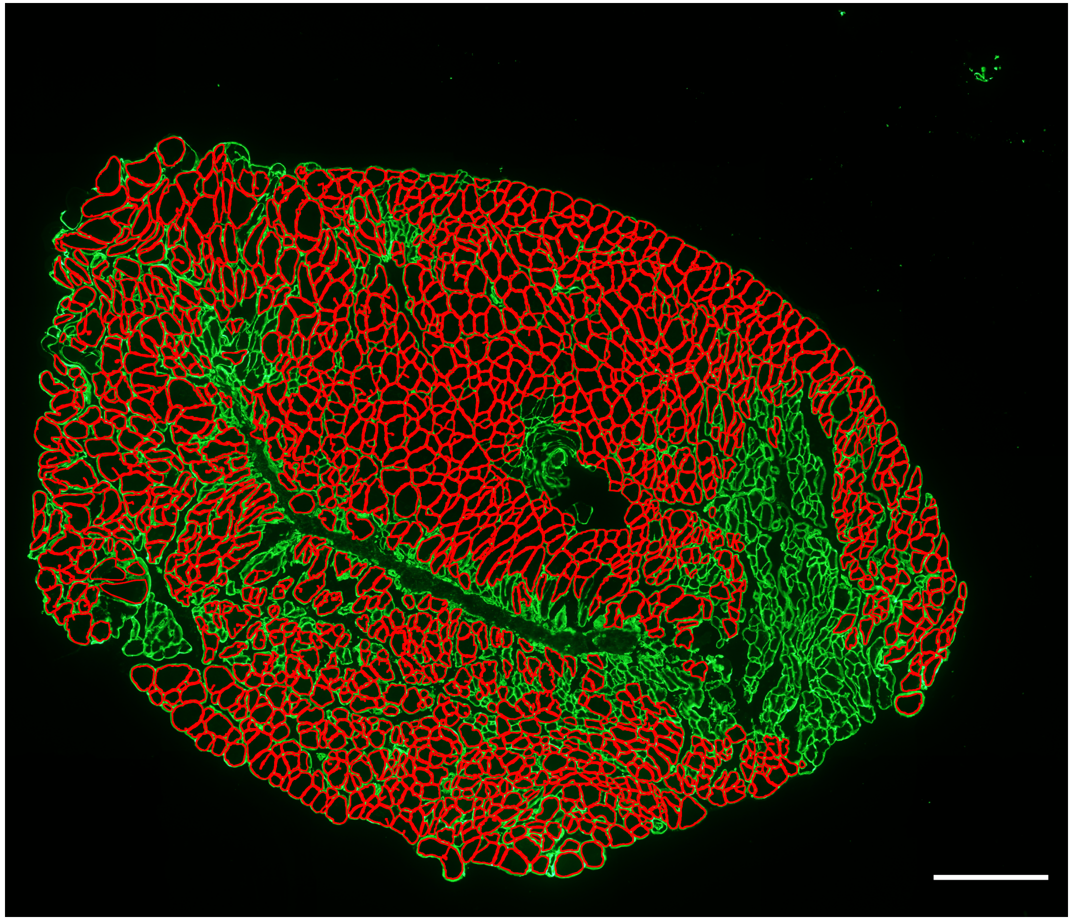

nanozoomer

mdx P 8G

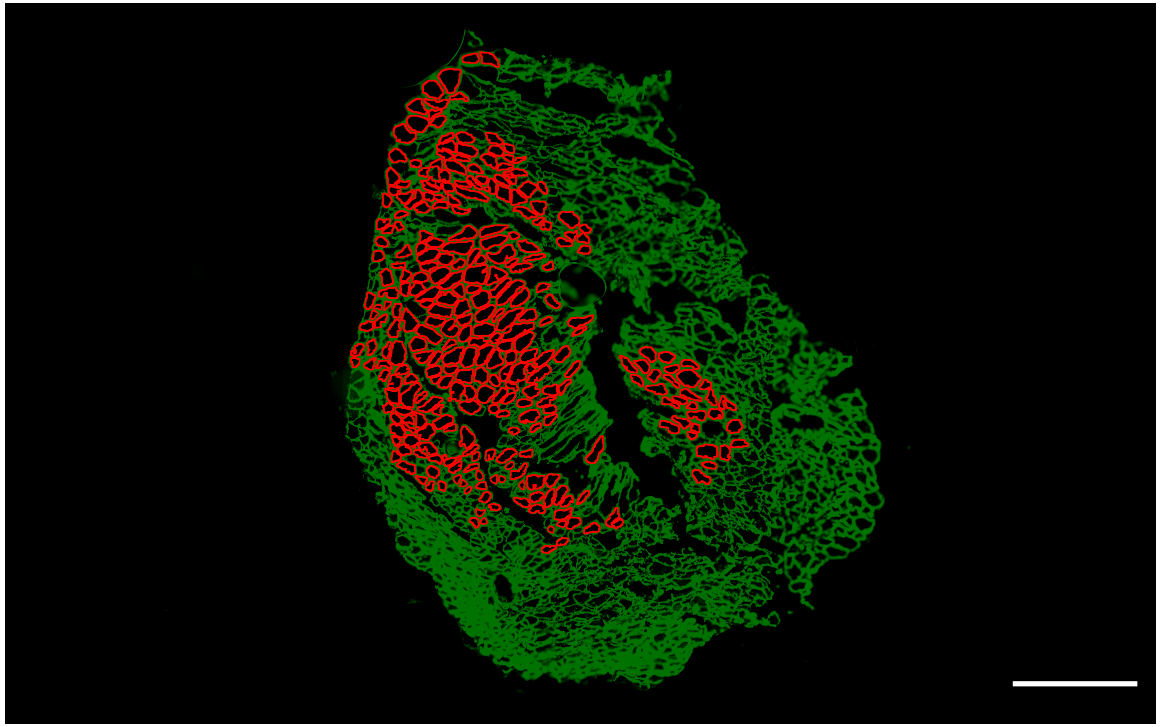

nanozoomer

mdx P 4D (série set 1)

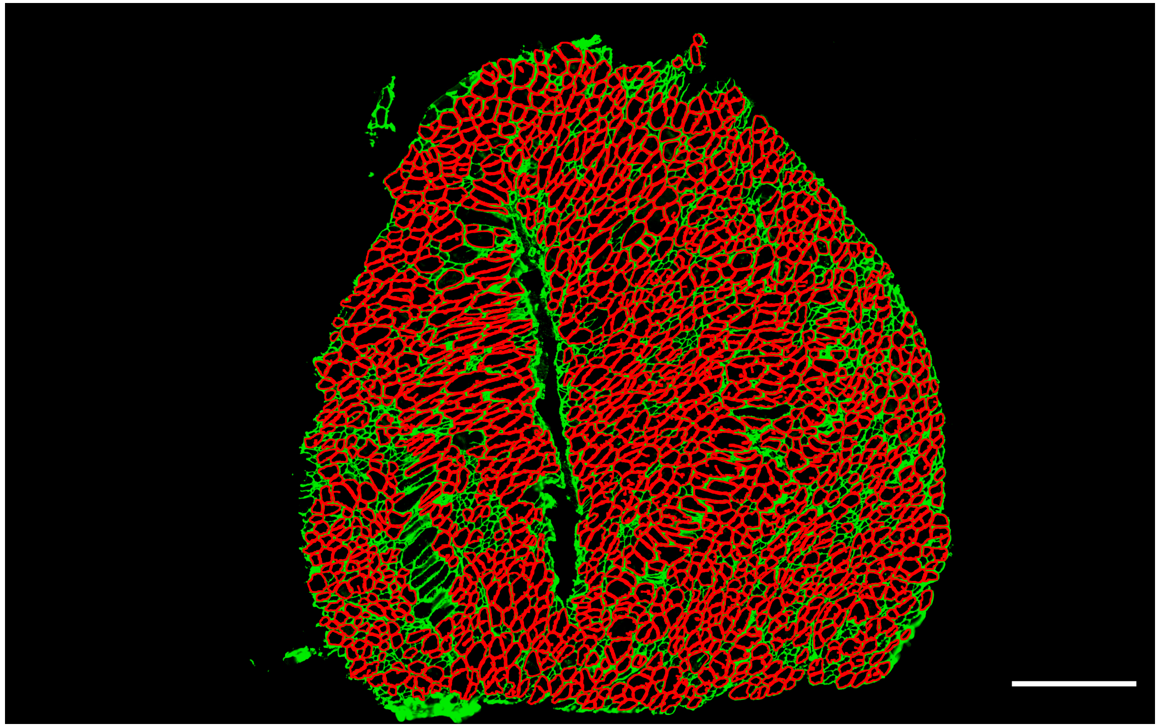

nanozoomer
