# Supplementary material for: The beneficial effect of chronic muscular exercise on muscle fragility is increased by Prox1 gene transfer in dystrophic mdx muscle
Source: PLoS One. 2022 Apr 18;17(4):e0254274. doi: 10.1371/journal.pone.0254274 (PMC9015141; doi:10.1371/journal.pone.0254274)
Supplement: S2 Table — (PDF) [file pone.0254274.s007.pdf]

MHC electrophoresis

SET 2

	mdx			mdx + P	
MHC-2x (%)	24,89	33,35	34,2	29,51	28,07
MHC-2b (%)	75,11	66,65	65,8	70,49	71,93

SET 1

	mdx + W			mdx + W + P	
MHC-2x (%)	24,01	22,58	27,64	35,76	45,45
MHC-2b (%)	75,99	77,42	72,36	64,24	54,55

no MHC-2a band

25,02
74,98

38,68
61,32
