# Supplementary material for: The beneficial effect of chronic muscular exercise on muscle fragility is increased by Prox1 gene transfer in dystrophic mdx muscle
Source: PLoS One. 2022 Apr 18;17(4):e0254274. doi: 10.1371/journal.pone.0254274 (PMC9015141; doi:10.1371/journal.pone.0254274)
Supplement: S3 Table — (PDF) [file pone.0254274.s008.pdf]

SET 1

N° Souris	P0 (g)	%	Pic 4	%	Pic 7	%	Pic 10	%	Body weight<br>poids souris	Muscle weight<br>Masse TA (mg)		sP0 (g/mg)	Lengthening force (g)<br>Force excentrique	%/P0
	Isometric Force													
**mdx + W + P**														
1(injectée)	56,0	100,0	44,3	79,1	35,7	63,8	35,6	63,6	30,5	55,2		1,0	132	236
3(injectée)	80,0	100,0	67,7	84,6	49,3	61,6	39,3	49,1	29,9	54,7		1,5	163	204
5(injectée)	78,2	100,0	59,2	75,7	41,5	53,1	31,9	40,8	30,1	57,6		1,4	154	197
6(injectée)	109,0	100,0	66,8	61,3	37,6	34,5	27,4	25,1	28,0	57,2		1,9	184	169
7(injectée)	85,4	100,0	72,3	84,7	63,5	74,4	55,9	65,5	30,4	59,8		1,4	143	167
8(injectée)	93,5	100,0	85,4	91,3	61,1	65,3	41,7	44,6	30,2	63,6		1,5	167	179
**mdx + W**														
1(non injectée)	138,3	100,0	96,7	69,9	54,4	39,3	42,7	30,9		77,7		1,8	248	179
4 ? (non injectée)	138,5	100,0	77,1	55,7	28,5	20,6	17,9	12,9		86,3		1,6		
5(non injectée)	119,3	100,0	88,0	73,8	67,1	56,2	56,9	47,7		90,3		1,3	239	200
6(non injectée)	116,9	100,0	83,1	71,1	51,9	44,4	40,3	34,5		81,7		1,4	198	169
7(non injectée)	136,1	100,0	96,7	71,1	60,1	44,2	44,2	32,5		89,4		1,5	236	173
8(non injectée)	155,8	100,0	130,6	83,8	56,5	36,3	33,5	21,5		62,8		2,5	279	179
2(non injectée)	123,9	100,0	105,5	85,1	82,9	66,9	67,4	54,4	29,9	76,0		1,6	213	172
2(non injectée)	111,2	100,0	80,7	72,6	62,5	56,2	34,8	31,3	29,9	67,0		1,7	215	193
**mdx**														
9(non injectée)	146,8	100,0	73,7	50,2	29,3	20,0	20,5	14,0		101,3		1,4	242	165
(non injectée)	172,7	100,0								109,3		1,6	294	170
10(non injectée)	111,3	100,0	48,5	43,6	16,6	14,9	12,1	10,9		93,4		1,2	203	182
(non injectée)	166,5	100,0	71,8	43,1	17,4	10,5	10,8	6,5		94,7		1,8	286	172
11(non injectée)	111,6	100,0	56,9	51,0	25,9	23,2	21,8	19,5		110,5		1,0	180	161
(non injectée)	179,2	100,0	104,2	58,1	29,3	16,4	14,3	8,0		118,2		1,5	294	164
12(non injectée)	157,6	100,0	78,6	49,9	16,2	10,3	9,0	5,7		100,5		1,6	268	170
(non injectée)	153,8	100,0	77,9	50,7	37,6	24,4	18,1	11,8		96,0		1,6	281	183

SET 2

N° Souris	Patte	P0 (g)	%	pic 2	%	pic3	%	pic 4	%	pic 5	%	pic 6	%	pic 7	%	pic 8	%	pic 9	%	pic 10	%	Muscle weight<br>Masses (mg)	sP0<br>Force	Lengthening force (g)<br>force excentrique	%/P0
		Isometric Force																							
**mdx**																									
4415	droite																					113			
4413	droite	114,60	100,00	94,60	82,55	87,60	76,44	82,30	71,82	72,50	63,26	62,20	54,28	52,10	45,46	42,60	37,17	35,10	30,63	27,10	23,65	96	1,19	202	176
4420	droite	157,20	100,00	118,20	75,19	91,30	58,08	71,60	45,55	52,60	33,46	38,60	24,55	27,10	17,24	20,40	12,98	14,70	9,35	11,20	7,12	92,3	1,70	271	172
4406	droite	153,80	100,00	130,70	84,98	113,20	73,60	87,30	56,76	59,30	38,56	38,50	25,03	25,10	16,32	17,30	11,25	13,00	8,45	9,60	6,24	86,5	1,78	287	187
4409	droite	128,39	100,00	98,52	76,73	84,30	65,66	74,75	58,22	64,11	49,93	51,83	40,37	40,98	31,92	31,50	24,53	24,08	18,75	18,22	14,19	86,5	1,48	222	173
4407	droite	131,13	100,00	108,11	82,45	91,22	69,57	73,13	55,77	49,89	38,05	45,47	34,68	50,14	38,24	49,50	37,75	46,59	35,53	43,45	33,14	88,1	1,49	297	227
4408	droite	129,30	100,00																			81,2	1,59	263	203
**mdx + P**																									
4415	gauche	52,80	100,00	42,70	80,87	38,30	72,54	36,40	68,94	34,50	65,34	33,50	17,69	32,80	62,12	30,90	58,52	29,60	56,06	28,70	54,36	72,2	0,73	102	193
4413	gauche	104,60	100,00	89,80	85,85	71,60	68,45	56,90	54,40	45,20	43,21	39,80	38,05	34,90	33,37	30,90	29,54	28,60	27,34	26,30	25,14	67,8	1,54	229	219
4420	gauche	87,40	100,00	78,90	90,27	71,40	81,69	61,60	70,48	51,40	58,81	40,70	46,57	31,70	36,27	26,00	29,75	21,30	24,37	17,80	20,37	68	1,29	206	236
4406	gauche	120,10	100,00	104,70	87,18	89,70	74,69	70,70	58,87	55,90	46,54	44,20	36,80	36,90	30,72	32,80	27,31	27,90	23,23	24,70	20,57	81,9	1,47	238	198
4409	gauche	143,13	100,00	103,42	72,26	71,41	49,89	40,05	27,98	20,92	14,62	10,41	7,27	6,53	4,56	4,28	2,99	1,77	1,23	0,97	0,68	69,8	2,05	255	178
4407	gauche	86,70	100,00	76,59	88,34	40,05	46,19	71,53	82,50	65,14	75,13	59,28	68,37	56,03	64,62	51,30	59,16	47,61	54,91	43,39	50,05	92,3	0,94	199	230
4408	gauche	145,90	100,00																			61,2	2,38	212	145
