# Supplementary material for: The beneficial effect of chronic muscular exercise on muscle fragility is increased by Prox1 gene transfer in dystrophic mdx muscle
Source: PLoS One. 2022 Apr 18;17(4):e0254274. doi: 10.1371/journal.pone.0254274 (PMC9015141; doi:10.1371/journal.pone.0254274)
Supplement: S1 Table — (PDF) [file pone.0254274.s006.pdf]

mRNA

					calcineurin signalling	type fibres				canaux ions avec dystro					NOX2	nadph oxydase					Atrophy													Ref			
			Muscles	sdha	prox1	myh7	myh2	myh4	tnni1	snc4a	cacn1s	slc8a1	TRPC1	chrna1	prxII	gp91phox	p47phox	rac1	UTRN	desmin	murf1	Mafbox/atrogin1	LC3	gadd45	hdac4	Fn14	JunB	redd1	redd2	MSTN	follistatin	igf1	smox	hmbs	Muscles		
SET 2	16/11/2018	Sed+PBS	MdxSed-TA-1D	0,9	1,7	0,6	0,3	1,2	0,5	1,0	1,0	1,3	1,4	0,7	0,9	0,9	0,7	1,1	0,9	1,0	0,9	0,8	0,9	0,8	0,9	1,3		0,6	1,4	1,2	1,0	1,2	0,5	1,1	MdxSed-TA-1D	Sed+PBS	16/11/2018
			MdxSed-TA-2D	1,1	0,8	0,6	0,1	1,4	0,5	1,1	1,1	1,0	0,8	0,6	0,9	0,4	0,4	1,1	0,9	1,1	1,0	0,8	0,9	0,9	0,8	1,4		1,7	1,1	1,2	1,0	0,9	1,2	1,2	MdxSed-TA-2D		
			MdxSed-TA-3D	1,2	0,3	2,2	3,1	0,9	0,5	1,1	1,1	1,2	1,2	0,7	1,4	1,0	0,8	0,8	1,2	1,0	0,9	0,9	1,4	0,8	1,0	1,2		1,3	1,2	1,1	1,0	1,1	1,2	1,0	MdxSed-TA-3D		
			MdxSed-TA-4D	0,9	1,0	0,9	2,0	0,7	1,1	1,1	0,9	0,9	1,0	0,6	0,9	1,5	1,1	1,3	1,0	0,7	1,1	0,9	0,8	0,8	1,1	0,7		0,4	0,6	0,8	0,9	0,9	1,3	0,6	MdxSed-TA-4D		
			MdxSed-TA-5D	1,0	1,6	2,1	1,6	0,9	0,6	1,0	1,0	0,7	1,0	0,8	1,0	0,8	1,1	1,1	1,0	1,0	1,0	1,0	0,9	0,9	1,1	0,8		0,5	0,7	0,7	0,9	0,8	0,5	1,1	MdxSed-TA-5D		
			MdxSed-TA-6D	1,2	2,3	0,6	0,3	1,4	0,5	1,2	1,2	0,7	0,9	0,8	0,9	0,6	0,6	0,9	0,9	0,9	1,2	1,2	1,2	1,3	1,1	0,5		0,9	1,4	1,1	0,8	0,7	1,7	1,2	MdxSed-TA-6D		
			MdxSed-TA-7D	0,9	0,3	0,7	0,8	0,9	1,5	0,8	1,0	1,1	1,1	1,5	0,8	1,3	1,1	1,1	0,8	0,8	1,2	1,6	0,9	1,1	1,0	0,9		1,0	0,9	1,0	1,0	0,9	0,8	0,8	MdxSed-TA-7D		
			MdxSed-TA-8D	1,1	1,1	1,0	0,9	1,0	4,2	0,7	0,9	1,1	1,0	2,6	1,1	1,8	1,4	1,1	1,1	1,0	0,8	1,0	0,9	1,1	0,8	1,0		0,8	1,1	0,8	1,0	1,3	0,7	0,7	MdxSed-TA-8D		
			MdxSed-TA-3G	0,9	0,3	0,9	0,5	0,6	0,4	1,0	0,9	1,3	1,0	1,0	1,2	1,0	1,5	0,7	1,0	1,0	0,7	0,7	1,0	0,6	1,0	1,6		1,0	0,9	1,0	1,3	1,6	0,9	1,2	MdxSed-TA-3G		
			MdxSed-TA-7G	0,8	0,7	0,7	0,2	0,9	0,6	1,0	0,9	0,7	1,0	0,9	0,9	0,7	1,5	0,7	0,9	0,7	1,5	1,1	1,0	1,3	1,0	1,0		1,3	0,7	1,4	0,8	0,9	1,2	1,0	MdxSed-TA-7G		
			Mdx+R+Prox1-TA-4G	1,0	0,8	0,8	1,1	1,0	0,7	1,0	0,9	0,8	0,7	0,9	1,0	0,9	0,9	1,1	1,1	1,7	0,8	1,1	1,0	1,3	1,0	0,6		1,6	1,0	0,7	1,3	0,8	1,0	0,9	Mdx+R+Prox1-TA-4G		
		Sed+Prox1	MdxSed-TA-1G	0,9	24,9	4,4	0,2	0,6	2,9	0,8	1,0	0,9	1,6	1,8	1,1	1,8	1,2	1,3	0,9	1,1	1,1	0,5	1,2	1,0	0,8	3,0		0,2	0,8	0,5	1,4	1,3	0,7	1,4	MdxSed-TA-1G	Sed+Prox1	
			MdxSed-TA-2G	0,8	25,0	1,4	0,7	0,7	2,2	0,8	1,2	1,0	1,1	2,0	1,1	3,2	0,8	1,3	1,0	1,2	0,9	0,4	1,1	1,4	1,0	3,2		0,8	0,6	0,6	1,2	1,0	0,7	1,4	MdxSed-TA-2G		
			MdxSed-TA-4G	0,8	31,6	21,0	0,4	0,4	4,9	0,5	1,3	1,5	2,0	2,9	1,5	0,4	1,0	0,4	1,1	1,2	0,8	0,5	1,1	1,2	1,0	1,9		0,2	0,7	0,5	1,5	1,2	0,1	2,3	MdxSed-TA-4G		
			MdxSed-TA-6G	0,9	28,5	3,5	0,9	0,8	2,3	0,9	1,2	0,7	1,4	1,7	1,1	3,7	0,9	1,1	1,1	1,3	1,1	0,8	1,1	1,7	1,0	1,7		0,6	0,9	0,6	1,3	0,9	0,1	1,0	MdxSed-TA-6G		
			MdxSed-TA-8G	0,8	28,9	6,6	0,4	0,6	3,0	0,7	1,0	0,9	1,4	1,5	1,1	0,6	2,0	0,6	1,2	1,1	0,9	0,6	1,3	1,2	0,8	1,4		0,6	0,7	0,5	0,9	0,7	0,5	1,2	MdxSed-TA-8G		
			Mdx+R+Prox1-TA-4D	0,7	25,2	0,4	0,1	1,0	1,9	1,0	1,2	0,7	1,1	1,3	0,9	1,4	1,5	1,1	1,3	2,2	1,2	0,7	1,1	1,9	1,6	1,7		1,1	0,7	0,7	1,1	0,6	0,8	0,7	Mdx+R+Prox1-TA-4D		
																																		0,0			
SET 1	17/05/2018	Sed pas injecté PBS	Mdx+R+Prox1-TA-9G	1,0	0,9	0,3	0,3	1,2	1,2	1,0	1,1	0,9	0,7	1,1	1,0	0,8	0,5	0,9	0,8	1,1	0,7	0,9	0,9	0,8	0,9	1,1		0,4	1,2	0,8	0,7	1,0	1,4	0,8	Mdx+R+Prox1-TA-9G	Sed pas injecté PBS	17/05/2018
			Mdx+R+Prox1-TA-10G	1,1	1,1	0,1	0,4	1,2	0,8	1,2	1,0	1,1	0,7	0,9	1,2	0,6	0,5	1,1	1,0	1,2	1,0	1,1	1,3	0,8	0,9	1,2		0,8	1,0	1,4	0,9	1,0	1,0	1,5	Mdx+R+Prox1-TA-10G		
			Mdx+R+Prox1-TA-11G	1,1	1,6	4,1	0,7	1,2	0,6	0,9	0,9	1,1	1,0	1,0	1,0	0,6	0,5	1,0	1,0	1,0	1,6	1,2	1,2	0,8	0,9	0,6		2,1	1,1	0,9	0,9	0,9	1,7	1,0	Mdx+R+Prox1-TA-11G		
			Mdx+R+Prox1-TA-12G	1,2	1,4	0,7	4,3	0,7	1,3	0,8	0,8	0,9	0,9	1,2	1,3	0,7	0,4	1,0	1,0	1,0	0,8	1,1	1,0	0,8	1,3	0,6		1,1	1,0	0,9	1,3	0,8	0,5	1,2	Mdx+R+Prox1-TA-12G		
			Mdx+R+Prox1-TA-9D	0,9	0,1	1,5	0,8	0,8	1,3	1,1	1,2	0,7	2,7	0,5	0,8	1,4	0,2	0,7	1,1	0,7	0,7	0,6	0,7	0,6	0,9	1,4		0,5	0,6	0,7	0,5	1,2	0,8	0,0	Mdx+R+Prox1-TA-9D		
			Mdx+R+Prox1-TA-10D	0,8	0,9	0,3	0,3	1,0	0,9	1,2	1,4	1,0	0,8	1,2	0,8	2,2	4,3	1,3	1,3	1,1	1,0	0,9	0,8	2,4	1,2	1,5		0,5	0,8	0,8	2,0	1,3	0,9	1,2	Mdx+R+Prox1-TA-10D		
			Mdx+R+Prox1-TA-11D	1,1	1,1	0,2	0,3	1,1	0,9	1,0	0,7	1,1	0,7	1,1	1,0	0,6	0,7	1,1	0,9	0,9	1,6	1,4	1,3	0,9	0,8	1,0		1,7	1,1	1,3	0,8	0,9	0,8	1,4	Mdx+R+Prox1-TA-11D		
			Mdx+R+Prox1-TA-12D	0,9	0,9	0,7	1,0	0,8	1,2	0,7	0,8	1,2	0,6	1,0	1,0	1,1	1,0	0,9	0,9	1,1	0,6	0,8	0,8	0,9	1,0	0,5		0,9	1,1	1,2	0,9	1,0	0,9	0,9	Mdx+R+Prox1-TA-12D		
		Roue + PBS	Mdx+R+Prox1-TA-1G	1,2	2,9	2,8	1,0	1,2	1,2	1,3	1,0	1,0	0,9	1,4	1,2	2,1	1,2	1,2	1,3	1,1	0,9	0,6	1,2	0,8	1,0	1,6		0,8	1,0	0,8	1,4	1,1	1,8	1,2	Mdx+R+Prox1-TA-1G	Roue + PBS	
			Mdx+R+Prox1-TA-2G	1,0	2,5	1,9	1,8	0,7	4,4	0,9	0,8	0,8	0,9	2,6	1,4	2,5	1,5	1,1	1,5	0,9	0,8	0,6	1,2	0,9	1,0	1,1		1,6	0,9	0,7	1,5	2,0	0,7	1,9	Mdx+R+Prox1-TA-2G		
			Mdx+R+Prox1-TA-3G	0,9	6,1	2,1	1,6	1,0	1,9	1,2	0,9	1,2	0,7	1,6	1,0	0,9	0,7	1,1	1,2	0,9	1,2	0,8	1,1	1,1	1,2	1,3		2,9	0,6	0,8	0,9	0,8	1,1	1,4	Mdx+R+Prox1-TA-3G		
			Mdx+R+Prox1-TA-5G	1,1	4,2	1,6	1,9	0,8	1,2	1,2	0,9	0,7	0,9	0,9	1,0	0,6	0,6	1,0	1,1	0,8	1,3	0,8	0,9	1,0	1,7	0,7		1,4	0,4	0,8	0,8	0,6	1,2	1,4	Mdx+R+Prox1-TA-5G		
			Mdx+R+Prox1-TA-6G	0,9	2,8	0,9	1,6	0,9	1,6	0,8	0,7	1,1	0,6	2,3	1,0	1,9	1,0	1,0	1,0	0,8	0,7	0,9	0,9	1,0	1,0	0,6		0,7	0,8	1,0	1,2	1,1	0,1	2,1	Mdx+R+Prox1-TA-6G		
			Mdx+R+Prox1-TA-7G	1,0	2,9	0,8	0,8	1,1	1,0	1,3	1,1	0,7	0,6	1,0	0,9	0,6	0,7	1,1	1,0	0,9	1,2	0,9	1,2	1,4	1,9	0,5		2,1	0,6	0,8	1,1	0,7	1,8	1,1	Mdx+R+Prox1-TA-7G		
			Mdx+R+Prox1-TA-8G	1,1	5,2	0,9	1,2	1,0	0,9	0,9	0,8	0,9	0,8	1,0	0,9	0,5	1,0	0,9	0,8	0,7	0,9	0,9	0,9	1,1	0,9	0,3		1,2	0,5	0,9	0,7	0,6	1,3	1,6	Mdx+R+Prox1-TA-8G		
			Mdx+R+Prox1-TA-2D	0,7	1,9	0,4	0,7	1,0	1,5	0,8	0,8	1,2	0,6	1,3	0,9	1,2	0,9	0,9	0,8	0,8	0,7	0,8	0,7	0,9	0,7	1,4		1,6	0,9	0,9	0,9	1,0	0,4	1,8	Mdx+R+Prox1-TA-2D		
		Roue + Prox1	Mdx+R+Prox1-TA-1D	1,0	103,9	9,1	0,9	0,5	3,2	0,9	1,0	0,9	1,9	2,1	1,2	2,4	1,1	1,2	1,4	1,0	1,2	0,5	1,0	0,9	1,0	4,3		0,5	0,5	0,3	1,3	1,1	0,1	1,5	Mdx+R+Prox1-TA-1D	Roue + Prox1	
			Mdx+R+Prox1-TA-3D	0,9	149,7	11,9	1,3	0,8	2,5	1,1	1,1	0,9	1,6	1,4	1,2	0,8	1,0	1,2	1,6	1,1	1,3	0,7	1,1	1,2	1,7	2,4		3,0	0,6	0,4	1,0	0,8	0,7	1,3	Mdx+R+Prox1-TA-3D		
			Mdx+R+Prox1-TA-5D	0,8	134,0	48,6	1,9	0,5	3,1	0,6	0,8	0,8	1,7	2,1	1,2	1,2	1,0	1,0	1,2	1,1	0,8	0,5	0,9	1,0	1,4	1,3		0,8	0,4	0,4	1,1	0,7	0,2	2,3	Mdx+R+Prox1-TA-5D		
			Mdx+R+Prox1-TA-6D	1,0	82,0	15,1	2,6	0,5	2,6	0,8	1,0	0,7	1,5	1,2	1,1	0,4	0,5	1,0	1,1	0,9	0,8	0,8	1,2	1,4	1,6	0,8		0,8	0,5	0,5	0,8	0,5	0,6	2,0	Mdx+R+Prox1-TA-6D		
			Mdx+R+Prox1-TA-7D	0,8	164,3	18,7	0,9	0,5	2,8	0,8	0,9	0,9	1,8	1,5	1,0	1,1	1,4	1,1	1,1	1,1	1,2	0,6	1,0	1,7	1,6	1,4		1,1	0,4	0,4	0,9	0,6	0,7	1,5	Mdx+R+Prox1-TA-7D		
			Mdx+R+Prox1-TA-8D	0,8	164,4	24,7	0,9	0,5	1,9	0,8	0,9	0,8	2,0	1,7	1,1	1,1	0,6	1,1	1,3	1,0	1,0	0,7	1,1	1,9	1,4	1,0		0,9	0,7	0,5	1,0	0,7	0,5	1,5	Mdx+R+Prox1-TA-8D		
			Muscles	sdha	prox1	myh7	myh2	myh4	tnni1	snc4a	cacn1s	slc8a1	TRPC1	chrna1	prxII	gp91phox	p47phox	rac1	UTRN	desmin	murf1	Mafbox/atrogin1	LC3	gadd45	hdac4	Fn14		redd1	redd2	MSTN	follistatin	igf1	smox	sdha	Muscles		
					calcineurin signalling	type fibres				canaux ions avec dystro					NOX2	nadph oxydase					Atrophy													Ref			
