# Supplementary material for: The beneficial effect of chronic muscular exercise on muscle fragility is increased by Prox1 gene transfer in dystrophic mdx muscle
Source: PLoS One. 2022 Apr 18;17(4):e0254274. doi: 10.1371/journal.pone.0254274 (PMC9015141; doi:10.1371/journal.pone.0254274)
Supplement: Attachment — Submitted filename: Response to Reviewers.docx [file pone.0254274.s011.docx]

PONE-D-21-17707
The beneficial effect of chronic muscular exercise on muscle fragility is increased by Prox1 gene transfer in dystrophic mdx muscle
PLOS ONE

**Response to Reviewers**

**Academic Editor**

- A rebuttal letter that responds to each point raised by the academic editor and reviewer(s). You should upload this letter as a separate file labeled 'Response to Reviewers'. AUTHORS’RESPONSE:This was done.
- A marked-up copy of your manuscript that highlights changes made to the original version. You should upload this as a separate file labeled 'Revised Manuscript with Track Changes'. AUTHORS’RESPONSE: This was done.
- An unmarked version of your revised paper without tracked changes. You should upload this as a separate file labeled 'Manuscript'. AUTHORS’RESPONSE: This was done.

**Journal requirements**

When submitting your revision, we need you to address these additional requirements.

1. Please ensure that your manuscript meets PLOS ONE's style requirements, including those for file naming. The PLOS ONE style templates can be found at

<https://journals.plos.org/plosone/s/file?id=wjVg/PLOSOne_formatting_sample_main_body.pdf> and

<https://journals.plos.org/plosone/s/file?id=ba62/PLOSOne_formatting_sample_title_authors_affiliations.pdf>

AUTHORS’RESPONSE: We checked Plos One’s style requirements.

2. In your Data Availability statement, you have not specified where the minimal data set underlying the results described in your manuscript can be found. PLOS defines a study's minimal data set as the underlying data used to reach the conclusions drawn in the manuscript and any additional data required to replicate the reported study findings in their entirety. All PLOS journals require that the minimal data set be made fully available. For more information about our data policy, please see <http://journals.plos.org/plosone/s/data-availability>.

Upon re-submitting your revised manuscript, please upload your study’s minimal underlying data set as either Supporting Information files or to a stable, public repository and include the relevant URLs, DOIs, or accession numbers within your revised cover letter. For a list of acceptable repositories, please see [http://journals.plos.org/plosone/s/data-availability#loc-recommended-repositories](http://journals.plos.org/plosone/s/data-availability#loc-recommended-repositories). Any potentially identifying patient information must be fully anonymized.

Important: If there are ethical or legal restrictions to sharing your data publicly, please explain these restrictions in detail. Please see our guidelines for more information on what we consider unacceptable restrictions to publicly sharing data: [http://journals.plos.org/plosone/s/data-availability#loc-unacceptable-data-access-restrictions](http://journals.plos.org/plosone/s/data-availability#loc-unacceptable-data-access-restrictions). Note that it is not acceptable for the authors to be the sole named individuals responsible for ensuring data access.

We will update your Data Availability statement to reflect the information you provide in your cover letter.

AUTHORS’RESPONSE: Supporting information files concerning minimal data set were uploaded.

**Reviewer #1:**

 In this study, Monceau et al. investigated the effect of Prospero-related homeobox factor
1 gene (Prox1) transfer on fragility in chronically exercised or sedentary mdx mice. The authors concluded that Prox1 transfer reduced the force drop following lengthening contractions in exercised mdx mice, but not in sedentary mdx mice. Whereas, absolute maximal force and muscle weight were reduced by Prox1 transfer, especially in exercised mdx mice. In addition, based on the reduced muscle weight, the authors focused on atrophic genes.

Major concerns
1. The authors concluded the no effect of Prox1 transfer on the fragility and muscle weight in second experiments. In fact, there is no significant difference, but the data show the tendency to decrease. Is there a possibility that addition of mice changes the conclusion? The results of absolute maximal lengthening force (line 393-394) are also not significant, but actually the force was 20% decreased in Mdx+P group. AUTHORS’RESPONSE: Yes, it is possible. We have added this notion in the Discussion.

2. Lines 409-410; The present study confirms previous studies (8,18) showing that voluntary exercise alleviates the great susceptibility to contraction induced injury, a major dystrophic feature, in fast anterior crural muscles (TA and extensor digitorum longus) of mdx mice,
Lines 497-499; Combined to voluntary exercise, Prox1 transfer further improves (reduced) fragility, whereas the single Prox1 transfer approach in sedentary mdx mice failed to induce a
significant effect on the susceptibility to exercise-induced muscle injury.

Is muscle fragility really equal to susceptibility to muscle injury? In this study, there is no data indicating the reduced injury area in Mdx+W+P mice. AUTHORS’RESPONSE: The drop in maximal force following eccentric contractions is a marker recognized as relevant, emerging as a robust phenotype of murine dystrophy. This notion is underlined in the Introduction. It may give information different from that of histological markers. Indeed, it is possible to found a dramatic drop in maximal force for a few days although histological lesions are only minor.

3. The authors texted some genes related with muscle atrophy. However, in this study, there is no evidence indicating the decreased myofiber size. The decreased muscle size, but not muscle weight is primary criteria for muscle atrophy. AUTHORS’RESPONSE: This notion was added in the Discussion. The weight of the muscle is related to the volume of the muscle (weight = volume x density).


Minor concerns
1. Line 273; Please correct Figure 1C to Figure 2C. AUTHORS’RESPONSE: This is now revised.

2. Line 273-276; Immunohistological analyses revealed that these changes were not associated with the modification in the percentages of MHC-1, MHC-2a and MHC-2x expressing fibres because they were not different between Mdx+W+P muscle and Mdx+W muscle (Figures 2D and 2E).
Line 283-284; These data indicate that intramuscular delivery of AAV-283 Prox1 induced a substantial fast to slow contractile transition in the TA muscle of voluntary exercised Mdx mice.

It is unclear the definition of ‘substantial fast to slow contractile transition’ without the no changes of myofiber composition. AUTHORS’RESPONSE: The notion of a substantial rapid to slow transition is now revised.


3. To reviewer’s knowledge, Prox1 is well known as the marker and regulator for lymphangiogenesis. Reviewer recommends to add some description about the role of Prox1 in lymphangiogenesis. AUTHORS’RESPONSE: The importance of Prox1 for lymphatic vessels is now added (Introduction).

*************************************************************************************************************

**Reviewer #2:**

The authors investigated the effects of 1-month voluntary wheel-running exercise and/or Prospero-related homeobox factor 1 gene (Prox1) transfection in dystrophic mdx TA muscle. Prox1 transfection induced a fast-to-slow transition of the myosin isoform and reduced the lengthening-contraction-associated force decline in the TA. One month of running exercise was also found to partially attenuate the lengthening-contraction-associated force decline in the TA. This exercise-associated effect was enhanced by Prox1 transfection. The authors concluded that the Prox1-atransfection combined with voluntary exercise improves the fragility of the mdx muscle. While their results are fairly interesting, this manuscript has several concerns to address.

The authors report that the muscle weight was decreased by Prox1 transfection and/or 1-month voluntary wheel running. The authors should report the body weight, together with the muscle weight relative to the body weight, of each experimental group. These are important physiological parameters to evaluate the effects of Prox1 transfection and exercise. AUTHORS’RESPONSE: One leg is injected with Prox1 while the other leg is injected with PBS (Figure 1). One leg is injected with Prox1 while the other leg of the same mouse is injected with PBS (Figure 1). The body weights of the mice are therefore not different between the M + W + P and M + W groups. Ditto for groups M + P and M (Figure 1). Body weights is therefore not an interesting measured parameter. Body weights corresponding to M+W+P and M+W muscles were approximatively 30 g (28.g to 30.5 g), this was added in Materials and Methods.

While the muscle weight was decreased by Prox1 transfection and/or voluntary running, no significant difference was found in the mean fiber diameters of the respective fiber types among the experimental groups. What explains these discrepant results? Did these treatments decrease the number of muscle fibers? AUTHORS’RESPONSE: One possibility is that the number of fibers is decreased. Unfortunately, we could not measure this parameter with much precision because of the unsatisfactory quality of some cross sections of the muscle (part of the cross sections). Anyway, the possibility of a decrease in the number of fibers in 4 weeks is low because this type of phenomenon has not yet been described in previous studies. A second possibility is that the density of the muscle has changed (weight = volume x density). This latter notion is now added in the revised Discussion).

Prox1 transfection and/or voluntary exercise partially attenuated the lengthening-contraction-associated force decline in the TA. The relative reduction may be inversely related with the level of absolute maximal force. Do these results suggest that the lower level of maximal tension elicits the beneficial effects of Prox1 transfection and voluntary exercise on the fragility of the mdx TA muscle? Though the authors discuss this issue, they should take the added step of offering a clear explanation. The amplitude of the absolute maximal force had a large impact on the relative percentage of force reduction. Therefore, the authors should also evaluate the lengthening-associated force reduction using the absolute force. AUTHORS’RESPONSE: AUTHORS’RESPONSE: We added a clearer explanation in Discussion as suggested. Results concerning the reduction of maximal force are already presented in Results: “Similarly, absolute maximal lengthening force was lower (x 0.6) in Mdx+W+P muscle (157.2 g ± 7.5) compared to Mdx+W muscle (240.0 g ± 10.8) muscle (p < 0.01). In addition, the ratio of absolute maximal lengthening force to the absolute maximal isometric force was not different between Mdx+W+P muscle (1.9 ± 0.1) and Mdx+W muscle (1.8 ±329 0.1).”

Figure 2
An image of the mdx muscle should be provided. AUTHORS’RESPONSE: This is now added in supporting information files. We added all the images of muscles.
Judging from the images, the combination of Prox1 and voluntary exercise drastically increased the population of type 2x fibers, and decreased the populations of type 2a and 2b fibers, compared to the exercised-alone group. These changes are somewhat smaller than those shown in Figure 2C. What explains the difference? AUTHORS’RESPONSE: 1) When we measured all the images using immunohistochemistry, there are no differences in the number of fibers expressing neither MHC-2b nor MHC-2a between Mdx+W+P muscle and Mdx+W muscle, as shown in Figure 2E. We also found no significant difference between these two groups concerning the number of fibres expressing MHC-2x. 2) The variability between muscles and portions of muscle can explained the difference between Figure 2E and Figure 2D. So, based on the reviewer's remarks, we decided to delete Figure 2D. All the images of the muscles are now shown in supporting information files. 2) The discrepancy between the Figure 2C and Figure 2E could be explained by the fact that Figure 2C considers the co-expression of MHC, unlike Figure 2E. A second explanation could come from the fact that Figure 2C is more quantitative while Figure 2E is more qualitative. In the revised manuscript, we modified the notion of a substantial fast to slow transition since the effect of Prox1 on the populations on the different types of fibres is modest compared to what expected, and more complicated.

All of the evaluations in this study were carried out using the TA muscle. The fiber type distribution of the TA muscle is region-specific. The authors state that they counted all fibers in a cross-section of the mid-belly region of the TA. Figure 2D, on the other hand, shows only a limited portion of the TA muscle. AUTHORS’RESPONSE: We agree with the reviewer. We deleted the Figure 2D. The full images are now shown in supporting information files.

The cause of the lengthening-associated force reduction remains unclear. The intracellular Ca2+ level has an impact on the force generation. The authors should provide a record of the force curve with a baseline level (namely, the changes in the resting tension during successive contractions). AUTHORS’RESPONSE: We found no change in the baseline level. Force records are now shown supporting information files.

Further, they should evaluate the molecules that handle intracellular Ca2+, such as those associated with the functions　sarcoplasmic reticulum (ryanodine receptor, Ca2+ pump), T-tubes (DHP receptor, caveolin), parvalbumin, and Na+-Ca2+ exchanger. AUTHORS’RESPONSE: We agree with the reviewer, the study of the functions of the proteins involved in excitation would be very interesting, but this goes well beyond the scope of this study. Previous studies (cited in the manuscript) show that treatments (gene therapy aimed at restoring dystrophin expression, voluntary exercise) that improve muscle fragility in mdx mice improve muscle excitability, through mechanisms not still well known

While revising your submission, please upload your figure files to the Preflight Analysis and Conversion Engine (PACE) digital diagnostic tool, <https://pacev2.apexcovantage.com/>. PACE helps ensure that figures meet PLOS requirements. To use PACE, you must first register as a user. Registration is free. Then, login and navigate to the UPLOAD tab, where you will find detailed instructions on how to use the tool. If you encounter any issues or have any questions when using PACE, please email PLOS at [figures@plos.org](mailto:figures@plos.org). Please note that Supporting Information files do not need this step. AUTHORS’RESPONSE: The new Fig2 was uploaded to the PACE tool.
