# Supplementary material for: The beneficial effect of chronic muscular exercise on muscle fragility is increased by Prox1 gene transfer in dystrophic mdx muscle
Source: PLoS One. 2022 Apr 18;17(4):e0254274. doi: 10.1371/journal.pone.0254274 (PMC9015141; doi:10.1371/journal.pone.0254274)
Supplement: Attachment — Submitted filename: Response 2.docx [file pone.0254274.s012.docx]

**Reviewers' comments:**

**Reviewer #1:**

Related to major comment #1

Authors’ response; Line 462; It is nevertheless not excluded that the addition of mice changes this conclusion.

AUTHOR RESPONSE. Major comment #1. Previously, to consider the comment of the reviewer we had indeed added in the Discussion this :“It is nevertheless not excluded that the addition of mice changes this conclusion”. In the revised manuscript we added: “but the potential beneficial effect would nevertheless be less important” (see below).

Additional experiment should be performed to confirm the possibility.

AUTHOR RESPONSE. “Additional experiment should be performed to confirm the possibility”. The principal purpose of the present study was to determine whether Prox1 transfer, i.e., Prox1 transfer, reduced muscle fragility in voluntary exercised mdx mice, since voluntary running only partly reduced the susceptibility to exercise-induced muscle injury, so it would be interesting to combined the effects of exercise with those of another treatment, i.e., Prox1 transfer. Thus, the main aim is not to study the effect of the transfer of Prox1 in sedentary mdx mice. This study already includes 2 sets of experiments. In our opinion, these 2 sets of experiments already bring important preclinical results in exercised mdx mice, which meets our objectives. However, we agree that it may be of interest in the future to better study the effect of Prox1 transfer in mdx mice, although the effect appears to be more modest. In fact, the p-value is high (p = 0.3989) in the second set of experiment. In order to find a significant difference (p = 0.05) between the 2 groups (sedentary mice treated or not with Prox1), we estimate (calculate) the number of muscles in each of the 2 groups to be more than 20. Unfortunately, it not possible to perform and analyze a such study. It is not possible to do such a third set of experiment, with so many muscles in each group at the same time. Indeed, it would take too much production of mice (only one sex is studied) and viral vector. In addition, the difference of 8.623% between the 2 groups (Mdx + P and Mdx) is small, of no great physiological consequence, even if it had to be found statistically significant using 40 more muscles.

Related to major comment #2

Reviewer’s question means that the impact of Prox1 on susceptibility to muscle injury, because the authors described this concerns in line 409-410 (revised 403-404) and 497-499 (revised 492-494). Without the results of histological analyses, the authors can not mention the impact of Prox1 on susceptibility to muscle injury. The description in lines 492-494 also needs to be correct as sedentary group did not exercise.

AUTHOR RESPONSE: “Muscle injury”. The immediate drop force following lengthening contractions we used is a very widely accepted way to determine the susceptibility to exercise-induced muscle damage (see Introduction). Below are three examples: 1) “Lack of dystrophin renders skeletal muscle susceptible to injury, particularly eccentric contraction (ECC)-induced strength loss.” (Skeletal Muscle. 2020; 10: 3.), 2) “Susceptibility to ECC contraction-induced strength loss has therefore become a standard outcome measure in preclinical studies to assess disease severity and the efficacy of potential therapies for DMD.” (Med Sci Sports Exerc. 2020 Feb;52(2):354-361) and 3) “Whether whole muscles are studied in vitro, in situ, or in vivo, the overwhelming evidence indicates that whole skeletal muscles of mdx mice show a greater susceptibility to contraction-induced injury than muscles of control mice. The most compelling data indicate a 20% greater force deficit for EDL muscles of mdx mice compared with those of control mice with both studied in situ.”(Am J Physiol Cell Physiol. 2000 Oct;279(4):C1290-4.). It is this notion which is defined at the beginning of the Introduction of our manuscript, and which we use in our manuscript. Moreover, in a recent study, we found no histological structural change following immediate lengthening contractions in mdx mice (Roy et al 2016, Skeletal Muscle. 2016 Jul 20;6:23.). This last notion was also added in the revised manuscript (Introduction), in order to better define what fragility is. Moreover, we have considered the reviewer's remark in our revised manuscript (Abstract, Discussion, conclusion…). In particular, we have removed the notion of injury.

AUTHOR RESPONSE. “The description in lines 492-494 also needs to be correct”. Mdx mice (sedentary Mdx mice) did not perform chronic voluntary running (see Material and Methods) but their TA muscles performed 9 lengthening contractions. Please, but do not confuse the single session of 9 lengthening contractions in response to nerve electric stimulation with chronic voluntary running in a wheel. The 9 lengthening contractions are performed to evaluate the “Susceptibility to ECC contraction-induced strength loss”, fragility.

Related to major comment #3

Reviewer disagrees with the authors’ response because number of myofiber also affect the muscle wight. As there is not difference in the data of diameter, the data of myofiber size should be included.

AUTHOR RESPONSE. “the data of myofiber size should be included”. The data of myofiber size were already included in the manuscript R-1 (Figures 4D and 6H). However, the data of diameter are now deleted in the R-2 manuscript because Reviewer #2 have criticisms regarding the quality of the immunostained image. This is the reason why we prefer to not show data concerning the number of fibers in the previous version of the manuscript, which requires a perfect quality of muscle cross section images, which is difficult to obtain in dystrophic mice with many muscle tissue alterations (very small fibres and cells, splitted fibers, branched fibers…), in addition to the absence of dystrophin which did not allow a good staining of the contours of the fibers. For example, half of the fibers were branched in young adult mdx mice (Front Physiol. 2021 Dec 7;12:771499.). Thus, we deleted all histological data in the revised manuscript. This is not too regrettable as the effect of the transfer of Prox1 on fibre size/number is clearly not the main objective of the study. The main objective is to study the effect of the transfer of Prox1 on the muscle fragility of exercised mdx mice. In order to improve this understanding, we have focused less on muscle weight reduction in the revised manuscript.

**Reviewer #2:**

The authors made a few slight modifications to their manuscript in response to my previous comments. Regrettably, their responses and revisions do not go far enough. While the authors added supplemental data, their other revisions were minimal. The quality of the cross-sectional images of the muscle is a particular concern.

The authors’ responses and explanations on the discrepancy between their results on the muscle weights and fiber CSAs were insufficient. Both increases in number of muscle fibers and density of muscle fibers are unlikely. This finding can be attributed to technical errors during the fixation, the sectioning, and/or the staining procedures. Judging from the supplemental data (S4-S9), the very low quality of the images prevented the authors from accurately determining the fiber CSAs. These poor images reflect the low quality of the data in this study overall.

AUTHOR RESPONSE. “the very low quality of the images prevented the authors from accurately determining the fiber CSAs”. We agree with the reviewer, the quality of the immune-stained images is not good, probably due to the freezing/preservation of the muscles and structural alterations in dystrophic muscle. We regularly encounter difficulties to obtain nice images in dystrophic mice with many muscle tissue alterations (very small fibres and cells, splitted fibers, branched fibers, fibrosis…), in addition to the absence of dystrophin which did not allow a good staining of the contours of the fibers. For example, half of the fibers were branched in young adult mdx mice (Front Physiol. 2021 Dec 7;12:771499.). As suggested by the reviewer, we deleted all histological data. This is not too regrettable as the effect of the transfer of Prox1 on fibre size/number is clearly not the main objective of the study. The main objective is to study the effect of the transfer of Prox1 on the muscle fragility of exercised mdx mice. In order to improve this understanding, we have focused less on muscle weight reduction in the revised manuscript.

As mentioned, the quality of the immuno-stained images is too low. As a reviewer, I am unable to judge the MHC type of each fiber. I believe that types 2a and 2b are co-expressed in many types of fibers. The authors should carry out staining on new sections and then show not only merged images but also separate images stained with each type of MyHC.

AUTHOR RESPONSE.” I believe that types 2a and 2b are co-expressed in many types of fibers”. We found that very few MHC-2a and MHC-2b are co-expressed (Mdx: 0-10 fibres per TA muscle cross-section; Mdx+W: 1-18 fibres; Mdx+W+P: 2-3 fibres). We also made separate images. To take this remark (low quality of the image) into account, we have deleted all data concerning immunohistology. This is not harmful because we have other markers of fast to slow conversion of fiber type.

Figures 3 and 4:

The authors should show the body weights of the mdx mice. They should also evaluate the difference in body weights between the mdx group and Prox1-transferred mdx group with or without exercise.

AUTHOR RESPONSE. “Difference in body weights”. The range of the body weights of mdx mice (set 1) were already shown. We now shown the mean values ± SEM of the 2 groups (29.9 ± 0.321 versus 31.4 ± 0.23 g, see Materials and methods). There a 4.9% difference in body weight between the 2 groups (mdx group and Prox1-transferred mdx group with or without exercise). The mdx group was not intramuscularly injected, did not voluntary run and was studied 3-4 weeks later (The mdx group was studied later, due to sanitary confinement).

Why, moreover, did the muscle weight decrease by ~25% following the Prox1 gene transfer only during the 4-week experimental period? And why did the weights of the mdx mice with both Prox1 and exercise decrease by ~50% compared to the mdx control during the 4-week experimental period? Was this reduction of muscle weight attributable to atrophy? Were physiological factors responsible for the abnormal changes in muscle weight induced by Prox1?

AUTHOR RESPONSE. “Why, moreover, did the muscle weight decrease by 25%”. As described in the experimental design (Figure 1), the muscles treated or not with Prox1 come from the same mice. Therefore, they were subjected to the same physiological factors. The only difference is the injection of AAV-Prox1. Either it is Prox1 itself which is responsible for the reduction in muscle weight, or it is the injection of the AAV itself (independent of the overexpression of Prox1). This was now indicated in the text of the revised manuscript (see Discussion). What we do know is that a decrease in muscle weight of sedentary Mdx is also observed following various treatments inducing a change in the type of fibers (see Discussion). We agree with the reviewer that it would be interesting to better analyze the effects of Prox1 transfer on muscle weight. Unfortunately, we have no clear explanation concerning the reduction in the weight of the muscles treated with AAV-Prox1. However, for us, what is important in the results of this preclinical study, with regard to the objective of this study, is that a transfer of the Prox1 gene improves the fragility of the exercised dystrophic muscle, but also decreases the absolute maximal force. Thus, the potential clinical benefit of the transfer of Prox1 into exercised dystrophic muscle is real but more questionable than expected.

AUTHOR RESPONSE. “why did the weights of the mdx mice with both Prox1 and exercise decrease by ~50%”. Concerning the reduced muscle weight of Mdx+W+P compared to Mdx mice we noted these differences between the 2 groups of mice: 1) the Mdx group was not intramuscularly injected with AAV-Prox1, 2) Mdx mice did not voluntary run and 3) Mdx mice were studied 3-4 weeks later (the Mdx group was studied later, due to sanitary confinement). That makes a lot of differences that make a possible explanation even more complicated.
