# Supplementary material for: The beneficial effect of chronic muscular exercise on muscle fragility is increased by Prox1 gene transfer in dystrophic mdx muscle
Source: PLoS One. 2022 Apr 18;17(4):e0254274. doi: 10.1371/journal.pone.0254274 (PMC9015141; doi:10.1371/journal.pone.0254274)
Supplement: Attachment — Submitted filename: Response to reviewers 4.docx [file pone.0254274.s013.docx]

**Reviewers' comments:**

While the focus of this study is for functional improvement of mdx muscle after injection of Prox1 AAV vectors, both reviews requested revised histological figures as well as fiber CSA measurements, both of which data would strengthen the mdx muscle phenotype after Prox1 gene transduction. Therefore, please add revised histological figures and perform the fiber CSA measurements.

Author response: As requested, we add revised histological figures concerning the measurement of fibre size. In fact, we preferred to measure the smallest diameter (min feret) of the fibres (and not fibre CSA) because some fibers are not cut transversely. We have selected the images of muscle cross-section that are of good quality, in order to respond to criticism from reviewers in previous reviews. We were not able to recut the muscles to make new muscle cross-sections because we had already used all of the muscles before for analysis (immunobloting, qPCR, histology). With these good quality images, we measured the min ferret of the fibres. All these selected images are shown in Supplemental data.
